# Single-cell RNA-based phenotyping reveals a pivotal role of thyroid hormone receptor alpha for hypothalamic development

**DOI:** 10.1242/dev.201228

**Published:** 2023-01-30

**Authors:** Varun K. A. Sreenivasan, Riccardo Dore, Julia Resch, Julia Maier, Carola Dietrich, Jana Henck, Saranya Balachandran, Jens Mittag, Malte Spielmann

**Affiliations:** ^1^ Institute of Human Genetics, Universitätsklinikum Schleswig-Holstein, University of Lübeck and University of Kiel, Lübeck 23562, Germany; ^2^ Institute for Endocrinology and Diabetes, University of Lübeck and Universitätsklinikum Schleswig-Holstein Campus Lübeck, Center of Brain Behavior and Metabolism (CBBM), Ratzeburger Allee 160, 23562 Lübeck, Germany; ^3^ Human Molecular Genomics Group, Max Planck Institute for Molecular Genetics, Berlin 14195, Germany; ^4^ German Centre for Cardiovascular Research (DZHK), partner site Hamburg/Lübeck/Kiel, Lübeck 23562, Germany

**Keywords:** Hypothalamus, Single-cell sequencing, Hypothyroidism, Thyroid receptor, Oligodendrocyte, Mouse

## Abstract

Thyroid hormone and its receptor TRα1 play an important role in brain development. Several animal models have been used to investigate this function, including mice heterozygous for the TRα1R384C mutation, which confers receptor-mediated hypothyroidism. These mice display abnormalities in several autonomic functions, which was partially attributed to a developmental defect in hypothalamic parvalbumin neurons. However, whether other cell types in the hypothalamus are similarly affected remains unknown. Here, we used single-nucleus RNA sequencing to obtain an unbiased view on the importance of TRα1 for hypothalamic development and cellular diversity. Our data show that defective TRα1 signaling has surprisingly little effect on the development of hypothalamic neuronal populations, but it heavily affects hypothalamic oligodendrocytes. Using selective reactivation of the mutant TRα1 during specific developmental periods, we find that early postnatal thyroid hormone action seems to be crucial for proper hypothalamic oligodendrocyte maturation. Taken together, our findings underline the well-known importance of postnatal thyroid health for brain development and provide an unbiased roadmap for the identification of cellular targets of TRα1 action in mouse hypothalamic development.

## Teaser

**Summary:** Unbiased phenotyping of the mouse hypothalamus using single-cell RNA sequencing reveals the crucial role of early postnatal thyroid hormone receptor α1 signaling for hypothalamic oligodendrocyte maturation.

## INTRODUCTION

Thyroid hormone plays an important role in brain development. Clinically, this becomes most visible in untreated congenital hypothyroidism, which leads to irreversible mental retardation (Grüters and Krude, 2011). The effects of thyroid hormone are mediated by two different receptor isoforms, alpha and beta. For brain development, thyroid hormone receptor α1 (TRα1) is the most relevant isoform (Yen, 2001), present in almost all neurons (Wallis et al., 2010; Yen, 2001). To understand the effects of thyroid hormone and TRα1 on brain development, several animal models have been established (Flamant and Quignodon, 2010). Among those are mice harboring a heterozygous R384C mutation in TRα1 (TRα1+m). This particular mutation has been selected as it does not abolish thyroid hormone signaling, but instead only reduces the affinity of the receptor to the ligand to one-tenth, which has the decisive advantage that TRα1 signaling can be reactivated *in vivo* at any time by pharmacologically increasing the circulating levels of thyroid hormone (Tinnikov et al., 2002). Using this mouse strain, acute defects in thyroid hormone signaling can be distinguished from defects that occur during development by reactivating TRα1 signaling in the respective periods (Harder et al., 2018; Pedaran et al., 2021). For example, it was revealed that hypothalamic parvalbumin neurons require intact TRα1 signaling in the second half of pregnancy for correct development, but they are independent of thyroid hormone postnatally (Harder et al., 2018). However, little is known about the role of TRα1 signaling for the development of other hypothalamic cell populations.

Single-cell genomics approaches offer powerful tools to quantify cellular composition changes and gene expression alterations in any tissue, organ or entire organism (https://www.humancellatlas.org/) (Cao et al., 2019; Cao et al., 2020; Huang et al., 2022 preprint; Smajić et al., 2022). With regard to the hypothalamus, several studies have been conducted on wild-type mice using this technique, but these reports focus largely on neurons (Campbell et al., 2017; Chen et al., 2017; Hajdarovic et al., 2022; Kim et al., 2020; Mickelsen et al., 2019; Moffitt et al., 2018; Romanov et al., 2017, 2020; Steuernagel et al., 2022).

In our current study, we set out to use single-cell RNA sequencing on hypothalami of TRα1+m mutant mice and respective controls to obtain an unbiased global view of the role of TRα1 in hypothalamus development, cell-type diversity and the cellular transcriptome. We show that defective TRα1 signaling has surprisingly little effect on the development of hypothalamic neuronal populations. In contrast, it heavily affects the development of hypothalamic oligodendrocytes, which seem to require TRα1 signaling for full maturation. Using selective reactivation of the mutant TRα1 in our animal model, we subsequently demonstrate that early postnatal thyroid hormone action is crucial for the development of this cell type.

## RESULTS

### Cell-type composition of the hypothalamus

**Fig. 1.**
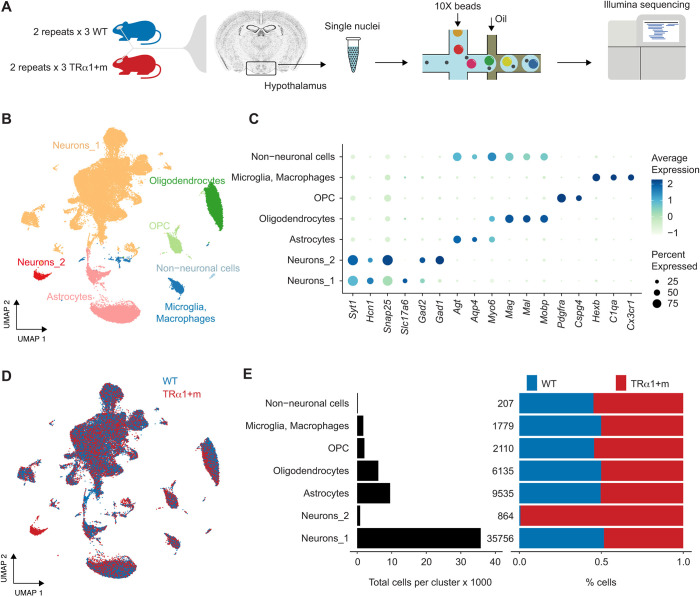
**Experimental scheme and the cell-type composition of the murine hypothalamus.** (A) Experimental scheme of hypothalamus tissue processing, sample pooling, single-nuclei barcoding, and sequencing. (B) UMAP embedding of 56,386 nuclei, colored by cell-type annotations. (C) Differentially expressed genes, based on which the clusters in B were annotated. (D) Same UMAP embedding as in B, but colored based on the genotype. (E) Cell-type composition (left) and the percentage contribution of nuclei in clusters from each of the two genotypes, after correcting for the total number of nuclei in the two genotypes (right).

Hypothalamus tissues were collected from six adult wild-type and six TRα1+m mice and snap-frozen in liquid nitrogen (Fig. 1A). Three tissues within each genotype were pooled together, yielding four samples of nuclei suspensions that were processed for sequencing, which ultimately resulted in ∼12,000-16,000 nuclei per sequenced sample. The sequencing output contained a median of ∼3200 transcripts (unique molecular indices) and ∼1800 distinct genes per nucleus after filtering out poorly sequenced nuclei, nuclei with abnormally high mitochondrial and ribosomal gene counts, and nuclei predicted to be doublets (Materials and Methods). The final filtered dataset comprised 27,975 and 28,411 nuclei from the wild-type and the TRα1+m mice, respectively.

First, we projected the nuclei in the feature-space into a principal component space to perform clustering and visualization, which was then further projected down to two-dimensional uniform manifold approximation and projection (UMAP) embeddings to aid visualization (Fig. 1B). This revealed seven main clusters, which were unique primarily based on known cell types in murine hypothalamus, namely neurons, astrocytes, oligodendrocytes, oligodendrocyte progenitor cells (OPCs) and microglia/macrophages. These cell types were identified based on the known marker genes *Slc17a6* and *Gad1* (neurons), *Agt*, *Aqp4*, *Slc1a2* and *Slc1a3* (astrocytes) (Hajdarovic et al., 2022), *Mag* and *Mobp* (oligodendrocytes) (Hajdarovic et al., 2022), *Pdgfra* and *Cspg4* (OPCs) (Marques et al., 2016), and *Hexb*, *C1qa* and *Cx3cr1* (microglia/macrophages) (Campbell et al., 2017) expressed by these clusters, as revealed by differential gene expression analysis, and was in agreement with the previously reported cell types in murine hypothalamus (Campbell et al., 2017). Because the astrocyte cluster appeared to be composed of two groups of cells in the UMAP embedding, we performed sub-clustering, which revealed the presence of two additional cell types, namely *Nnat^+^* tanycytes, which are specialized ependymal cells lining the third ventricle, as well as *Igfbp2^+^* vascular and leptomeningeal cells (VLMCs), in addition to astrocytes. (Fig. S1). These annotations were verified based on the top differentially expressed genes using the enrichment analysis tool Enrichr (Chen et al., 2013; Kuleshov et al., 2016; Xie et al., 2021) (Fig. 1C). The small seventh cluster, containing 207 cells, appeared to express several astrocytic and oligodendrocytic marker genes and was initially annotated to be the non-neuronal cell cluster. Specifically, of the 929 positively differentially expressed genes in this non-neuronal cluster, 408 and 500 genes were shared with the differentially expressed genes of oligodendrocytes and astrocytes, respectively. A similar cluster was also observed in the lateral hypothalamic area by Mickelsen et al. (figure S2 in Mickelsen et al., 2019), which was annotated as doublets in their dataset. Indeed, these cells did exhibit a higher doublet score distribution in our dataset (Fig. S2). Although the formation of such cell type-specific doublets constituting astrocytes and oligodendrocytes is unlikely in our dataset owing to the use of single-nuclei sequencing (as opposed to single-cell sequencing by Mickelsen et al.), we erred on the side of caution and decided not to analyze this small cluster further (0.37% of total nuclei).

Next, we performed a cell-type composition analysis that revealed neurons to be the most abundant cell type in the murine hypothalamus, followed by astrocytes and oligodendrocytes (Fig. 1E, left). Segregating the cell-type composition by genotype showed that all the cell types, except Neurons_2, were composed equally of wild-type and TRα1+m cells, suggesting no major changes to the hypothalamic neuronal composition and the transcriptomic profiles in the mutant mice (Fig. 1D, Fig. 1E, right). Segregating the cell-type composition by the experimental repeats revealed that the Neurons_2 cluster contained nuclei primarily from the second experimental replicate (853 nuclei in repeat #2 versus 11 nuclei in repeat #1), and was therefore deemed to be an experimental artifact and not included for further analysis.

As a final check, we verified whether batch effects between the two experimental replicates (Fig. S3) could have influenced our cell-type annotation, or have resulted in the misleading identification/annotation of the Neurons_2 cluster (composed mostly of repeat #2). To this aim, we performed a harmony-based batch correction, followed by dimensionality reductions, clustering, differential gene expression analysis, and cell-type annotation (Fig. S4). Harmony was chosen for batch correction based on our preliminary comparison of its performance with that of Seurat's canonical correlation analysis as well as the published benchmarking results on batch-correction methods (Tran et al., 2020). A comparison of the cell-type annotation between the raw and batch-corrected data revealed that the annotation of only 30 of the 56,386 cells changed as a result of batch correction, indicating that the batch effects in the data set did not significantly affect coarse-level clustering (Fig. S4D,E).

Overall, the cells of wild type and TRα1+m appeared to be scattered throughout the UMAP embedding (before and after batch correction) without genotype-specific clusters, suggesting no major differences in cellular composition between the two genotypes at least in terms of the main cell types (Fig. 1, Fig. S4). Next, we explored the dataset for finer differences in cellular subtypes as well as in gene expression profiles.

### Neurons exhibit only minor differences between the genotypes

**Fig. 2.**
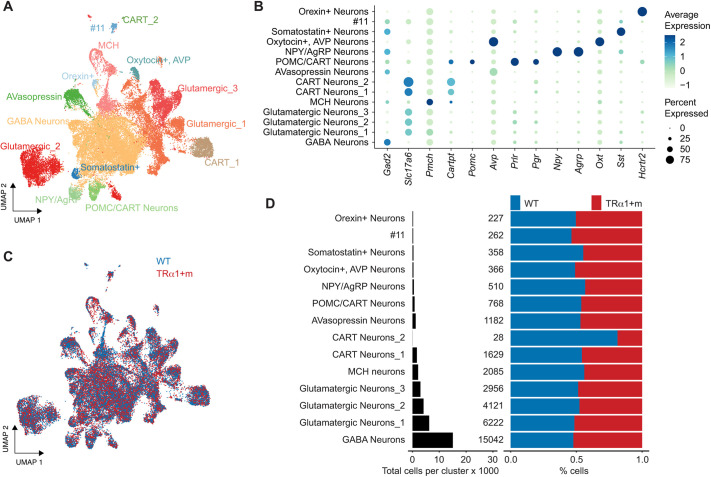
**Sub-clustering of Neurons_1, annotation and cell-type composition.** (A) UMAP embeddings of the neuronal cluster after harmony-based batch correction across the two experimental repeats (Fig. 1A, Neuron_1). (B) Differentially expressed genes, based on which the sub-clusters were annotated. (C) Same UMAP embedding as in A, but colored based on the genotype. (D) Cell-type composition (left) and the percentage contribution of nuclei in each cluster from each of the two genotypes, after correcting for the total number of nuclei in the two genotypes (right).

Given that the neuronal composition is known to be affected in the TRα1+m mutants (Mittag et al., 2013), we asked whether we could replicate this finding in the single-cell sequencing data and if there were other changes to the neuronal composition that could not be seen at coarse-level analysis or by traditional histological approaches. To answer these questions, we aimed to sub-cluster the Neurons_1 cluster in order to annotate the neuronal subtypes. However, a UMAP embedding of Neurons_1 segregated by the experimental repeats revealed that the cells from the two repeats were distinctly located in the embedding, likely a result of batch effects (Fig. S5B). To remove the technical differences, we subjected the cells to harmony-based batch correction across the two repeats, followed by clustering, resulting in 14 sub-clusters corresponding to the various neuronal subtypes. GABA neurons, glutamatergic neurons, melanin-concentrating hormone (MCH) neurons, cocaine- and amphetamine-regulated transcript (CART) neurons, agouti-related peptide (NPY/AgRP) neurons, arginine vasopressin (AVasopressin) neurons and pro-opiomelanocortin (POMC) neurons, among others, were identified based on known hypothalamus neuronal subtypes and their markers (Fig. 2A-C).

To address the first question, as to whether parvalbuminergic neurons are less abundant in the TRα1+m hypothalamus (Mittag et al., 2013), we identified these cells based on the expression of *Pvalb* and *Syt2*, known markers of parvalbuminergic neurons (Sommeijer and Levelt, 2012) (Fig. S6A-D). Indeed, the total number of parvalbuminergic neurons dropped from 66 in the wild-type hypothalamus to 16 in the mutant hypothalamus, which, after correcting for the total number of neurons per dataset, amounts to a drop in abundance by 72% (Fig. S6E). However, given the generally low abundance of these neurons and the limited experimental repeats, the difference in cell numbers was not found to be statistically significant. Perhaps for similar reasons, a differential gene expression analysis between the genotypes for this neuronal population did not yield any differentially expressed genes that met our statistical filtering criteria (see Materials and Methods).

To address the second question, as to whether the perturbed thyroid hormone signaling affected other neuronal subtypes, we performed cell-type composition analysis across all the neuronal sub-clusters, which, when segregated based on genotype, revealed no discernable differences between the wild-type and TRα1+m cells. Only a small cluster of 28 CART Neurons_2 deviated from ∼50% (Fig. 2D). Differential gene expression analysis (Tables S2) between the two genotypes across all neuronal sub-clusters revealed seven genes (after additional *P*-value-based filtering), of which *AC149090.1* (phosphatidylserine decarboxylase, *Pisd*) was present in eight of the 14 neuronal sub-clusters and was also the only gene that was expressed higher in the TRα1+m than the wild-type neurons. The other six appeared in only one (*Grm8*, *Hcn1*, *Lrrtm4*, *mt-Atp6* and *Oxt*) or two (*mt-Co3*) sub-clusters each, and to our knowledge have not been linked to thyroid hormone signaling previously, whereas *Pisd* plays a role in lipid metabolism, a pathway known to be affected by thyroid signaling in the hypothalamus (López et al., 2010; Pucci et al., 2000). Overall, based on the gene expression data and the similar expression of several neuronal markers tested by *in situ* hybridization and immunohistochemistry, including *Agrp*, *Npy*, *Pomc*, *Crh*, *Hcrt*, *Gad67* (*Gad1*) or NeuN (Fig. S7), the effect of the mutant TRα1 on hypothalamic neuronal development seems to be subtle.

### Oligodendrocytes are affected by disrupted thyroid receptor function

**Fig. 3.**
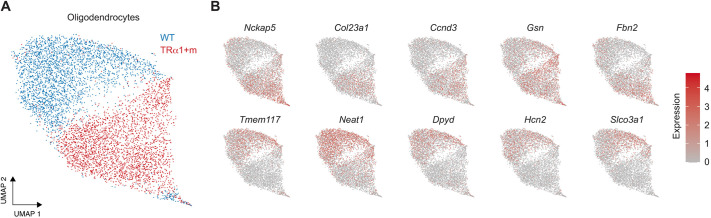
**UMAP embedding of oligodendrocytes and differential expression between the two genotypes.** (A) UMAP embedding of oligodendrocytes after harmony-based batch correction across the two experimental repeats. (B) Expression pattern of a subset of genes that were in the top ten differentially expressed genes between the two genotypes. Violin plots of all differentially expressed genes are presented in Figs S11, S12 and S13 and the corresponding raw data are available in Table S2.

We performed similar harmony-based batch correction and UMAP embedding of all the cell types (Fig. 3A, Fig. S8, S9). Amongst them, oligodendrocytes (Fig. 3A) and, to a lesser extent, astrocytes and oligodendrocyte progenitor cells (Fig. S9A,B) exhibited segregated accumulation of cells from the two genotypes in the UMAP embeddings. Differential gene expression analysis between the two genotypes across all the annotated clusters and sub-clusters also revealed oligodendrocytes to be the most affected cell type, with 99 differentially expressed genes after filtering (see Materials and Methods; Fig. S10, Table S2). Of the 99 differentially expressed genes identified, 66 genes were expressed higher and 33 genes were expressed lower in the wild type compared with the mutant. The expression pattern of a subset of these genes that showed the most difference in the percentage of cells expressing them are presented in Fig. 3B and the violin plots of all the identified genes are shown in Figs S11, S12 and S13.

### Oligodendrocytes require early postnatal TRα1 signaling for correct development

**Fig. 4.**
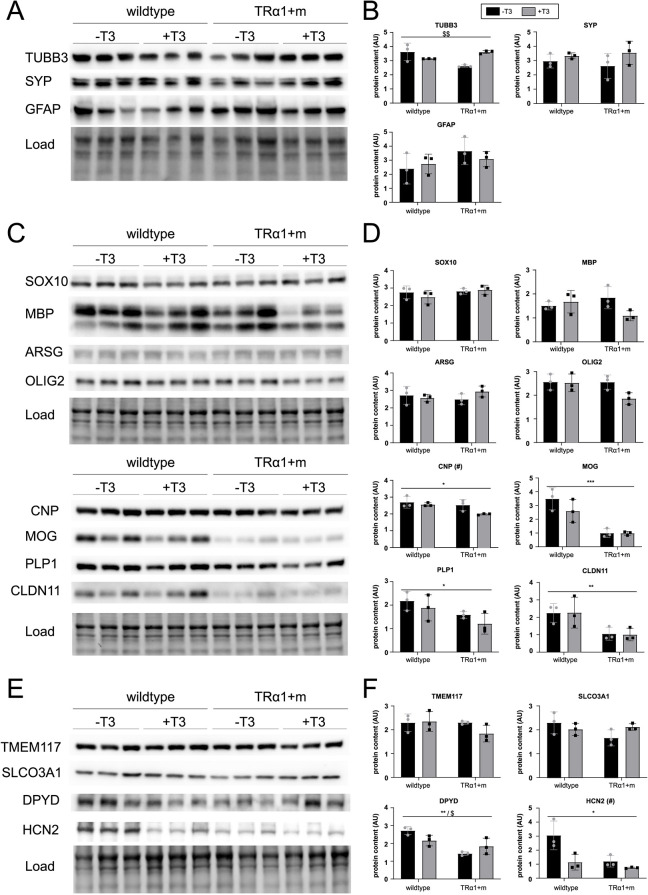
**Alterations in oligodendrocytic proteins in the TRa1+m hypothalamus.** (A-F) Comparison of the protein-expression of cellular and oligodendrocyte markers from whole-hypothalamus homogenates in adult wild type and TRα1+m mutant, with and without T3 treatment. Raw western blots (A,C,E) and their quantifications (B,D,F) for the expression of overall hypothalamic cell markers (A,B), classic oligodendrocyte markers (C,D) and four potentially differentially expressed genes between the WT and the mutant (E,F). *P<0.05, **P<0.01, ***P<0.001 for wild type versus TRα1+m mutant; ^#^*P*<0.05 for T3-treatment; ^$^*P*<0.05, ^$$^*P*<0.01 for interaction between T3 treatment and genotype (two-way ANOVA, *n*=3 per group, Table S1). Data are mean±s.d. AU, arbitrary unit.

Given that oligodendrocytes seemed to be the most severely affected cell population in TRα1+m mutant mice, we focused on this cell type for a more in-depth analysis. Because mRNA translation is heavily regulated in oligodendrocytes on several levels, including mRNA editing (Xu et al., 2020), integrin-mediated activation of the 3′UTR (Laursen et al., 2011), mRNA transport (Müller et al., 2013), tRNA modification and codon-based mRNA decay (Martin et al., 2022) as well as small non-coding RNAs (Bauer et al., 2012), we decided to probe for established oligodendrocyte proteins from different stages of development to better define the fate of this cell type, namely OLIG2 and SOX10 for all stages of development; PLP1 and CNP for stages from pre-oligodendrocytes; and MBP, MOG, CLDN11 (also known as oligodendrocyte-specific protein) and ARSG for mature oligodendrocytes. We therefore included an additional cohort of mice from both genotypes that were treated for 2 weeks as adults with thyroid hormone T3 in drinking water to restore acute TRα1 signaling. Although SOX10, MBP, ARSG and OLIG2 were not affected by genotype or T3 treatment (Fig. 4, Table S1), we observed a reduction of CNP, MOG, PLP1 and CLDN11 in the TRα1+m mice, which was, however, not restored when TRα1 was reactivated by T3, indicating a permanent developmental defect. The newly identified differentially expressed genes *Tmem117*, *Dpyd*, *Slco3a1* and *Hcn2* (Fig. 3) were also tested at the protein level, confirming a reduction in DPYD and HCN2 that was not rescued by T3 treatment, whereas TMEM117 and SLCO3A1 were not affected at the protein level in either condition (Fig. 4, Table S1).

Based on single-cell expression, we subsequently verified that the protein expression of these marker genes measured in tissue homogenates provided a reasonable representation of their expression in oligodendrocytes (Fig. S14A). However, the clear differences observed between the genotypes in CNP, MOG, PLP1 and CLDN11 protein levels were not recapitulated at the level of transcript expression, probably owing to the strong translational regulation mentioned earlier (Fig. S14B). Taken together, these findings led us to conclude that oligodendrocytes do develop in TRα1+m mice, but seem to have different properties, which cannot be restored by adult reactivation of TRα1 signaling.

**Fig. 5.**
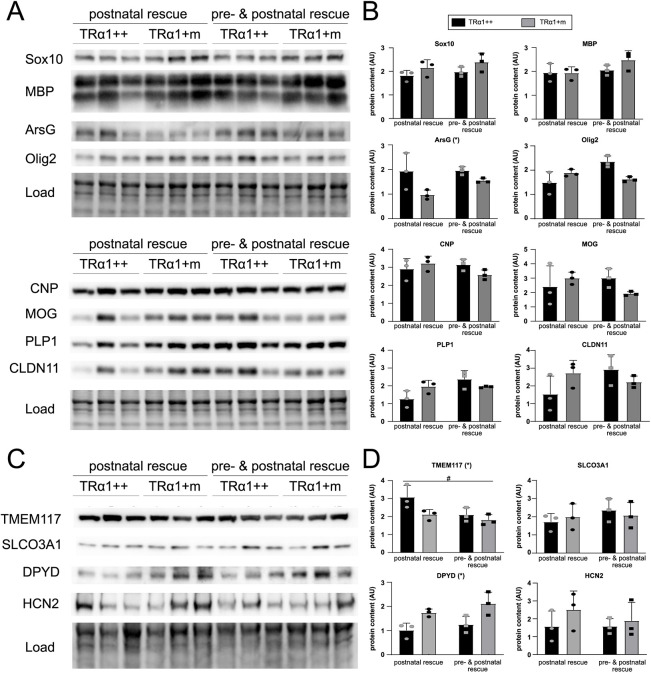
**Oligodendrocyte protein levels are rescued in TRa1+m hypothalamus by early postnatal thyroid hormone.** (A-D) Comparison of the protein-expression of oligodendrocyte markers from the whole-hypothalamus homogenates in adult wild type (TRα1++) and TRα1+m mutant, in which TRα1 signaling was reactivated either postnatally or pre- and postnatally by crossing to endogenously hyperthyroid TRβ knockout mice or additionally bred by hyperthyroid TRβ knockout dams. Raw western blots (A-C) and their quantifications (B,D) for the expression of classic oligodendrocyte markers (A,B) and some of the differentially expressed genes between TRα1++ and TRα1+m (C,D) on the TRβ knockout background (postnatal rescue) and additionally bred by TRβ knockout dams (pre- and postnatal rescue). *P<0.05 for TRα1++ versus TRα1+m mutant; ^#^*P*<0.05 for additional effect of prenatal treatment (two-way ANOVA, *n*=3 per group, Table S1). Data are mean±s.d. AU, arbitrary unit.

Based on these observations, we hypothesized that impaired TRα1 signaling during an earlier developmental period would be responsible for this irreversible defect, as oligodendrocyte development in mice occurs in several perinatal waves. To investigate this, we aimed to reactivate TRα1 signaling before and after birth using our established approach of backcrossing the TRα1+m mice to the TRβ knockout strain (Forrest et al., 1996). The TRα1+m TRβ^−/−^ double-knockout mice are endogenously hyperthyroid in postnatal life, as removal of TRβ results in an impaired feedback loop within the hypothalamus–pituitary–thyroid axis and thus elevated thyroid-stimulating hormone (TSH) levels. This triggers high release of T3 and T4 from the thyroid gland and reactivates the mutant TRα1 (Tinnikov et al., 2002). Therefore, any reversal of a TRα1+m phenotype on the TRβ^−/−^ background confirms that it occurs postnatally and is furthermore independent of TRβ signaling, as this receptor has been removed in the double mutants. As a second group, we included TRα1+m TRβ^−/−^ double knockouts that were born to TRβ^−/−^ knockout mothers, which are hyperthyroid throughout pregnancy and in which the mutant TRα1 can be reactivated additionally during embryonic development (Wallis et al., 2008) to test for reversal in this period. The resulting animals were then analyzed as adults (Fig. 5, Table S1). Our data unequivocally show that the postnatal reactivation of TRα1 signaling leads to an unaltered expression of the previously impaired expression of CNP, MOG, PLP1 and CLDN11 when comparing the wild types (TRα1++) and TRα1+m genotypes, whereas the additional reactivation before birth in TRβ^−/−^ dams has little effect. Likewise, DPYD and HCN2 were no longer lower in TRα1+m compared with the respective controls. Interestingly, TMEM117, which was not affected by the TRα1 mutation at the protein level, was lower in both groups that were exposed to elevated thyroid hormone prenatally.

Taken together, these data suggest that early postnatal thyroid hormone signaling via TRα1 is required for proper hypothalamic oligodendrocyte development, as reflected in impaired marker gene expression of CNP, MOG, PLP1 and CLDN11, whereas prenatal and adult thyroid hormone action has little effect on their architecture.

## DISCUSSION

Single-cell RNA sequencing has been gaining in popularity as a means to investigate cell-type composition, gene expression profiles, and cellular development/differentiation in tissues, organs, and even at the whole-animal scale (https://www.humancellatlas.org/) (Cao et al., 2019, 2020; Huang et al., 2022 preprint; Smajić et al., 2022). Indeed, numerous single-cell studies of the hypothalamus have been carried out in recent years, starting with the cataloging of neuronal and non-neuronal cell types and the identification of gene expression dynamics in these cellular populations (Campbell et al., 2017; Chen et al., 2017; Romanov et al., 2017). Here, we set out to use single-cell transcriptome profiling to study the role of TRα1 in hypothalamus development and cell-type diversity in the mouse. We show that the defective TRα1 signaling has surprisingly little effect on the development of hypothalamic neuronal populations, but it heavily affects hypothalamic oligodendrocytes.

Comparing our results to published datasets of the hypothalamus (Hajdarovic et al., 2022; Campbell et al., 2017; Chen et al., 2017; Kim et al., 2020; Moffit et al., 2018; Mickelson et al., 2019) revealed similarities as well as differences. The major cell types, including neurons, astrocytes, oligodendrocytes and OPCs were common across the datasets, expressing similar differentially expressed genes (Fig. S15). However, the composition of these cell types varied dramatically between the datasets. For example, neurons were nearly twice as abundant in our dataset compared with that of Kim et al. (2020). Moreover, many of the rarer cell populations, including mural cells and ependymocytes, were observed only in a few datasets, and were absent in ours, at least at this clustering resolution. This is most likely due to nuclei versus cell sequencing as well as usage of distinct tissue dissociation methods (Denisenko et al., 2020; Sreenivasan et al., 2022). Indeed, these factors represent inherent limitations of the single-cell sequencing technologies, particularly when comparing across datasets. That said, our dataset most resembled that of Hajdarovic et al. (2022) in terms of the cellular composition. Moreover, similar to theirs, and unlike, for example, Chen et al. (2017; hypothalamus) and La Manno et al. (2018; cortex), we did not observe the intermediate cell states in the oligodendrocyte differentiation, namely the committed oligodendrocyte progenitors and the newly formed oligodendrocytes (Fig. S16A-C). This was further confirmed by integrating our data to the recently published curated hypothalamus single-cell atlas HypoMap (Steuernagel et al., 2022) (Fig. S16D,E).

Given our observation of the 72% reduction of *Pvalb^+^*/*Syt2^+^* hypothalamic neurons in TRα1+m mice, we investigated whether the reduced affinity to thyroid hormone results in alteration of gene expression or cellular composition across other neuronal cells. Importantly, our analysis identified that the majority of known hypothalamic neuronal populations, e.g. POMC, orexin (hypocretin), etc., were not strongly affected by the impaired TRα1 signaling, in terms of the number of cells as well as their transcriptome, except for a handful of genes, including *Pisd*. This finding was confirmed by independent histological techniques (Fig. S7) as well as differential gene expression analyses (Fig. S10). It is possible that the altered thyroid signaling did affect other smaller neuronal populations that we could not detect either because of our unbiased analysis approach or because we were only able to probe a fraction of the cells in the hypothalamus, resulting in insensitivity to rare cell types. The use of higher throughput technologies, such as single-cell combinatorial indexing RNA sequencing (sci-RNA-seq) could be used to address the latter, albeit at the expense of reduced gene depth and increased cost. Methods such as the ‘local cellular heuristic neighbourhood enrichment specificity score’ (lochNESS) scoring system that are being currently developed could also improve unbiased analysis strategies (Huang et al., 2022 preprint).

### Oligodendrocytes as a major target of developmental thyroid hormone action

Given that thyroid hormone has been shown to be important for hypothalamic parvalbumin neuron development (Mittag et al., 2013), we were surprised that the vast majority of other hypothalamic neurons were not obviously affected in TRα1+m mice. The massive defect in oligodendrocyte development, by contrast, was not too unexpected given the well-established role of thyroid hormone in this context in other brain regions, as evidenced by severe white matter abnormalities in children with untreated congenital hypothyroidism (Perri et al., 2021) or individuals with Allan–Herndon–Dudley syndrome, a disorder characterized by severely impaired thyroid hormone import into the brain (Valcárcel-Hernández et al., 2022). Similar observations have been made in other species, such as zebrafish (Farías-Serratos et al., 2021) or rats (Barradas et al., 2001). These previous studies indicated that oligodendrogenesis was not entirely abolished by reduced thyroid hormone signaling, but instead only delayed with respect to some genes such as *Cnp* or *Mbp*, which were normalized in the adult stage, whereas some markers remained permanently affected, including PLP1 (Barradas et al., 2001). Similarly, in our study we found normal protein expression of SOX10 and OLIG2 as early markers and MBP and ARSG as markers for mature oligodendrocytes, whereas other protein markers, such as PLP1, CNP, MOG and CLDN11, were still lower in TRα1+m mice. Remarkably, our study identified several other genes that were disrupted in this cell type at the transcript level, and some of these, including the genes encoding the sodium/potassium channel HCN2 and the dihydropyrimidine dehydrogenase DPYD, could be confirmed at the protein level. Although our findings support the notion that mRNA and protein level do not necessarily match in oligodendrocytes, as expected from the heavily regulated translation in this cell type (Xu et al., 2020; Laursen et al., 2011; Müller et al., 2013; Martin et al., 2022; Bauer et al., 2012), both technologies confirm a generally altered appearance of mature oligodendrocytes in TRα1+m mice. In agreement with observations in children with congenital hypothyroidism (Gupta et al., 1995), these findings suggest that thyroid hormone may be less important for the start of the myelination process, but very relevant for normal maturation, as evidenced by the number of differentially expressed genes and the clearly distinct UMAP embeddings between the oligodendrocytes from wild-type and TRα1+m mice.

### Timing of TRα1 action in oligodendrocyte development

Our observation that oligodendrocyte development can be rescued by early postnatal restoration of TRα1 signaling fits well with findings in humans; for example, a 14-month-old girl with untreated congenital hypothyroidism still showed rescue of myelination after thyroid hormone substitution (Alves et al., 1989). Again, similar to our data, when children with congenital hypothyroidism picked up early by routine neonatal screening were analyzed by MRI before thyroid hormone substitution, no difference was observed compared with unaffected children (Siragusa et al., 1997), suggesting that perinatal thyroid hormone signaling is of negligible relevance. The importance of the postnatal period was further supported by recent studies in a mouse model of Allan–Herndon–Dudley syndrome, in which the lower PLP1 expression as a marker for mature oligodendrocytes could be rescued when thyroid hormone import into the brain was restored postnatally using an AAV-based gene therapy approach (Sundaram et al., 2022). Very elegant studies using a conditional TRα1 mutant revealed that, at least in the cerebellum, the receptor has two different roles during development (Picou et al., 2012). Initially, it shapes the environment for oligodendrocyte progenitor cells through actions in other cell types, whereas at later stages it acts cell-autonomously to arrest differentiation and drive maturation (Picou et al., 2012). Given that oligodendrocyte generation in mice is known to peak at 2-4 weeks postnatally, with only minor production in adult mice (Bergles and Richardson, 2015; Kuhn et al., 2019), and that the terminal differentiation of oligodendrocytes also occurs postnatally (Li et al., 2009), we conclude that thyroid hormone-mediated TRα1 signaling is crucial in this critical time period. However, the window seems to close at some point, given that oligodendrocyte marker expression could not be rescued in adulthood. The precise duration of this crucial period remains to be fully established, keeping in mind that mice are born at a somewhat earlier developmental stage compared with humans.

## Conclusions

Our findings of the importance of thyroid hormone for postnatal oligodendrocyte development clearly underline the necessity to identify affected individuals, e.g. those with congenital hypothyroidism, early after birth for immediate treatment to avoid irreversible brain damage. Most importantly, unlike several neuronal cell types, which are specific to the hypothalamus, these non-neuronal cells are found throughout the brain (Chen et al., 2017), suggesting that other neuroanatomical areas might be similarly affected. Given the growing resource of genome-wide studies of T3 target genes in different tissues (Zekri et al., 2022), our study provides a unique foundation to dissect the developmental from the acute actions of the hormone in brain development.

## MATERIALS AND METHODS

### Experimental animals

Animals were housed in groups at 22±1°C at constant 12 h light/dark cycle with *ad libitum* access to food and water. Experiments were conducted in adult male mice. TRα1R384C heterozygous mutant mice (TRα1+m) as well as the combinations and breedings with endogenously hyperthyroid TRβ knockout mice have been described previously (Harder et al., 2018; Tinnikov et al., 2002). All mice were bred on the C57/BL6NCr background at the Gemeinsame Tierhaltung of the University of Lübeck. Hyperthyroidism in adult animals was induced by treatment with 0.5 mg/l 3,3′,5-triiodo-L-thyronine (T6397, Sigma-Aldrich) in 0.01% bovine serum albumin (BSA) and tap water for 12 days (Johann et al., 2019). For single-cell sequencing, a total of six wild-type and six TRα1+m mice were used across two independent experimental repeats. Immediately after sacrificing the animal by CO_2_ inhalation and cervical dislocation, the whole brain was quickly collected and placed on absorbent paper in a Petri dish. Then, the hypothalamus was dissected, frozen with liquid nitrogen and then stored at −80°C until the samples were further processed. All animal procedures were approved by the MELUND Schleswig-Holstein (Kiel, Germany) or Djurförsöksetiska Nämnd (Stockholm, Sweden), and the respective power calculations for determination of group sizes are part of the ethical application.

### Nuclei isolation, 10x library preparation, and sequencing

Nuclei were isolated from snap-frozen hypothalamus tissue with an adapted version of the published sci-RNA-seq3 protocol for nuclei isolation (Cao et al., 2019). The samples were cut into small pieces in 0.5 ml ice-cold cell lysis buffer (CLB) [10 mM Tris-HCl, pH 7.4, 10 mM NaCl, 3 mM MgCl_2_, 1%SUPERase In RNase Inhibitor (20 U/μl, Ambion), 2% BSA and 0.1% IGEPAL CA-630], then another 0.5 ml CLB was added and the tissue was further singled out by resuspension with a 1 ml pipette. The samples were strained using a 40 µm cell strainer and pooled by genotype (wild type or TRα1+m) into respective Eppendorf LoBind tubes. The nuclei were pelleted (4°C, 500 ***g***, 5 min) and resuspended in 1 ml nuclei suspension buffer (NSB) [10 mM Tris-HCl, pH 7.4, 10 mM NaCl, 3 mM MgCl_2_, 1%SUPERase In RNase Inhibitor (20 U/μl, Ambion) and 2% BSA]. The nuclei were checked for successful lysis under a microscope using a cell counter. After a second cell strainer step into a new LoBind tube, the nuclei were again pelleted, resuspended in 1 ml NSB, pooled (across the three biological samples within a genotype), and counted using a cell counter. In a new LoBind tube, the nuclei were diluted into 1000 nuclei/μl in 20 μl and directly processed for single-cell RNA sequencing using the 10x Genomics protocol (V3.1). The quality of the cDNA was assessed using TapeStation (HighSensitivity D5000 Screen Tape), as recommended by the 10x protocol. Sequencing was performed at the Max Planck Institute Sequencing facility with asynchronous paired end sequencing NovaSeq 6000 (Illumina). The experiments were performed in two independent repeats, in each of which cells from three mice per genotype were pooled.

### Processing of sequencing reads and filtering

Single-cell feature-barcode matrices were generated from fastq files with the count (cellranger-5.0.1) pipeline using the Mouse reference transcriptome mm10-2020-A (10x Genomics). The include-introns argument was added to count reads falling in the intronic regions, because of single-nuclei sequencing, which are enriched in intronic reads. Across the four sequencing runs, the output resulted in 68,190 barcodes being classified as nuclei. Further custom filtering was performed using Seurat-4.0.5 (Hao et al., 2021), resulting in 56,386 nuclei with a minimum of 900 counts and 700 distinct features (transcripts), a maximum of 5% mitochondrial reads, as well as a doublet score (scrublet-0.2.3; Wolock et al., 2019) below 0.18.

### Normalization, merging, cell clustering and annotation

All of the following steps were carried out using Seurat-4.0.5. To identify the major cell types comprising the murine hypothalamus, we merged the four single-nuclei datasets (two genotypes×two repeats) after log-normalizing the data using the NormalizeData and Merge functions. For cell clustering, first the top 4000 highly variable genes were identified with FindVariableFeatures function using the vst method. The expression data corresponding to these features was then centered and scaled using the ScaleData function and used to carry out principal component (PC) analysis. The top 20 PCs were used to calculate a neighbor graph (FindNeighbors), based on which Louvain clustering was performed with a resolution of 0.008 (FindClusters). To assign cell-type identities to the identified clusters, we determined the differentially expressed features using the FindAllMarkers function using the following parameters min.cells.group=50, min.pct=0.1, and logfc_threshold=0.25. The differentially expressed features were ranked based on the Wilcoxon Rank Sum test, for each of the identified clusters. The clusters were annotated manually based on the positively differentially expressed features. The top 20 PCs were embedded onto two dimensions based on the UMAP algorithm using the RunUMAP function.

### Batch correction across experimental repeats of individual clusters

All of the following steps were carried out using Seurat-4.0.5, harmony-0.1.0, as well as standard R packages. To visualize individual clusters as well as for further sub-clustering, the data in the PC space corresponding to each cluster was batch corrected across the two experimental repeats by using RunHarmony, with 20 PCs when the cluster contained greater than 1000 cells, otherwise with ten PCs.

### Differential gene expression analysis across genotypes

All of the following steps were carried out using Seurat-4.0.5 as well as standard R packages. We determined the differentially expressed features between wild-type and TRα1+m-expressing cells across all the annotated cell clusters or sub-clusters using the FindAllMarkers function using the following parameters: min.cells.group=0, min.pct=0.1, and logfc_threshold=0.5. The obtained gene list was further filtered for statistical significance based on the Wilcoxon Rank Sum test using Bonferroni-adjusted *P*-values<1E−20. This stringent cut off was chosen to reduce false positives.

### Integration of oligodendrocytes and OPCs with the HypoMap dataset

All of the following steps were carried out using Seurat-4.0.5 as well as standard R packages. The HypoMap dataset was downloaded as a Seurat object in RDS format from https://doi.org/10.17863/CAM.87955. Harmony-based integration was performed as before, with 20 PCs. For integration, the group.by.vars was set up such that the integration aligned the data corresponding to individual studies in the HypoMap dataset as well as the two experimental repeats (separately) in our dataset.

### *In situ* hybridization and immunohistochemistry

*In situ* hybridization histochemistry and immunohistochemistry were performed on 20-µm-thick brain sections as described previously (Mittag et al., 2009, 2013). The primary antibodies were anti-orexin (sc8070, Santa Cruz Biotechnology, 1:4000), anti-NeuN (24307T, Cell Signaling Technology, 1:1000), anti-GFAP (Z0334, Dako, 1:500) or anti-parvalbumin (pv27, Swant, 1:2000) with a biotinylated secondary antibody (BA-1000 Vector Laboratories, 1:250).

### Western blotting

Western blotting was performed as described previously (Oelkrug et al., 2020). Briefly, 20 µg total hypothalamic protein were separated on a 12% SDS polyacrylamide gel (TGX Stain FreeTM FastCastTM Acrylamide Kit, Bio-Rad Laboratories) and transferred onto a polyvinylidene fluoride membrane (IPVH00010, Merck Millipore). Membranes were probed with the mature oligodendrocyte marker panel; OLIG2, MBP, MOG, SOX10 and CLDN11 (ab109186, ab218011, ab109746, ab155279/ab227680 and ab53041, respectively; abcam), PLP1, CNP and MBP (mAb#28702, mAb#5664 and mAb#78896, respectively; Cell Signaling Technology), ARSG (AF4600, R&D Systems), TMEM117, SLCO3A1 and DPYP (#PA5-61189, #PA5-42457 and #PA-22302, respectively; Thermo Fisher Scientific), TMEM117 (SAB2102450, Sigma-Aldrich), HCN2 (APC-030, Alomone Labs), SYP, GFAP or TUBB3 (MA5-14532, MA5-12023 or PA5-85639, respectively; Thermo Fisher Scientific) as primary antibodies (all diluted 1:1000) followed by a peroxidase-conjugated secondary antibody (goat anti-rabbit-IgG HRP-conjugated at 1:5000 or goat anti-mouse-IgG HRP-conjugated at 1:5000; both from Dako). References for these primary antibodies can be found on the provider's webpages (see Fig. S17 for example reference blots). The antigens were visualized using Clarity MaxTM Western ECL Substrate (Bio-Rad Laboratories) and an ECL Plus western blotting detection system (ChemiDocTM, Touch Imaging System, Bio-Rad Laboratories). Band intensity was quantified using Image Lab software 6.1 (Bio-Rad Laboratories) and data were normalized to total protein load. In cases for which more than one antibody is mentioned for a specific antigen, the results of both were comparable. Data were analyzed using Prism8 (GraphPad) with a two-way ANOVA and significance for either factor or interaction is indicated in the figures. As the results from Figs 4 and 5 were obtained from separate gel runs, there is no direct comparison between the respective groups to avoid technical bias.

## Supplementary Material

10.1242/develop.201228_sup1 Supplementary information
