# Supplementary material for: Single-cell RNA-based phenotyping reveals a pivotal role of thyroid hormone receptor alpha for hypothalamic development
Source: Development. 2023 Jan 30;150(3):dev201228. doi: 10.1242/dev.201228 (PMC10110490; doi:10.1242/dev.201228)
Supplement: Supplementary information [file develop-150-201228-s1.pdf]

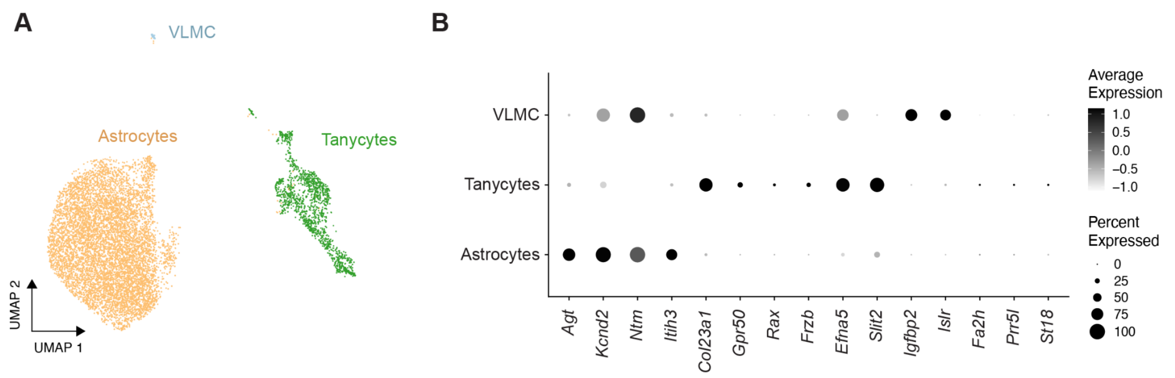

Fig. S1. Sub-clustering of the main astrocyte cluster reveals the presence of tanycytes and VLMCs. A. UMAP embeddings of the main astrocyte cluster after harmony-based batch-correction (materials and methods). **B.** Differentially expressed genes, based on which the three sub-clusters were annotated.

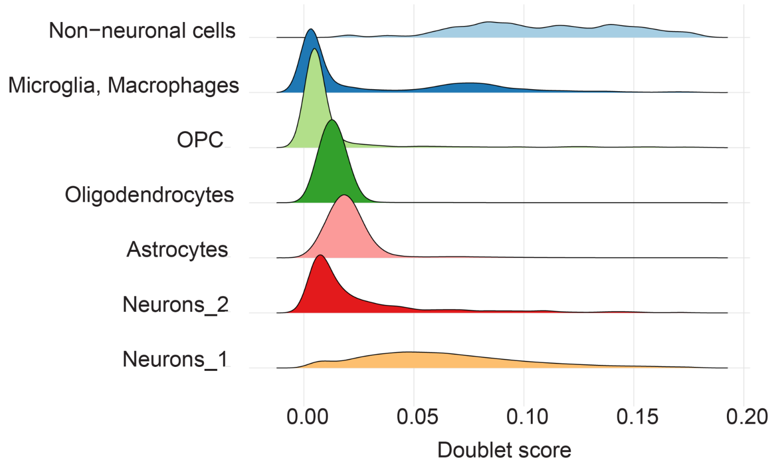

Fig. S2. Histogram of doublet scores, segregated by clusters (cell types). Cells with doublet score > 0.18 were filtered out.

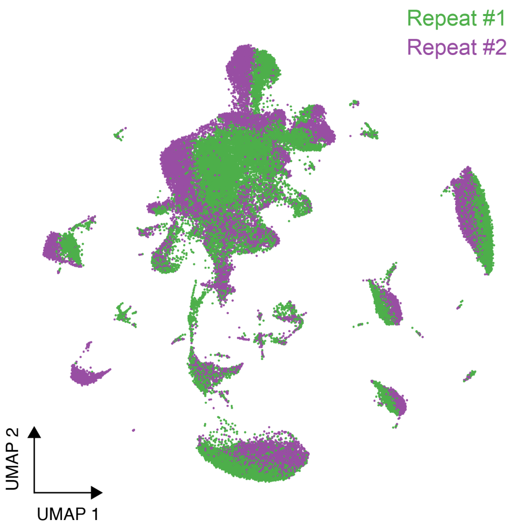

Fig. S3. Batch effects are present in the data. UMAP embeddings of the entire dataset without batch-correction, where cells are colored by experimental repeat.

Development • Supplementary information

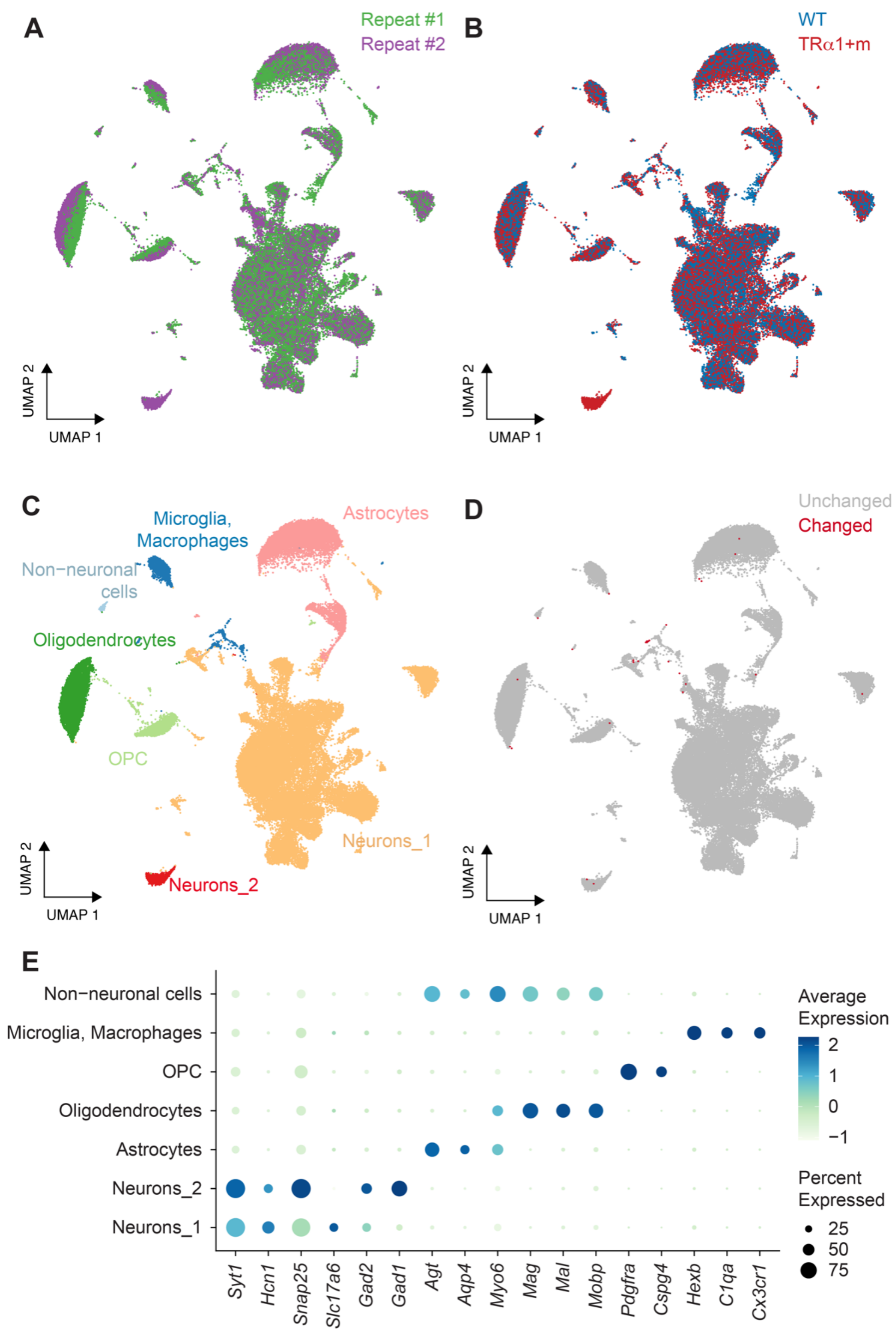

Fig. S4. Harmony-based batch-correction at the entire dataset level does not affect clustering or cell type annotation. A-C. UMAP embeddings of the entire dataset after harmony-based batch-correction, where cells are colored by **A**. experimental repeat, **B**. genotype, or **C**. cell type. **D**. The cell type annotation of 30 cells (red) changed as a result of harmony-based batch-correction. **E.** The differentially expressed genes between the clusters also remained unchanged in comparison to **Fig. 1C.**

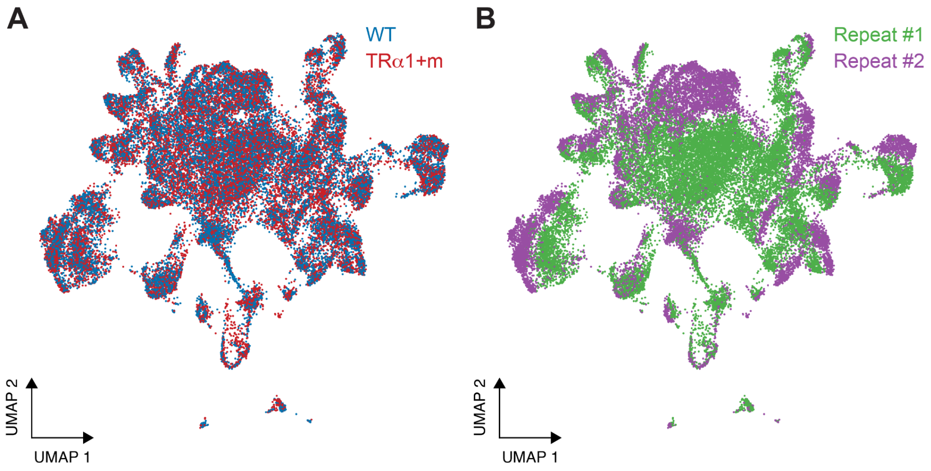

Fig. S5. Batch effects are also apparent in the data at the cluster level. UMAP embeddings of the neuronal cluster without batch-correction, where cells are colored by **A.** genotype or **B.** experimental repeat.

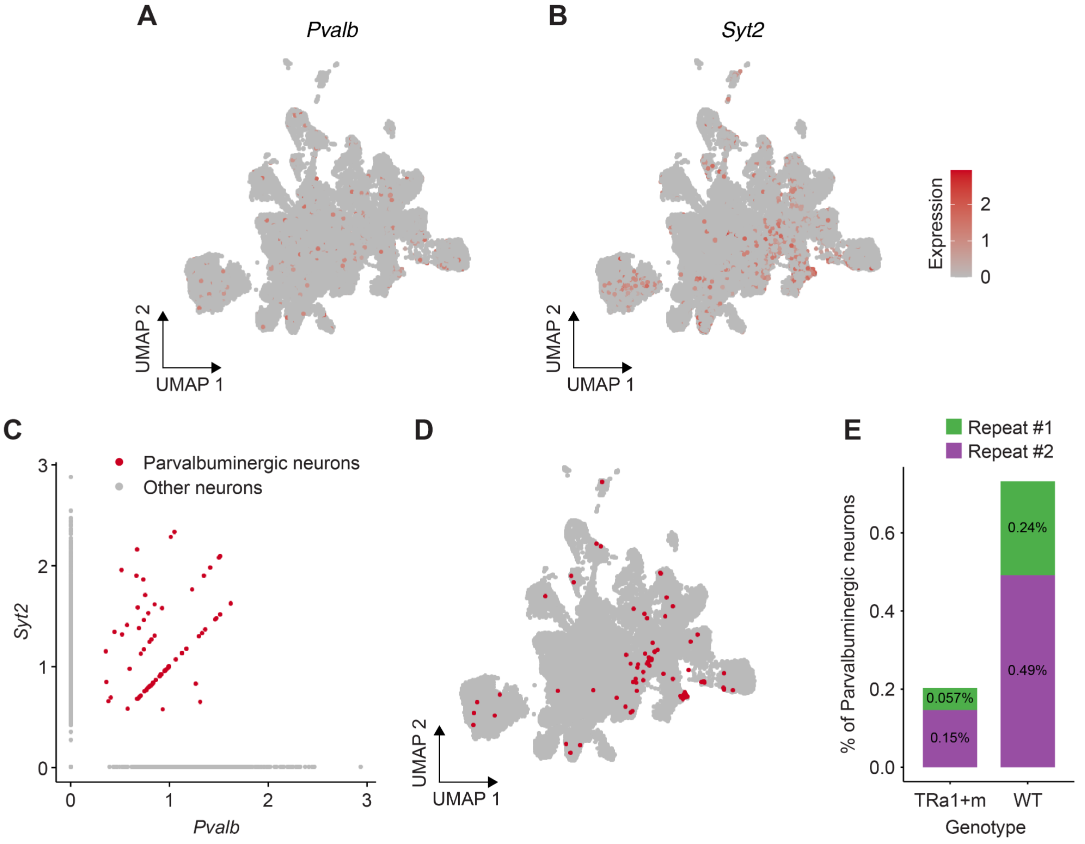

Fig. S6. Cellular composition of parvalbuminergic neurons. A-B. UMAP embedding of the Neurons_1 cluster highlighting the expression of the marker genes of parvalbuminergic neurons (*Pvalb*, *Syt2*) **C.** *Pvalb*+/*Syt2*+ neurons are defined as parvalbuminergic neurons. **D**. UMAP embedding of the Neurons_1 cluster highlighting the parvalbuminergic neurons (red). **C**. The cellular composition of parvalbuminergic neurons as a percentage of the total number of neurons in each of the four datasets (to account for varying cell numbers between the datasets).

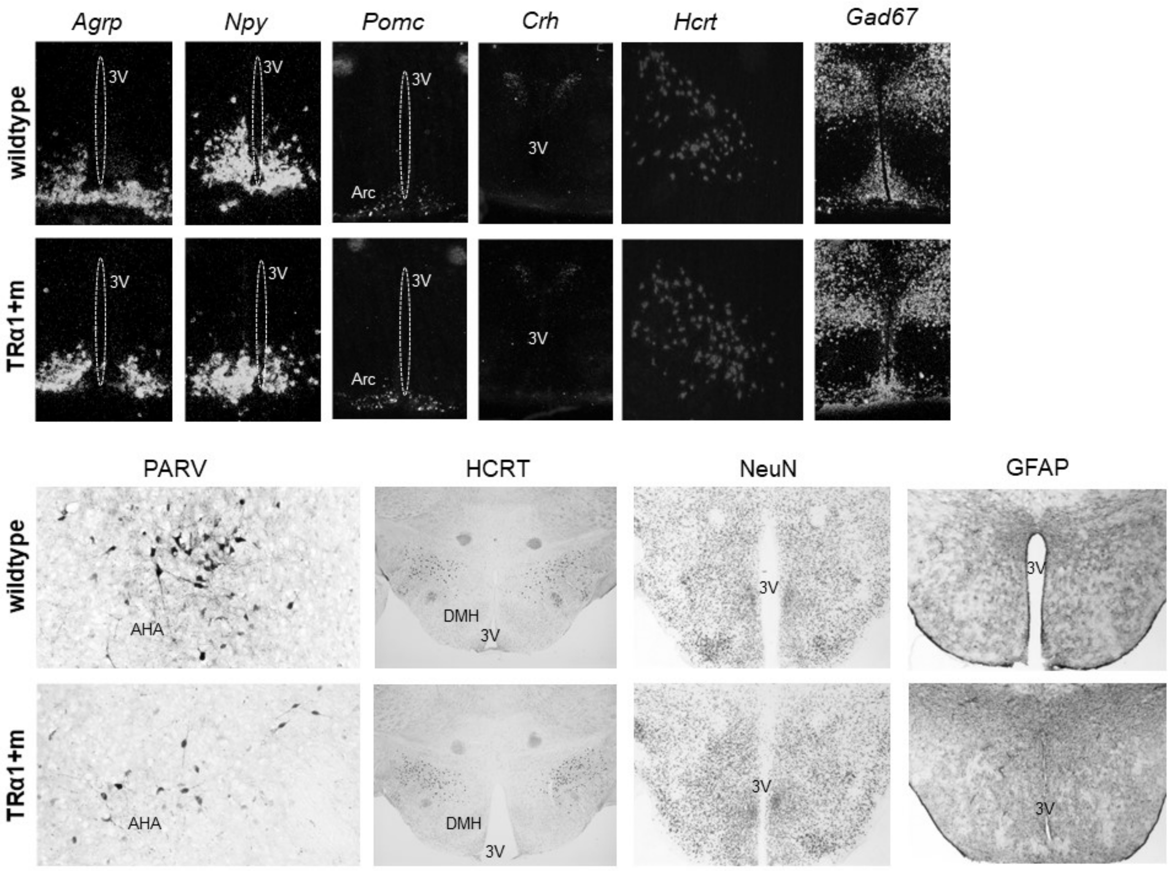

Fig. S7. Expression of marker genes. (**Top**) In situ hybridisation for agouti-related peptide (*Agrp*), neuropeptide Y (*Npy*), proopiomelanocortin (*Pomc*), corticotropin-releasing hormone (*Crh*), Orexin (*Hcrt*), and glutamate decarboxylase 67 (*Gad67*) as well as (**Bottom**) immunohistochemistry (IH) for HCRT, neuronal nuclei (NeuN) and glial fibrillary acidic protein (GFAP) reveals similar expressions of classic hypothalamic markers between wildtype and TRα1+m mutants, with the expected exception of parvalbumin (PARV). 3V: third ventricle of the hypothalamus, Arc: arcuate nucleus, DMH: dorsomedial hypothalamus.

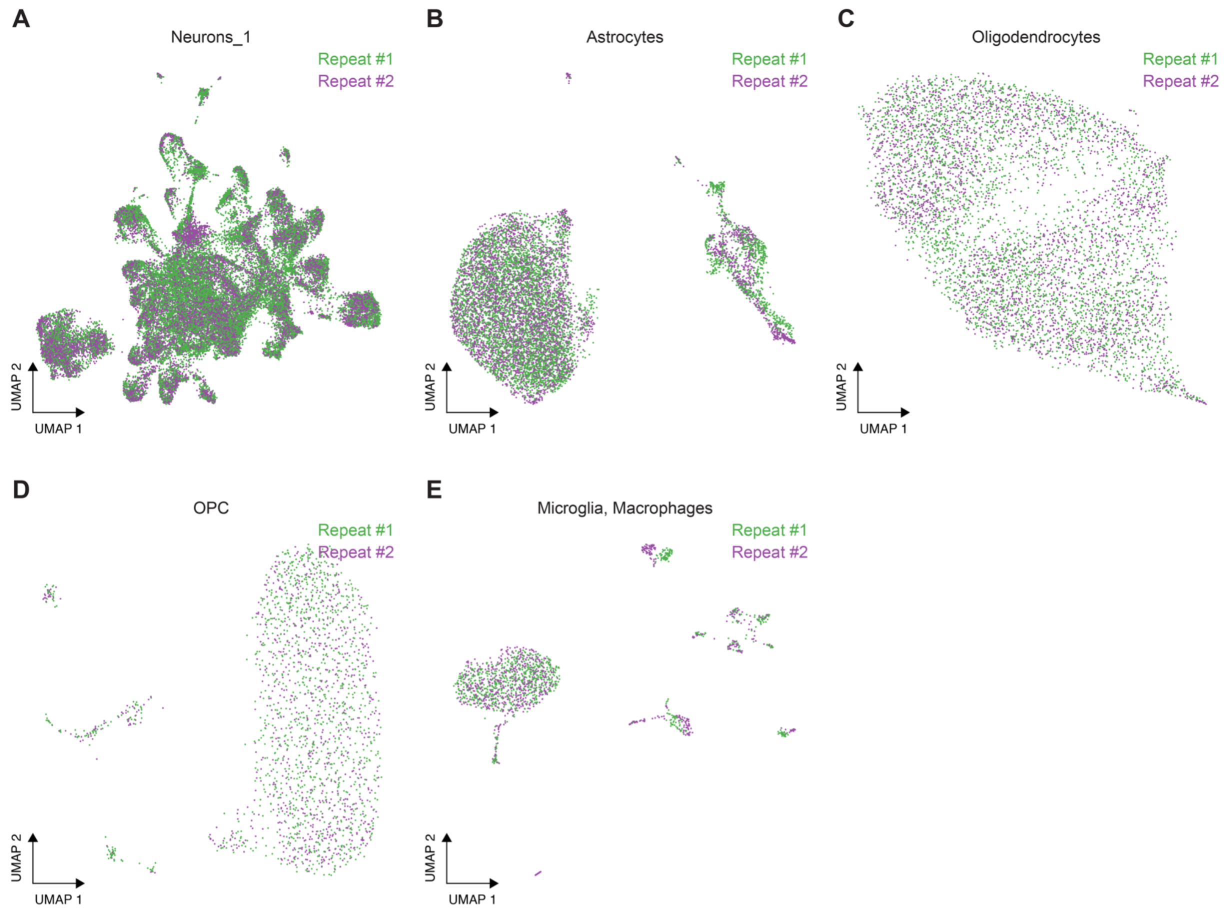

Fig. S8. Harmony-based batch-correction of individual clusters. A-E. UMAP embeddings of the clusters after batch-correction, colored by experimental repeats.

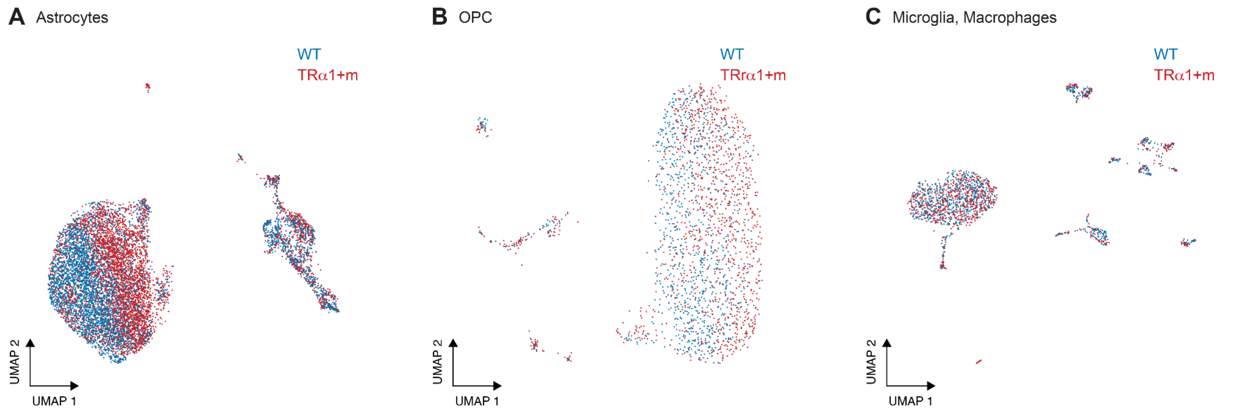

Fig. S9. UMAP embeddings of individual cell types. UMAP embeddings of **A.** Astrocytes **B.** OPC and **C.** Microglia, Macrophages after harmony-based batch-correction across the two experimental repeats. Colored based on the genotype.

Development • Supplementary information

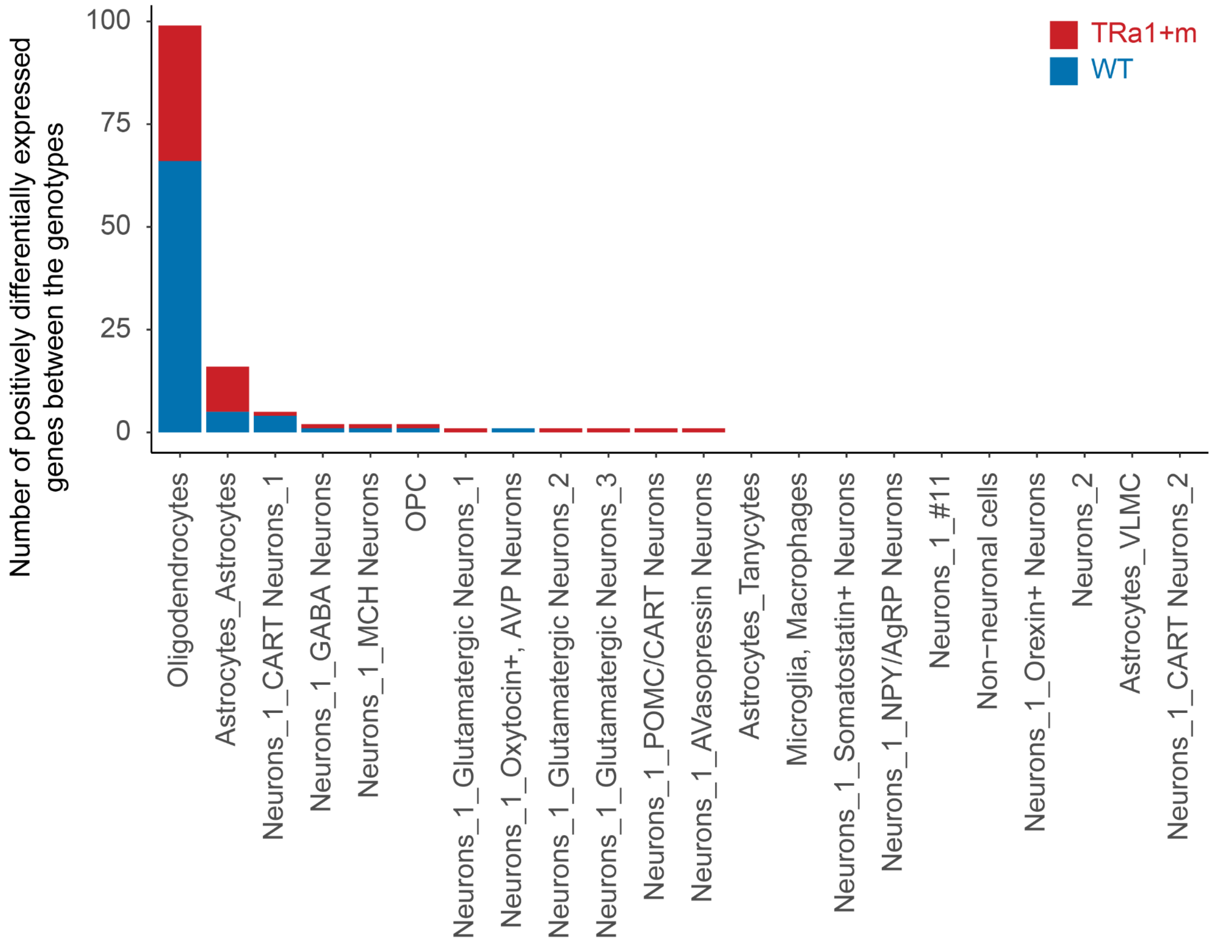

Fig. S10. Number of differentially expressed genes per annotated clusters or sub-clusters. Differential expression analysis was performed between the two genotypes based on the Wilcoxon Rank Sum test for genes expressed in at least 10% of the cells of either of the two genotypes and was further filtered for the absolute average log2 fold change greater than 0.5 and bonferroni adjusted p-values < 1E-20. Positively differentially expressed genes of either of the two genotypes are colored in blue and red for the two genotypes.

Development • Supplementary information

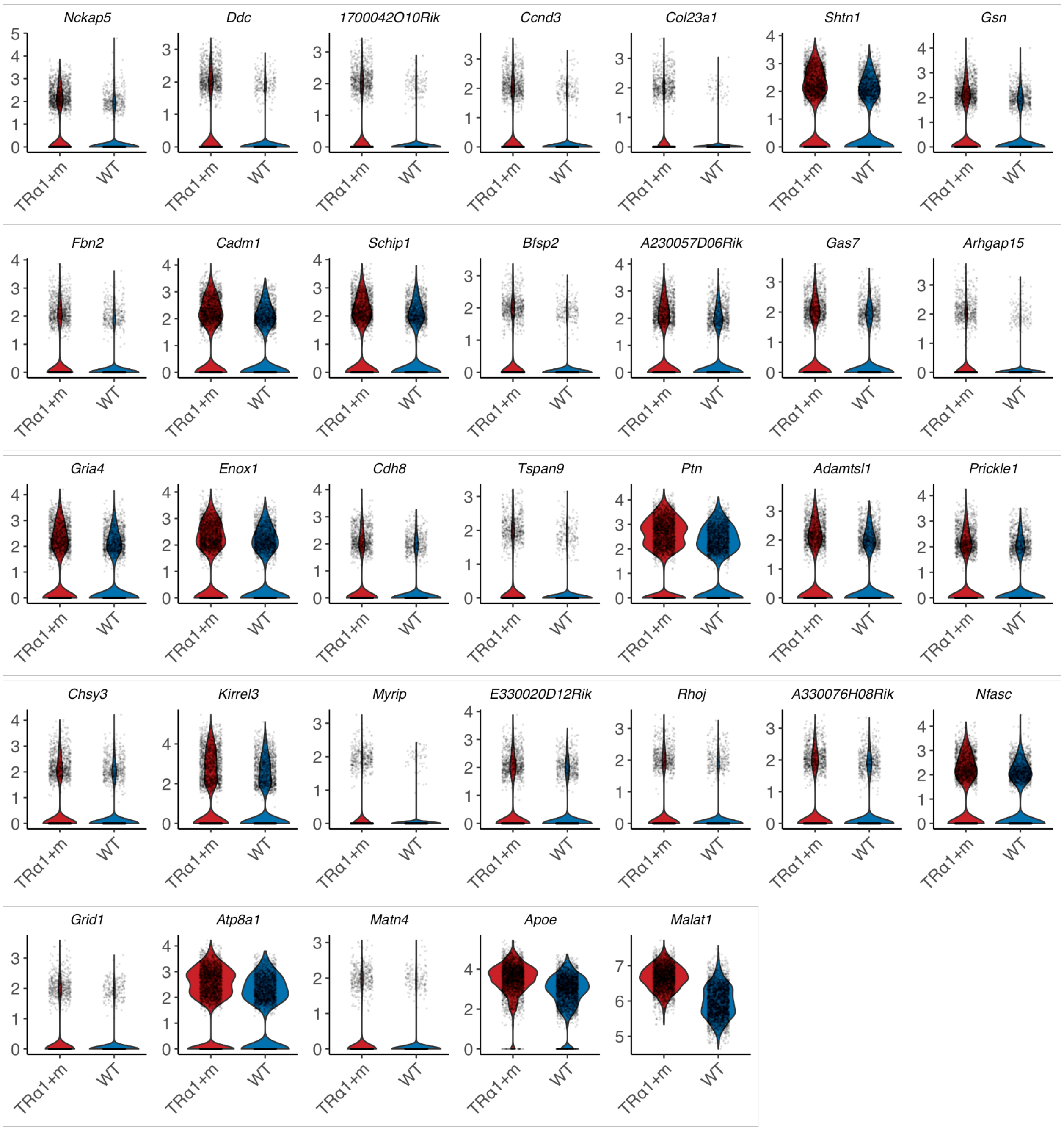

Fig. S11. Expression of differentially expressed genes between the genotypes in oligodendrocytes. Positively differentially expressed genes in the TRα1+m, sorted by decreasing order of the difference in percentage of cells expressing the gene. Statistical information in **Table S2**.

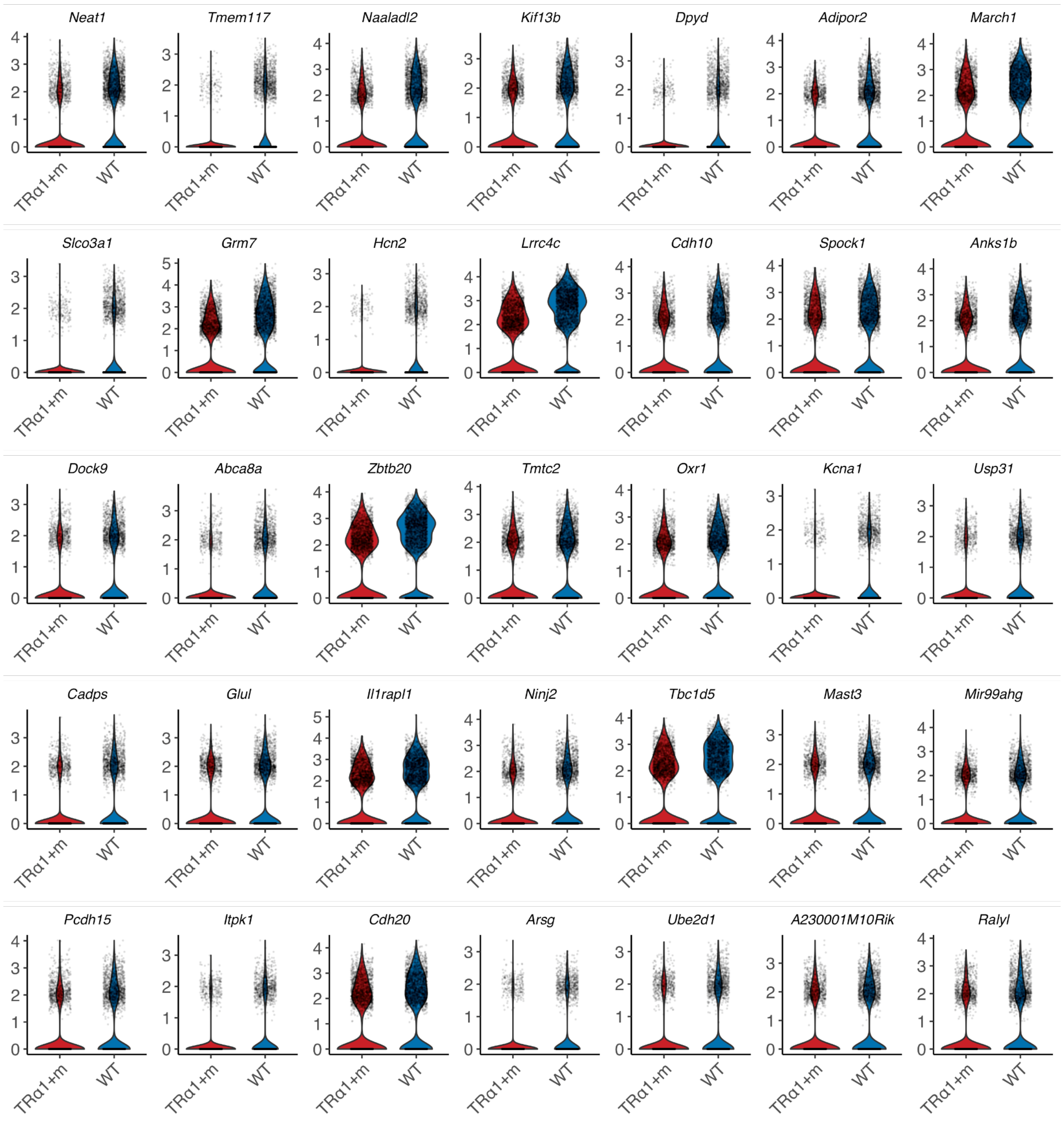

Fig. S12. Expression of differentially expressed genes between the genotypes in oligodendrocytes. Positively differentially expressed genes in the wild type, sorted by decreasing order of the difference in percentage of cells expressing the gene. Continued in **Fig. S13**. Statistical information in **Table S2**.

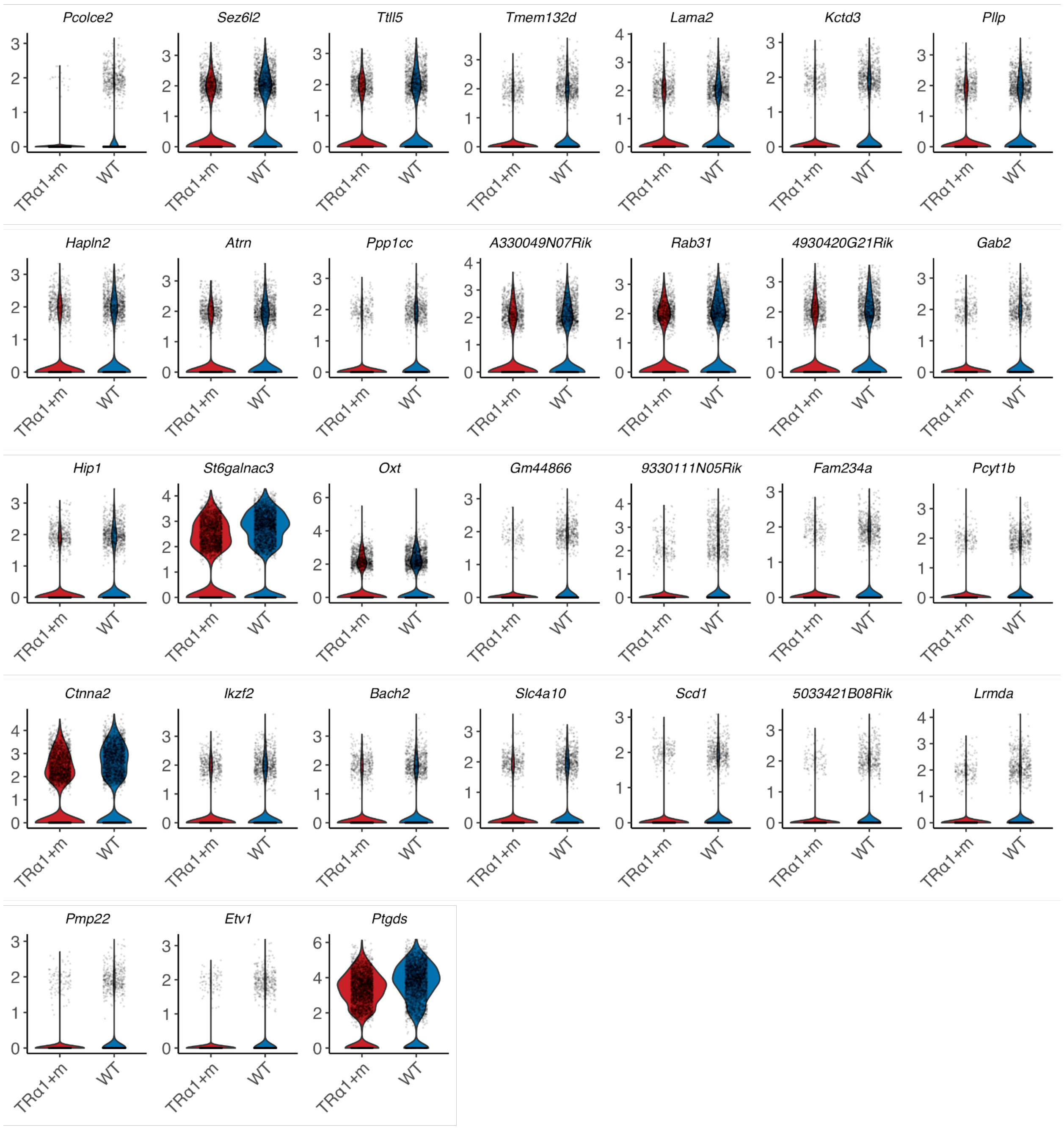

Fig. S13. Expression of differentially expressed genes between the genotypes in oligodendrocytes. Positively differentially expressed genes in the wild type, sorted by decreasing order of the difference in percentage of cells expressing the gene. Continued from **Fig. S12**. Statistical information in **Table S2**.

Development • Supplementary information

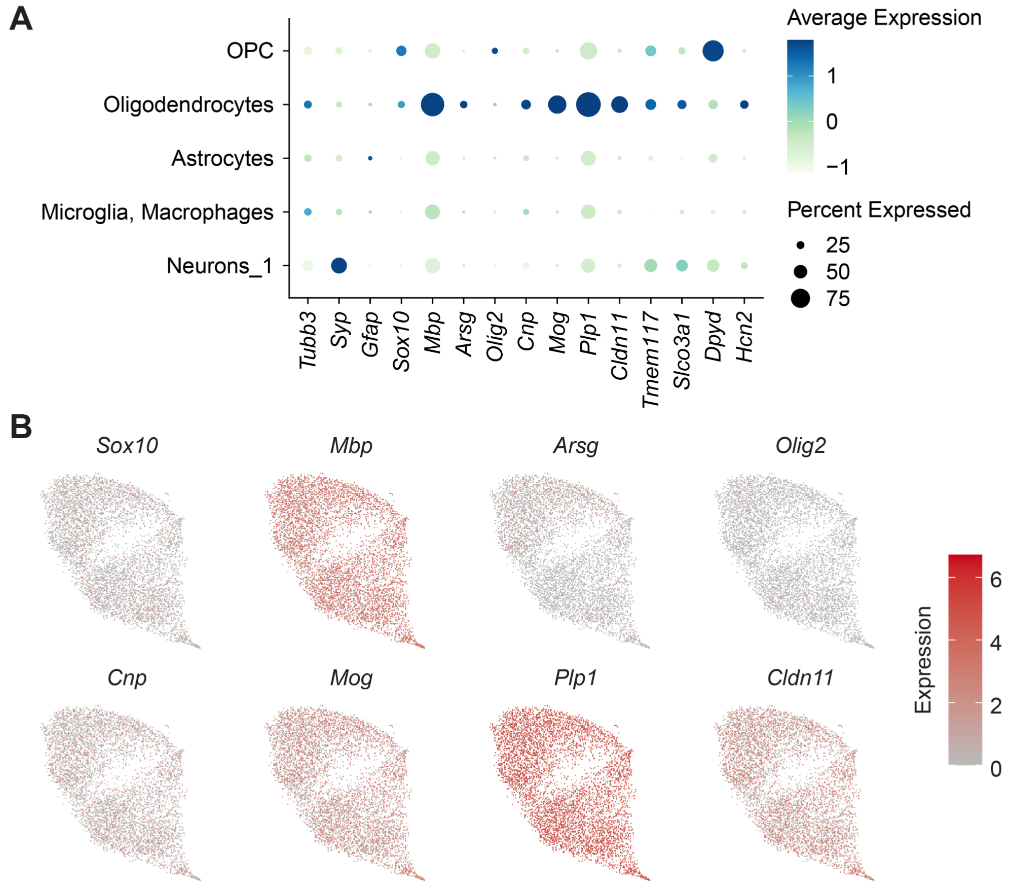

Fig. S14. Gene expression of proteins assayed by western blotting in Figs 4,5. A. Expression of most of the markers are limited to the respective cells. Only cells from the wild type hypothalamus were included, since some of these genes are differentially expressed in the TRα1+m oligodendrocytes. **B**. Expression pattern based on the same UMAP embedding as in **Fig. 3**.

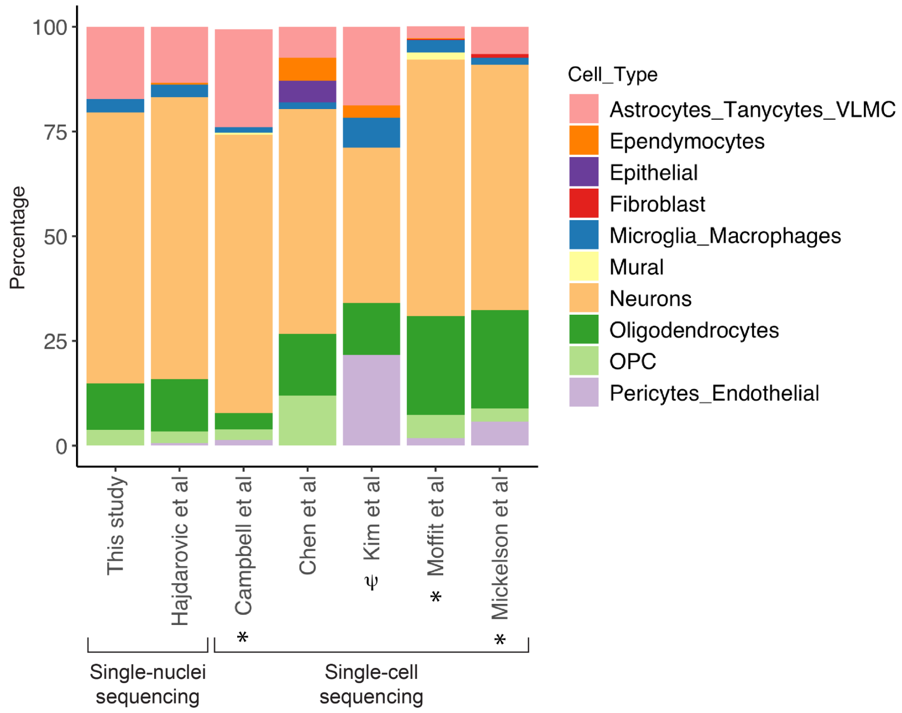

Fig. S15. Comparison of cell type composition between hypothalamus single-nuclei or single-cell RNA sequencing studies (Campbell et al., 2017; Chen et al., 2017; Hajdarovic et al., 2022; Kim et al., 2020; Mickelsen et al., 2019; Moffitt et al., 2018). * Campbell et al, Moffit et al, and Mickelson et al investigated the arcuate hypothalamus/median eminence, rostral hypothalamus, and lateral hypothalamic area, respectively. Ψ Data corresponding to the time point P45 was used from Kim et al. Several cell types (e.g., astrocytes, tanycytes and VLMC) were aggregated to match with the first level clustering results in our dataset. Note: The cell type compositions presented here are based on the original clustering and annotations of the respective studies (i.e. no data integration was performed).

Development • Supplementary information

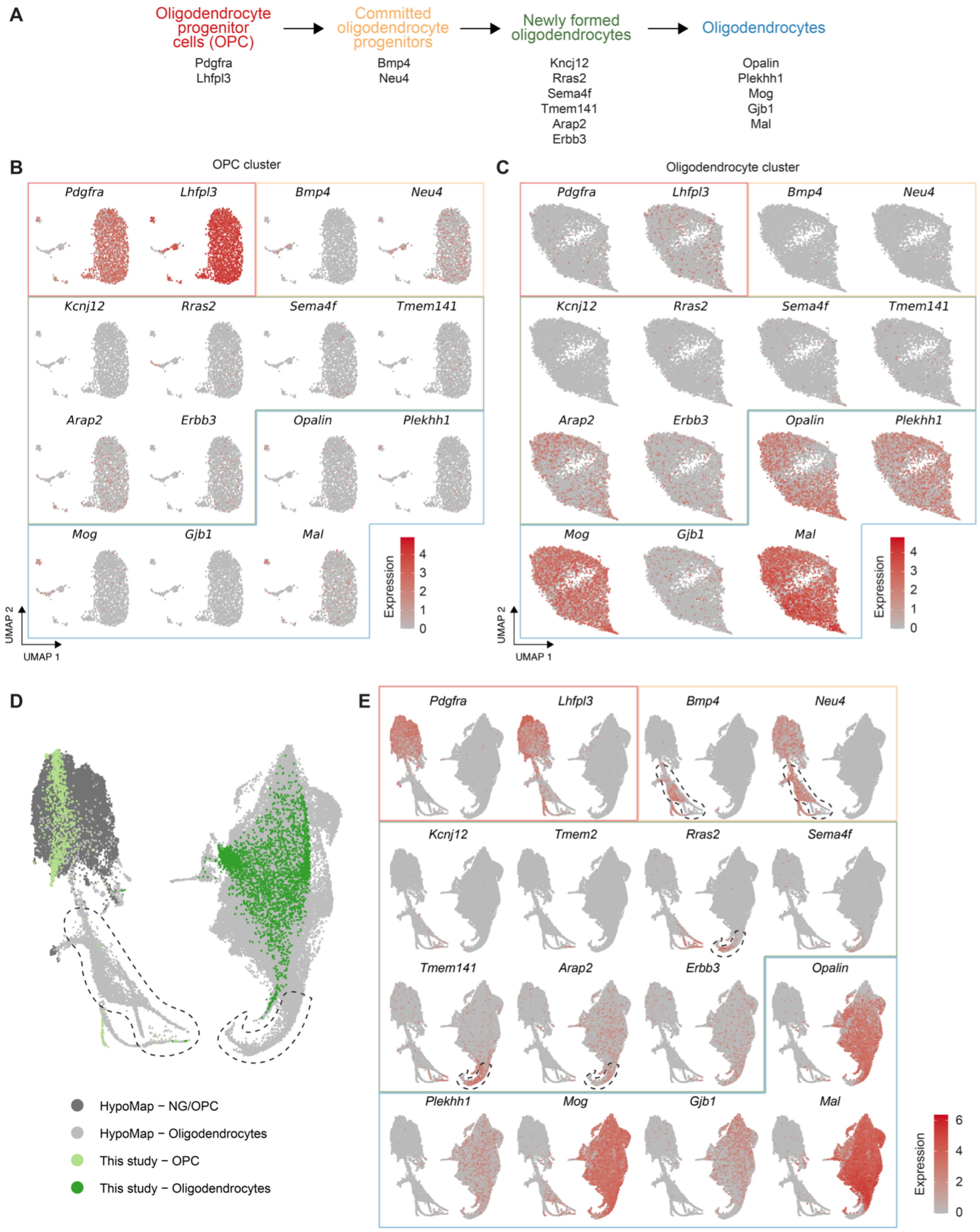
A
Oligodendrocyte progenitor cells (OPC)
Pdgfra
Lhfpl3
Committed oligodendrocyte progenitors
Bmp4
Neu4
Newly formed oligodendrocytes
Kncj12
Rras2
Sema4f
Tmem141
Arap2
Erbb3
Oligodendrocytes
Opalin
Plekhh1
Mog
Gjb1
Mal
B
OPC cluster
C
Oligodendrocyte cluster
Pdgfra Lhfpl3 Bmp4 Neu4
Kcnj12 Rras2 Sema4f Tmem141
Arap2 Erbb3 Opalin Plekhh1
Mog Gjb1 Mal
Expression
4 3 2 1 0
UMAP 2
UMAP 1
D
HypoMap – NG/OPC
HypoMap – Oligodendrocytes
This study – OPC
This study – Oligodendrocytes
E
Pdgfra Lhfpl3 Bmp4 Neu4
Kcnj12 Tmem2 Rras2 Sema4f
Tmem141 Arap2 Erbb3 Opalin
Plekhh1 Mog Gjb1 Mal
Expression
6 4 2 0

Fig. S16. Expression and the lack thereof of marker genes during the differentiation and myelination of oligodendrocytes. A. Four stages of oligodendrocyte formation and the marker genes at each stage (Chen et al., 2017; La Manno et al., 2018). **B,C**. The lack of expression of the known markers of committed oligodendrocyte progenitors (COL) and newly formed oligodendrocytes (NFOL) confirm the lack of these intermediate cells in our dataset. **D**. UMAP embedding of our oligodendrocyte and OPC cells integrated with the curated HypoMap dataset, highlighting the lack of COL and NFOL in our dataset as demarcated by their absence within the dashed black regions, which correspond to the expression patterns of markers shown in **E**. **E**. Expression of the markers in the integrated data.

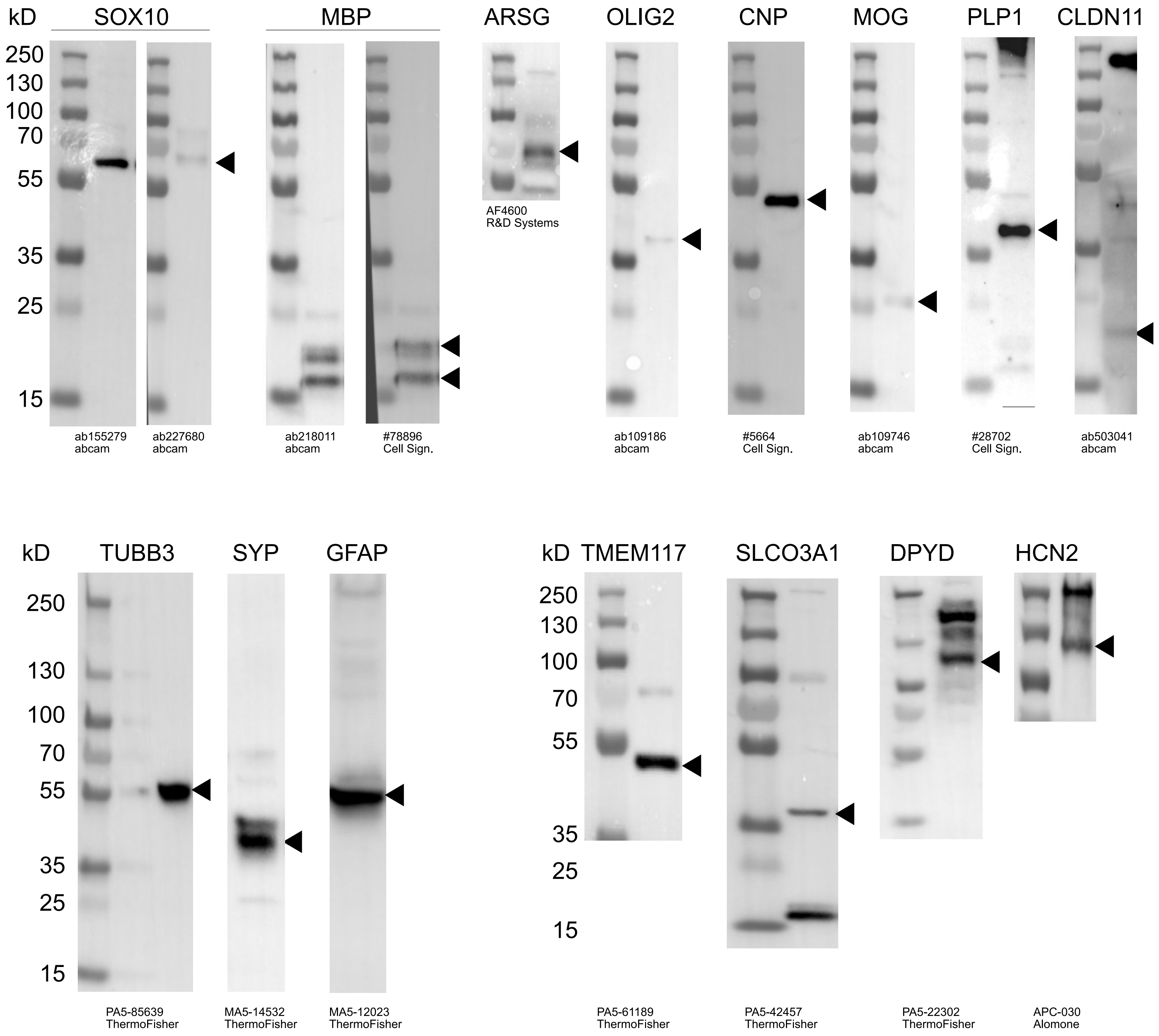

Fig. S17. Size markers for the used antibodies. Western blot images for all antibodies used in the study. Prestained size markers with the indicated molecular weights in kilodalton (kD) were used, and the light-field image of the marker was merged with the ChemiDoc chemiluminescence picture of the detected antibody.

Development • Supplementary information

Table S1. Summary of statistically significant effects of TRα1 signaling in different developmental periods. Asterisks in brackets indicate the significance level of the respective factor in the 2-way ANOVA analysis with *:p<0.05; **:p<0.01; ***:p<0.001

Marker	Affected by TRα1	Effect of adult T3	Rescue by TRβ KO	Rescue by TRβ ko and maternal hyperthyroidism	Conclusion
SOX10	=	=	=	=	Marker not affected
MBP	=	=	=	=	Marker not affected
ARSG	=	=	mildly lower (*)	no additional effect	Marker not affected
OLIG2	=	=	=	=	Marker not affected
CNP	lower (*)	further lowered (*)	normalized	no additional effect	Requires early postnatal TRα1 action
MOG	much lower (***)	no effect	normalized	no additional effect	Requires early postnatal TRα1 action
PLP1	lower (*)	no effect	normalized	no additional effect	Requires early postnatal TRα1 action
CLDN11	lower (**)	no effect	normalized	no additional effect	Requires early postnatal TRα1 action
TMEM117	=	=	mildly lower (*)	further lowered (*)	Marker not affected
SLCO3A1	=	=	=	=	Marker not affected
DPYD	lower (**)	no effect	even higher (*)	no additional effect	Requires early postnatal TRα1 action
HCN2	Lower (*)	further lowered (*)	normalized	no additional effect	Requires early postnatal TRa action

Development • Supplementary information

Table S2. Differentially expressed genes between the two genotypes (TRα1+m versus wild type) across all sub-clusters, without additional filtering.

Click here to download Table S2

REFERENCES

Campbell, J. N., Macosko, E. Z., Fenselau, H., Pers, T. H., Lyubetskaya, A., Tenen, D., Goldman, M., Verstegen, A. M. J., Resch, J. M., McCarroll, S. A., et al. (2017). A molecular census of arcuate hypothalamus and median eminence cell types. *Nat. Neurosci.* **20**, 484–496.

Chen, R., Wu, X., Jiang, L. and Zhang, Y. (2017). Single-Cell RNA-Seq Reveals Hypothalamic Cell Diversity. *Cell Rep.* **18**, 3227–3241.

Hajdarovic, K. H., Yu, D., Hassell, L.-A., Evans, S. A., Packer, S., Neretti, N. and Webb, A. E. (2022). Single-cell analysis of the aging female mouse hypothalamus. *Nat Aging* **2**, 662–678.

Kim, D. W., Washington, P. W., Wang, Z. Q., Lin, S. H., Sun, C., Ismail, B. T., Wang, H., Jiang, L. and Blackshaw, S. (2020). The cellular and molecular landscape of hypothalamic patterning and differentiation from embryonic to late postnatal development. *Nat. Commun.* **11**, 4360.

La Manno, G., Soldatov, R., Zeisel, A., Braun, E., Hochgerner, H., Petukhov, V., Lidschreiber, K., Kastriti, M. E., Lönnerberg, P., Furlan, A., et al. (2018). RNA velocity of single cells. *Nature* **560**, 494–498.

Mickelsen, L. E., Bolisetty, M., Chimileski, B. R., Fujita, A., Beltrami, E. J., Costanzo, J. T., Naparstek, J. R., Robson, P. and Jackson, A. C. (2019). Single-cell transcriptomic analysis of the lateral hypothalamic area reveals molecularly distinct populations of inhibitory and excitatory neurons. *Nat. Neurosci.* **22**, 642–656.

Moffitt, J. R., Bambah-Mukku, D., Eichhorn, S. W., Vaughn, E., Shekhar, K., Perez, J. D., Rubinstein, N. D., Hao, J., Regev, A., Dulac, C., et al. (2018). Molecular, spatial, and functional single-cell profiling of the hypothalamic preoptic region. *Science* **362**,.

Development • Supplementary information
