# Supplementary material for: Single-cell RNA-based phenotyping reveals a pivotal role of thyroid hormone receptor alpha for hypothalamic development
Source: Development. 2023 Jan 30;150(3):dev201228. doi: 10.1242/dev.201228 (PMC10110490; doi:10.1242/dev.201228)
Supplement: Supplementary file 1 [file develop-150-201228_review_history.pdf]

Single-cell RNA-based phenotyping reveals a pivotal role of thyroid hormone receptor alpha for hypothalamic development

Varun K. A. Sreenivasan, Riccardo Dore, Julia Resch, Julia Maier, Carola Dietrich, Jana Henck, Saranya Balachandran, Jens Mittag and Malte Spielmann

DOI: 10.1242/dev.201228

Editor: Samantha Morris

Review timeline

Original submission:	23 August 2022
Editorial decision:	30 September 2022
First revision received:	21 November 2022
Editorial decision:	19 December 2022
Second revision received:	21 December 2022
Accepted:	23 December 2022

Original submission

First decision letter

MS ID#: DEVELOP/2022/201228

MS TITLE: Single-cell RNA based phenotyping reveals a pivotal role of thyroid hormone receptor alpha for hypothalamic development

AUTHORS: Varun K A Sreenivasan, Riccardo Dore, Julia Resch, Julia Maier, Carola Dietrich, Saranya Balachandran, Jana Henck, Jens Mittag, and Malte Spielmann

I have now received all the referees' reports on the above manuscript, and have reached a decision. The referees' comments are appended below, or you can access them online: please go to BenchPress and click on the 'Manuscripts with Decisions' queue in the Author Area.

As you will see, the referees express considerable interest in your work, but have some significant criticisms and recommend a substantial revision of your manuscript before we can consider publication. If you are able to revise the manuscript along the lines suggested, which may involve further experiments, I will be happy receive a revised version of the manuscript. Your revised paper will be re-reviewed by one or more of the original referees, and acceptance of your manuscript will depend on your addressing satisfactorily the reviewers' major concerns. Please also note that Development will normally permit only one round of major revision. If it would be helpful, you are welcome to contact us to discuss your revision in greater detail. Please send us a point-by-point response indicating your plans for addressing the referee's comments, and we will look over this and provide further guidance.

Please attend to all of the reviewers' comments and ensure that you clearly highlight all changes made in the revised manuscript. Please avoid using 'Tracked changes' in Word files as these are lost in PDF conversion. I should be grateful if you would also provide a point-by-point response detailing how you have dealt with the points raised by the reviewers in the 'Response to Reviewers' box. If you do not agree with any of their criticisms or suggestions please explain clearly why this is so.

© 2023. Published by The Company of Biologists under the terms of the Creative Commons Attribution License (https://creativecommons.org/licenses/by/4.0/).

Reviewer 1

Advance summary and potential significance to field

Sreenivasan and colleagues use single-nucleus RNA-seq profiling to investigate defects across individual cell types of the hypothalamus due to hypothyroidism induced by a hypomorphic allele of TRa1. Single-nucleus RNA-seq revealed only a small effect of hypothyroidism on the transcriptional phenotype of hypothalamic neuronal populations. However, the authors find a substantial change in hypothalamic oligodendrocytes, which authors propose is due to a developmental defect where oligodendrocytes do not reach full maturation. Moreover, the authors show that a post-natal increase in thyroid signaling can rescue this phenotype.
The study is well written, and the conclusions are largely supported. The study is of interest to the developmental field and has clear translational importance.
However, the analysis of single-nucleus RNA-seq data is largely superficial.

Comments for the author

Major comments to be addressed:

- The authors note a cluster of cells expressing both astrocytic and oligodendrocytic markers. Doublet scoring suggests that these are doublets. The authors remove these cells but comment on an expectation on mitochondrial reads that suggests these may not be doublets specifically, that the percentage of mitochondrial reads is expected to be similar. To the best of my knowledge, this is not an established measure. There could be technical reasons that give rise to this higher percentage of mitochondrial reads in doublets, especially given the results of the simulation of doublets with scrublet. Without further evidence this statement could be cited as a way to ignore high doublet scores.

- The claim of little differences in neuronal populations is a strong claim given their profiling is missing a population of neurons that has previously been described as TRa1 dependent (it does not seem the authors probed for this cell type via immunohistochemistry) and the lack of differential expression analyses by genotype (see point 3).

- The authors focus largely on UMAP embeddings and cell type proportions. They do not explore differential expression as a function of genotype across the entire dataset (i.e., across all cell types). It is unclear whether batch effects were accounted for in the differential expression analysis on genotypes present for oligodendrocytes. The authors do not discuss genes they identify as differentially expressed, except for a few new oligodendrocyte markers. The manuscript would be strengthened by an in-depth discussion of dynamic genes as a function of genotype.
The major conclusions could also be gained by immunoblot analysis of major hypothalamic cell types probing for known marker genes.

- The lack of intermediate marker expression does not rule out the existence of intermediate states. Still, it could be due to lower sensitivity or low capture of these genes in their particular dataset. Dataset integration analysis with existing datasets, at the very least those of the hypothalamus, is preferable to determine the lack of a cell state.

Minor comments:

- It is unclear why the authors do not explore and/or apply batch correction globally before cell type assignment. The authors only plot data by replicates across sub-UMAPs. Given teh exclusion of a cluster due to batch effects before batch correction, the authors should present the effect of batch effects earlier in the manuscript.

- Grayscale color bars for expression in marker gene dot plots are hard to distinguish.

- Gene expression differences should not be visualized only on UMAPs. Violin plots by cluster and genotype are preferred to highlight quantitative differences in expression.

© 2023. Published by The Company of Biologists under the terms of the Creative Commons Attribution License (https://creativecommons.org/licenses/by/4.0/).

Reviewer 2

Advance summary and potential significance to field

The study aims to characterize the role of TRalpha1 in the development of various cell populations in the mouse hypothalamus. The authors performed single-nucleus RNA sequencing on the hypothalamus of adult WT and TRalpha1 heterozygous mutant mice to compare the frequencies of individual cell types and their gene expression profiles. While they do not detect differences in the composition of cell types, they do identify a number of differentially expressed genes in oligodendrocytes. Next, they took homogenates of hypothalamic tissue and used western blotting to analyze genes known to play a role in oligodendrocyte biology. The authors find that when TRalpha1 signaling is diminished, there is a reduction in proteins involved in myelination. They demonstrate that this reduction can be abolished again when TRaplha1 signaling is postnatally restored.
In general, the data provided by the authors support several claims of the study. Single-nucleus transcriptomic profiling permits a largely unbiased and comprehensive characterization of cell populations and demonstrates that the overall tissue composition is not affected by defects in TRalpha1 signaling. After correcting for batch effects in the data, the authors discovered substantial differences in gene expression specifically in oligodendrocytes. The study further shows that myelination proteins are reduced, confirming that the oligodendrocyte population is affected in TRalpha1 mutants. However, the authors fail to connect the results from single-nucleus analysis sufficiently to the findings from the western blotting. Though the individual findings are valuable and interesting, this makes the two parts of the study seem disconnected which leaves some of the conclusions insufficiently supported. Under the condition that the authors address the major comments listed below, I would consider the study conclusive and valuable for the scientific community. I therefore, recommend this manuscript be subjected to a revision, at which point it will likely be suitable for publication.

Comments for the author

Major comments (essential):

1. The authors need to provide some account of how the findings from the single-nucleus analysis (first part of the study, Figure 1-3) are linked to those from the protein level (second part of the study, Figure 4-5) to clarify their claims about how the oligodendrocyte population is affected. They should explain the rationale by which they selected specific genes for profiling by western blotting and, whenever possible, present levels of both transcripts and proteins for these genes. The following changes/additions would strengthen the connection between the findings of the two parts of the manuscript.
Single-nucleus analysis: The differential gene expression analysis between the two genotypes in oligodendrocytes yielded a list of ~90 deregulated genes (line 161-165). Only 10 of these are mentioned and presented in Figure 3B. The complete list needs to be provided as supplementary information. Additionally for all the genes analyzed by western blotting, the authors should present and discuss the extent to which their transcript levels are affected in oligodendrocytes (feature plots as in Figure 3B). Many of the genes identified by western blotting might also exhibit differences in abundance at the transcript level. This information would help the reader to connect the findings regarding transcripts and proteins.
Western blotting: Only three of the differentially expressed genes identified in the single-nucleus analysis were analyzed by western blotting, and deregulation at the protein level could only be shown for one of them (TMEM117). In the current version of the paper, these three genes are the only link between the two parts of the study, which is insufficient and does not support a conclusion. No rationale was given for how the other genes (CNPase, MOG, PLP1, ODSP) were selected and what role the single-nucleus analysis results played in this selection. Did the transcriptional analysis indicate that they were deregulated? This information needs to be provided within the main text and can be included in Supplementary Table 1.

2. A major finding of the manuscript suggests that oligodendrocyte maturation is impaired, but the results presented so far do not sufficiently support this claim. There is no specific result to support the statement that TRalpha1 signaling “seems to be crucial for hypothalamic oligodendrocyte maturation” (line 41 in abstract; line 82, 250). The specific findings and reasoning

© 2023. Published by The Company of Biologists under the terms of the Creative Commons Attribution License (https://creativecommons.org/licenses/by/4.0/).

that allow the authors to arrive at this conclusion should be laid out in detail. Further, the statement “very relevant for completion of maturation, as evidenced by the clearly distinct UMAP populations” (line 250) is invalid; a 2D visualization alone cannot reveal impairments in maturation. Instead, the differentially expressed genes (line 161-165) should be analyzed to address whether maturation is indeed impaired, or whether only methylation is impaired without additional signs of a stalled cell maturation process. Figures 4 and 5 show that proteins involved in myelination are reduced (e.g. MOG, PLP1); however, there is no discussion regarding the extent to which myelination can be considered an approximation for maturation. Additionally, it should be investigated whether these genes are also reduced at the transcript level of oligodendrocytes using the single-nucleus data. To what extent maturation is affected requires more analysis, especially since the authors did not identify clusters reflecting oligodendrocyte maturation stages (Supplementary Figure 8). The authors should then formulate the way in which impaired TRalpha1 signaling affects oligodendrocytes as precisely as possible, by clearly differentiating between conclusions, indications, and assumptions.

Note: Addressing the major comments 1 and 2 above would require a further investigation of the genes found to be differentially expressed in the single-nucleus data from oligodendrocytes. The requirement that only genes detected in at least one in five cells (line 166-167) might be too stringent for data with on average 2,000 genes/nucleus. The authors could test whether a lower cut-off (e.g., detected in one in ten cells) recovers more genes involved in myelination and maturation.

Recommended changes and extensions:

To explain potential cell doublets, the authors refer to a study that reported cell-type specific doublets in an entirely different context (line 116-117, Xu et al. 2014). Specific cell types that preferentially attach (forming doublets) should not occur in single-nucleus RNA sequencing because all cells enter the experiment as nuclei.

The authors sometimes use the terminology “cellular architecture” (line 126, 195) when they refer to the cell type composition of the tissue. I recommend avoiding the term “architecture” as it often refers to spatial organization and thus can be misunderstood.

The authors point out that they could not identify a cell population (parvalbumin neurons) that they previously reported as being affected (line 148-150, line 229-231). They explain this by referring to the low abundance of this cell type. However, more information needs to be provided. The authors have sequenced ~30,000 nuclei from WT hypothalamus. According to their previous findings (Mittag et al. 2013), how many parvalbumin neurons would they expect to find in those 30,000 cells? Which genes were used to look for these cells? The feature plots should be provided in the supplement.

The selection of proteins in Figure 4A,B (CNPase, MOG, PLP1, ODSP; line 174), the biological role of these genes, and the interpretation of their reduction need to be explained. The reasoning behind profiling these proteins and the connection to the single-nucleus analysis results are unclear. Furthermore, since the proteins were analyzed from bulk samples (tissue homogenates), the authors should first show their cell type-specific expression across the hypothalamus cell types using the single-nucleus data (comparable to Figure 1C). Such a supplementary figure will illustrate that these proteins can be profiled in bulk to acquire information specifically about oligodendrocytes.

The strategy by which TRalpha1 signaling is restored postnatally and pre-/postnatally needs to be explained in more detail (line 182-186). To a reader unfamiliar with thyroid hormone signaling defects, it will be unclear how defects in another TH receptor (TRbeta) can restore TRalpha1 signaling.

Feature plots (UMAPs showing the expression of an individual gene) are only shown for the top ten differentially expressed genes in Figure 3B. However, these feature plots should also be provided for all genes profiled via western blotting.

© 2023. Published by The Company of Biologists under the terms of the Creative Commons Attribution License (https://creativecommons.org/licenses/by/4.0/).

Figure 4B,D and 5B,D: The three data points per group need to be shown individually, e.g. plotting them instead of the bar plots or plotting them on the bar plots (see also Weissgerber et al. 2015).

Figure 5: It is unclear what “wildtype” refers to: Are the hypothalamus samples from WT mice or mice carrying a WT TRalpha1 gene in a TRbeta knockout background? This needs to be clarified in the figure legend and the labeling within the figures. What is presented is presumably “WT TRalpha1 gene in a TRbeta knockout”; in this case, the protein levels should also be directly compared with those of WT mice (presented in Figure 4), and the authors should commend on potential differences between these two “WT” references.

Minor comments:

Line 26: “in cellular development” Could mean anything and should be specified.
Line 86: extend title beyond the method that is being used Line 90: change to “nuclei per sequenced sample” to avoid that the numbers are misunderstood as experimental input.
Line 103: it should be “by differential gene expression analysis”
Line 121: Provide percentages when referring to “this very small cluster”
Line 139: sentence is discontinued “batch effects since…”
Line 159: “oligodendrocytes formed two distinct clusters”. It seems like no additional clustering was performed and the authors should instead refer to two distinctly distributed groups of cells within the oligodendrocyte cluster.
Line 176: “newly identified marker genes”. These are not marker genes for a specific cell type (at least no data was presented that would suggest cell type specificity) and to avoid misunderstandings the authors could consistently refer to these genes as “differentially expressed genes”.
Line 236: “TH” should be spelled out before the abbreviation is used.
Line 325-339: Provide exact information which functions were used from which packages/versions.

Supplementary Figure 7: The cell type compositions of the data sets are compared based on the original clustering and annotations of the respective studies (i.e. no data integration was performed before making a comparison). This should be mentioned.

Figure 4C and 5C: Please add the missing loading controls for the Western blots.

Figure 4B: For CNPase, please check whether the difference is indeed significant between WT and TRalpha1 mutant (the average levels appear very similar)

Supplementary Table 1: The meaning of the asterisk should be included in the figure legend.

First revision

Author response to reviewers' comments

Our responses to the reviewer’s comments in blue and any edits made to the manuscript in *“brown italics”.*

Reviewer 1 Advance Summary and Potential Significance to Field:

Sreenivasan and colleagues use single-nucleus RNA-seq profiling to investigate defects across individual cell types of the hypothalamus due to hypothyroidism induced by a hypomorphic allele of TRa1. Single-nucleus RNA-seq revealed only a small effect of hypothyroidism on the transcriptional phenotype of hypothalamic neuronal populations. However, the authors find a substantial change in hypothalamic oligodendrocytes, which authors propose is due to a developmental defect where oligodendrocytes do not reach full maturation. Moreover, the authors show that a post-natal increase in thyroid signaling can rescue this phenotype. The study is well written, and the conclusions are largely supported. The study is of interest to the developmental

© 2023. Published by The Company of Biologists under the terms of the Creative Commons Attribution License (https://creativecommons.org/licenses/by/4.0/).

field and has clear translational importance. However, the analysis of single-nucleus RNA-seq data is largely superficial.
We thank the reviewer for their interest in our manuscript and the positive feedback. We have addressed all the comments raised by the reviewer, as below

Reviewer 1 Comments for the Author:

Major comments to be addressed:

- The authors note a cluster of cells expressing both astrocytic and oligodendrocytic markers. Doublet scoring suggests that these are doublets. The authors remove these cells but comment on an expectation on mitochondrial reads that suggests these may not be doublets specifically, that the percentage of mitochondrial reads is expected to be similar. To the best of my knowledge, this is not an established measure. There could be technical reasons that give rise to this higher percentage of mitochondrial reads in doublets, especially given the results of the simulation of doublets with scrublet. Without further evidence, this statement could be cited as a way to ignore high doublet scores.

We agree with the reviewer that our argument of using the mitochondrial composition of the transcriptome to distinguish doublets is not standard practice. Therefore, we have removed this aspect of the discussion. We note, however, that a similar cluster characterized by the expression of oligodendrocyte- and astrocyte- marker genes was labeled to be doublets by Mickelsen et al 2019 Nat. Neurosci. Nevertheless, given the high doublet scores of these scales, we erred on the side of caution to not include this cluster for further analysis. The edited sentences read as follows:

> *A small, seventh cluster of 207 cells appeared to express several astrocytic and oligodendrocytic marker genes and was initially labeled to be non-neuronal cells. Specifically, of the 929 positively differentially expressed genes in this non-neuronal cluster, 408 and 500 genes were shared with the differentially expressed genes of oligodendrocytes and astrocytes, respectively. A similar cluster was also observed in the lateral hypothalamic area by Mickelsen et al (in their Supplementary Fig 2) (Mickelsen et al., 2019), which was annotated as doublets in their dataset. Indeed, these cells do exhibit a higher doublet score distribution in our dataset (**Fig. S2**). Although, the formation of such cell type specific doublets constituting astrocytes and oligodendrocytes is unlikely in our dataset due to the use of single-nuclei sequencing (as opposed to single-cell sequencing by Mickelsen et al), we erred on the side of caution and decided not to further analyze this small cluster (0.37% of total nuclei).*

- The claim of little differences in neuronal populations is a strong claim given their profiling is missing a population of neurons that has previously been described as TRa1 dependent (it does not seem the authors probed for this cell type via immunohistochemistry) and the lack of differential expression analyses by genotype (see point 3).

Thank you for this important comment. We agree with the reviewer that it was disappointing to not observe an effect that was previously observed by us (namely, the reduction in the number of parvalbuminergic neurons) (Mittag et al J. Clin. Invest. 2013). Therefore, first of all, we refined our data exploration to identify these neurons. We now included a second marker for these genes in addition to *Pvalb*, namely *Syt2* (Sommeijer and Levelt PLoS One 2012). The subsequent analysis of the cellular composition reveals a definite drop in the cell numbers in the mutant hypothalamus. We have added this analysis as a new **Fig. S6** and discuss the findings in the manuscript as below:

> *To address the first question, as to whether parvalbuminergic neurons are less abundant in the TRa1+m hypothalamus (Mittag et al., 2013), we identified these cells based on the expression of Pvalb and Syt2 - known markers of parvalbuminergic neurons (Sommeijer and Levelt, 2012) (**Fig. S6A-D**). Indeed, the total number of parvalbuminergic neurons dropped from 66 in the wild type to 16 in the mutant hypothalamus, which after correcting for the total number of neurons per dataset amounts to a drop in abundance by 3.6 fold (**Fig. S6E**). However, given the generally low abundance of these neurons and the limited experimental repeats, the difference in cell numbers were not found to be statistically significant. Perhaps for similar reasons, a differential gene expression analysis between the genotypes for this neuronal population did not yield any significantly differentially expressed genes (data not shown).*

© 2023. Published by The Company of Biologists under the terms of the Creative Commons Attribution License (https://creativecommons.org/licenses/by/4.0/).

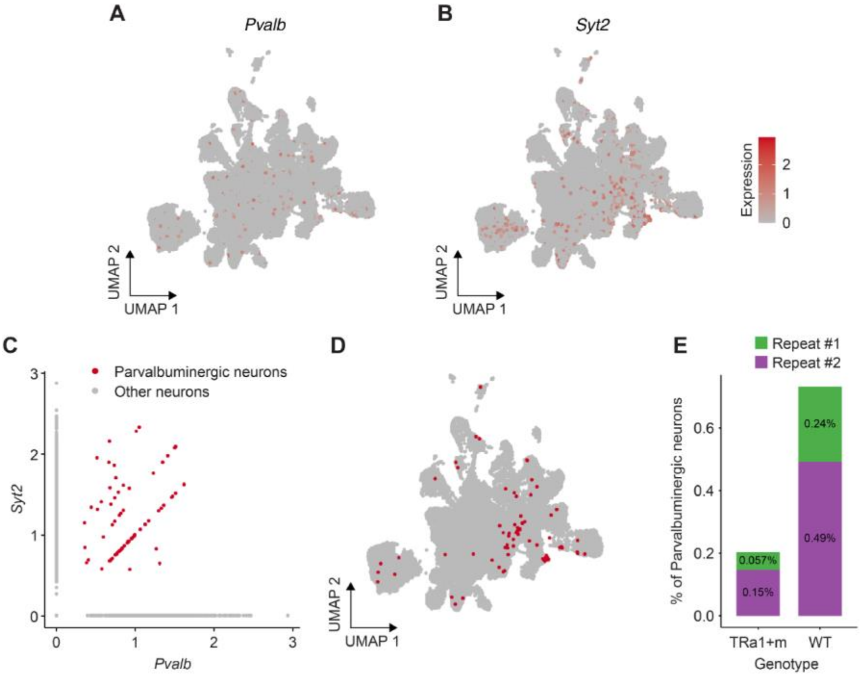

__Figure S6__. __Cellular composition of parvalbuminergic neurons__. __A-B__. UMAP embedding of the Neurons_1 cluster highlighting the expression of the marker genes of parvalbuminergic neurons (Pvalb, Syt2) __C__. Pvalb+/Syt2+ neurons are defined as Pvaralbuminergic neurons. __D__. UMAP embedding of the Neurons_1 cluster highlighting the parvalbuminergic neurons (red). __C__. The cellular composition of parvalbuminergic neurons as a percentage of the total number of neurons in each of the four datasets (to account for varying cell numbers between the datasets).

Furthermore, as discussed in our response to the next comment, we also performed DE analysis on the entire dataset across all annotated clusters/sub-clusters, the results of which are summarized in the **Fig. S10** (pasted below). This figure clearly displays the eclipsing nature of the effect of the TRA mutation on the oligodendrocytes when compared to all the other annotated cell types. This is the reason why we focussed our efforts on oligodendrocytes in the latter parts of our manuscript. A discussion on the DE genes identified for all neuronal subclusters have also been added to the main text, as follows:

Differential gene expression analysis (materials and methods, __Tables S2,S3__) between the two genotypes across all neuronal sub-clusters revealed seven genes (after additional p-value-based filtering), of which AC149090.1 (Phosphatidylserine Decarboxylase, Pisd) was present in 8 of the 14 neuronal sub-clusters and was also the only gene that was expressed higher in the TRα1+m than the wild type neurons. The other six appeared in only one (Grm8, Hcn1, Lrrtm4, mt-Atp6, and Oxt) or two (mt-Co3) sub-clusters each, and to our knowledge have not been linked to thyroid hormone signaling previously, while Pisd plays a role in lipid metabolism, a pathway known to be affected by thyroid signaling in the hypothalamus (López et al., 2010; Pucci et al., 2000). Overall, based on the gene expression data and the similar expression of several neuronal markers tested by in situ hybridization and immunohistochemistry including Argp, Npy, Pomc, Crh, Hcrt, Gad67 or NeuN (__Fig. S7__), the effect of the mutant TRα1 on hypothalamic neuronal development seems to be subtle.

© 2023. Published by The Company of Biologists under the terms of the Creative Commons Attribution License (https://creativecommons.org/licenses/by/4.0/).

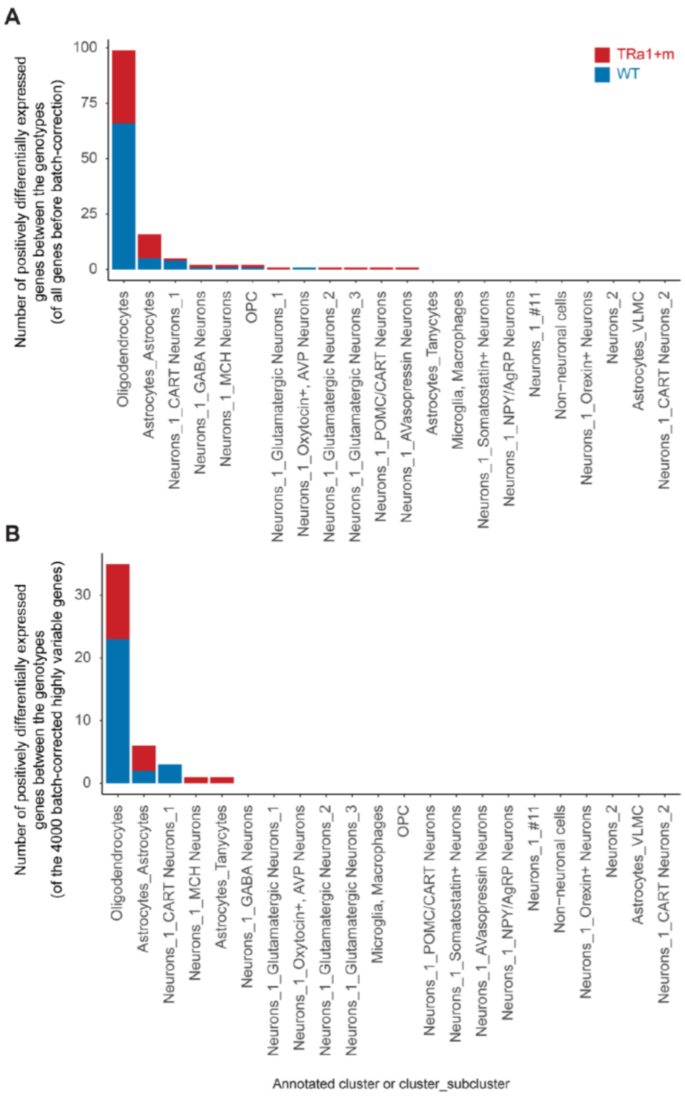

Figure S10. Number of differentially expressed genes per annotated clusters or sub-clusters*. The number of differentially expressed genes between the two genotypes were calculated* ***A.*** *without bach-correction across all the genes or* ***B.*** *with Seurat's canonical correlation analysis-based batch-correction of the top 4000 highly variable genes. Differential expression analysis was performed based on the Wilcoxon Rank Sum test for genes expressed in at least 10% of the cells of either genotypes, and was further filtered for the absolute average log2 fold change greater than 0.5 and bonferroni adjusted p-values < 1E-20. Positively differentially expressed genes of either genotypes are colored in blue and red for the two genotypes.*

As recommended by the reviewer, we have also added the requested immunostaining for parvalbumin in both genotypes to **Fig. S7** (pasted below), showing the expected lower number of Pvalb neurons in the TRa1+m mutant mice. Furthermore, we have added a NeuN immunostaining as marker for neurons in general, which also did not show any obvious difference between the two genotypes.

© 2023. Published by The Company of Biologists under the terms of the Creative Commons Attribution License (https://creativecommons.org/licenses/by/4.0/).

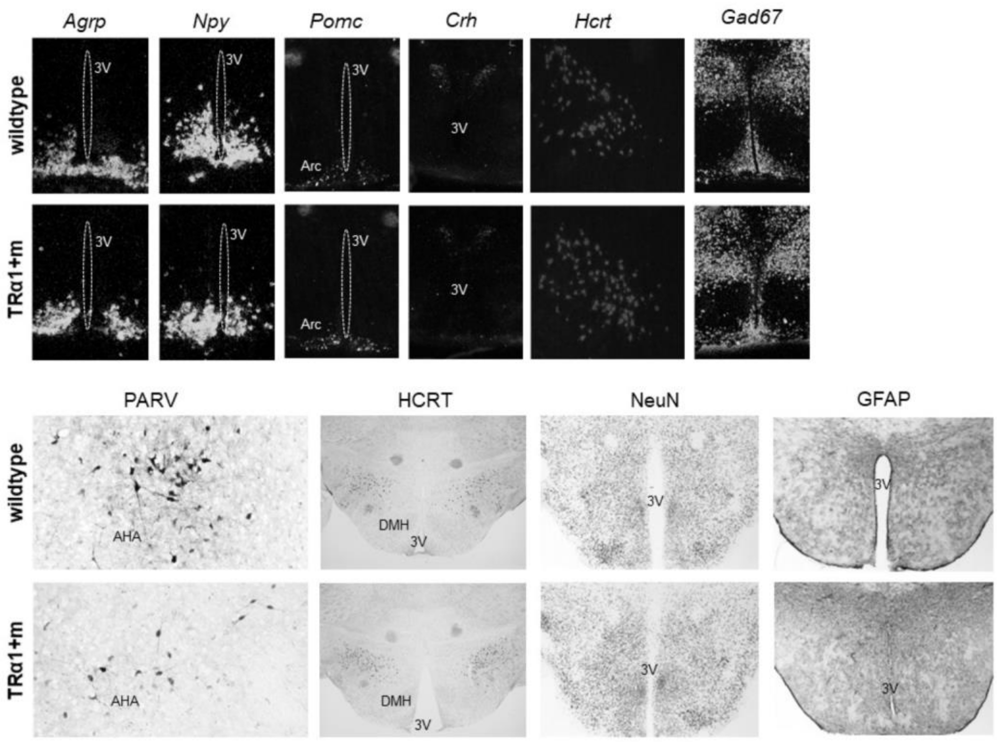

*Figure S7. Expression of marker genes. (Top) In situ hybridisation for agouti-related peptide (Agrp), neuropeptide Y (Npy), proopiomelanocortin (Pomc), corticotropin-releasing hormone (Crh), Orexin (Hcrt), and glutamate decarboxylase 67 (Gad67) as well as (**Bottom**) immunohistochemistry (IH) for HCRT, neuronal nuclei (NeuN) and glial fibrillary acidic protein (GFAP) reveals similar expressions of classic hypothalamic markers between wildtype and TRα1+m mutants, with the expected exception of parvalbumin (PARV). 3V: third ventricle of the hypothalamus, Arc: arcuate nucleus, DMH: dorsomedial hypothalamus.*

- The authors focus largely on UMAP embeddings and cell type proportions. They do not explore differential expression as a function of genotype across the entire dataset (i.e., across all cell types). It is unclear whether batch effects were accounted for in the differential expression analysis on genotypes present for oligodendrocytes. The authors do not discuss genes they identify as differentially expressed, except for a few new oligodendrocyte markers. The manuscript would be strengthened by an in-depth discussion of dynamic genes as a function of genotype. The major conclusions could also be gained by immunoblot analysis of major hypothalamic cell types probing for known marker genes.

We thank the reviewer for this recommendation and constructive feedback. We had relied on UMAP embeddings to dictate our analysis strategy, because UMAPs tend to portray the major changes (because the dimensionality reduction is performed based on highly variable genes). Indeed, as pointed by the reviewer, this approach will not be sensitive to smaller changes. Therefore, as mentioned in our reply to the previous comment, we have performed DE analysis across the entire dataset for every annotated cluster or sub-cluster (**Fig. S10**, pasted in the above reply). Likewise, we added the requested immunoblot and immunohistochemistry analyses for major hypothalamic cell types, namely TUBB3, SYP and GFAP (Western) and PVALB, NeuN and GFAP (Immunohistochemistry) (**Fig. S7**, pasted in the above reply). The results have been included in the manuscript and the discussion. The implemented changes are below:

> *Overall, based on the gene expression data and the similar expression of several neuronal markers tested by in situ hybridization and immunohistochemistry including Argp, Npy, Pomc, Crh, Hcrt, Gad67 or NeuN (**Fig. S7**), the effect of the mutant TRα1 on hypothalamic neuronal development seems to be subtle.*

Regarding whether batch-correction was applied prior to DE analysis. Since the primary batch correction method we use is harmony, which does not alter the raw gene expression values, the

© 2023. Published by The Company of Biologists under the terms of the Creative Commons Attribution License (https://creativecommons.org/licenses/by/4.0/).

differential gene expression analysis did not consider the batch effects in the first version of the manuscript. However, we have now also performed batch correction on the gene expression data of the top 4000 highly variable genes across the two batches on the entire dataset, per annotated cluster/sub-cluster, using Seurat's canonical correlation analysis method. We now present the list of DE genes both before and after batch-correction as **Tables S2 and S3** and the associated figure is added as a new **Fig. S10** (pasted above). The main text has also been updated to reflect this as follows:

> *Oligodendrocytes are affected by the disrupted thyroid receptor function We performed similar harmony-based batch-correction and UMAP embedding of all the cell types (**Figs 3A,S8,S9**). Amongst them, oligodendrocytes (**Fig. 3A**) and, to a smaller extent, astrocytes and oligodendrocyte progenitor cells (**Fig. S9A,B**) exhibited segregated accumulation of cells from the two genotypes in the UMAP embeddings. A differential gene expression analysis between the two genotypes across all the annotated clusters and sub-clusters also revealed oligodendrocytes to be the most affected cell type, with 99 differentially expressed genes after filtering (materials and methods, **Fig. S10A** and **Table S2**). To rule out the possibility that this apparent oligodendrocyte-centric effect of the genotype is caused by batch effects (because harmony-based batch-correction is performed in the PCA low-dimensional space and does not provide a corrected gene-expression matrix), we performed Seurat's canonical correlation analysis-based batch-correction on the gene-expression matrix corresponding to the top 4000 highly variable genes, on which the differential expression analysis was performed (**Fig. S10B, Table S3**). While the absolute number of genes identified was lower than without batch-correction (because only 4000 highly variable genes were considered in the batch-correction), oligodendrocytes were still the most affected cell type.*

- The lack of intermediate marker expression does not rule out the existence of intermediate states. Still, it could be due to lower sensitivity or low capture of these genes in their particular dataset. Dataset integration analysis with existing datasets, at the very least those of the hypothalamus, is preferable to determine the lack of a cell state.

We thank the reviewer for this suggestion to better demonstrate the lack of intermediate cells during oligodendrocyte differentiation. We took advantage of the recently published HypoMap dataset containing curated data from 17 hypothalamus single-cell and single-cell studies (Steuernagel et al 2022 Nat. Metab.). This figure is included as a part of **Fig. S16** (pasted below), which shows that our data does indeed have a severely low number of intermediate states. The discussion as well as the materials and methods sections has also been updated accordingly. The discussion section has also been updated as follows:

> *This was further confirmed by integrating our data to the recently published curated hypothalamus single-cell atlas, HypoMap (Steuernagel et al., 2022) (**Fig. S16D,E**).*

© 2023. Published by The Company of Biologists under the terms of the Creative Commons Attribution License (https://creativecommons.org/licenses/by/4.0/).

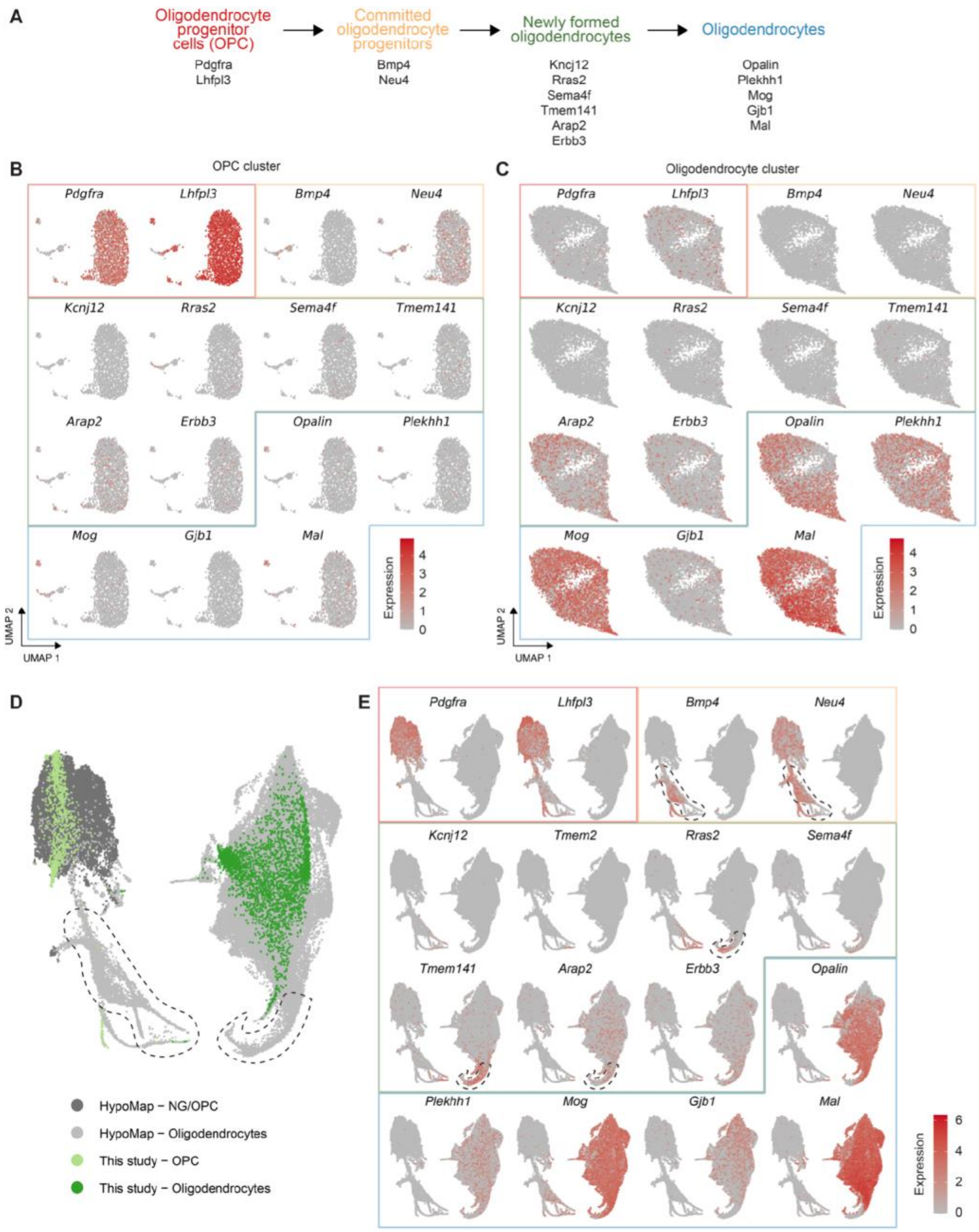

Figure S16. Expression and the lack thereof of marker genes during the differentiation and myelination of oligodendrocytes*. A. Four stages of oligodendrocyte formation and the marker genes at each stage* *(Chen et al., 2017; La Manno et al., 2018)*. *B,C. The lack of expression of the known markers of committed oligodendrocyte progenitors (COL) and newly formed oligodendrocytes (NFOL) confirm the lack of these intermediate cells in our dataset. D. UMAP embedding of our oligodendrocyte and OPC cells integrated with the curated HypoMap dataset, highlighting the lack of COL and NFOL in our dataset as demarcated by their absence within the dashed black regions, which correspond to the expression patterns of markers shown in E. E. Expression of the markers in the integrated data.*

Minor comments:

- It is unclear why the authors do not explore and/or apply batch correction globally before cell type assignment. The authors only plot data by replicates across sub-UMAPs. Given the exclusion of a cluster due to batch effects before batch correction, the authors should present the effect of batch effects earlier in the manuscript.

© 2023. Published by The Company of Biologists under the terms of the Creative Commons Attribution License (https://creativecommons.org/licenses/by/4.0/).

We thank the reviewer for highlighting this discontinuity in presenting our data. We had originally not shown the batch correction at the level of the entire dataset, because the batch effects at this level did not affect the cell type identification. As per the reviewer's suggestion, (i) we now include the UMAP embeddings of the full dataset based on repeats as the new **Fig S3**. As can be seen, while the data from the two repeats do not overlap well, the cluster boundaries are consistent between the repeats. (ii) Furthermore, we also carried out harmony-based integration on the full dataset (as opposed to individual clusters), followed by clustering and DE gene analysis for cellular annotation, with an aim to identify whether the cell type annotation is affected as a result of batch effects. These results are presented as **Fig. S4**. As can be seen, the annotations of only 30 of the 56k cells in the entire dataset changed. The new figures are:

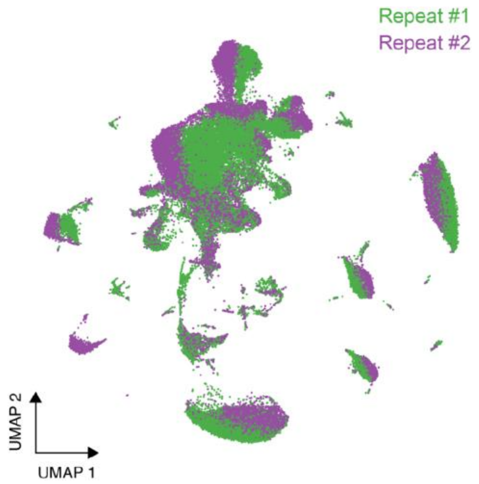

Figure S3. Batch effects are present in the data. *UMAP embeddings of the entire dataset without batch-correction, where cells are colored by experimental repeat.*

© 2023. Published by The Company of Biologists under the terms of the Creative Commons Attribution License (https://creativecommons.org/licenses/by/4.0/).

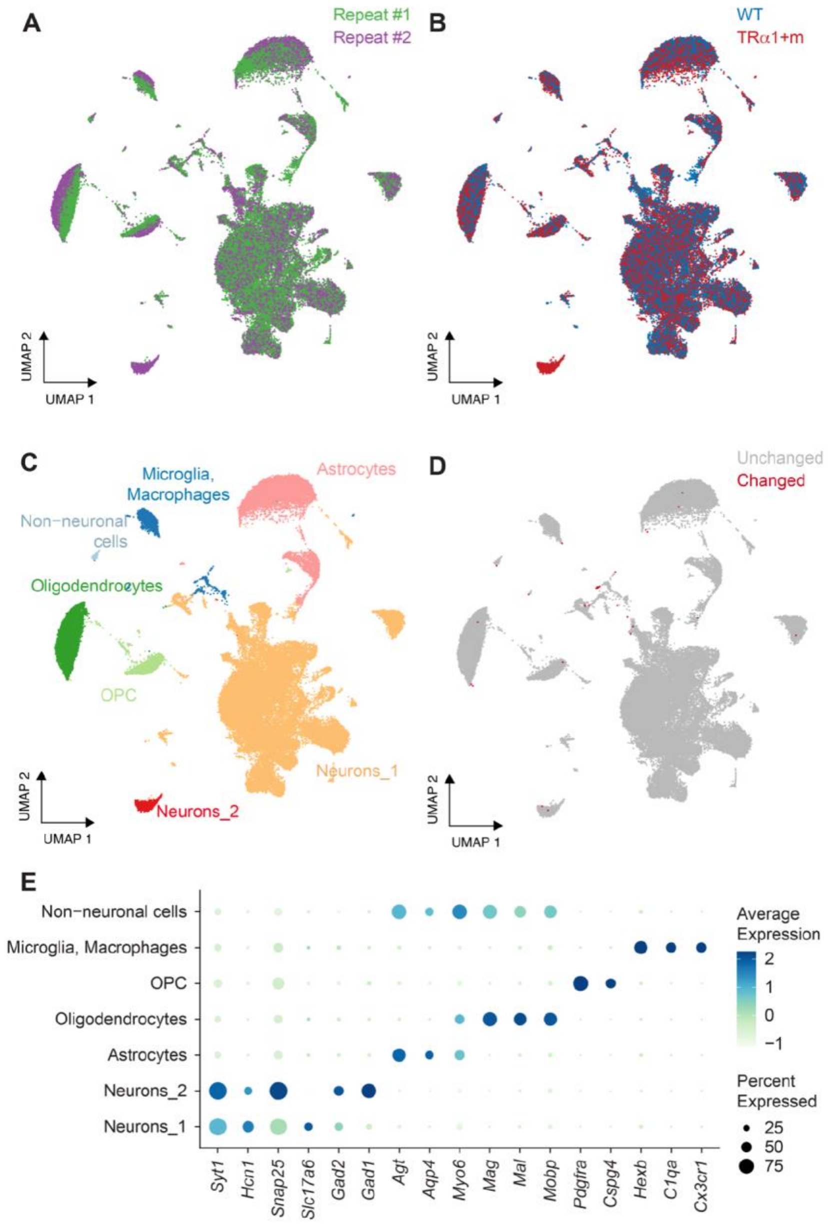

Figure S4. Harmony-based batch-correction at the entire dataset level does not affect clustering or cell type annotation. *A-C. UMAP embeddings of the entire dataset after harmony-based batch-correction, where cells are colored by A. experimental repeat, B. genotype, or C. cell type. D. The cell type annotation of 30 cells (red) changed as a result of harmony-based batch-correction. E. The differentially expressed genes between the clusters also remained unchanged in comparison to* ***Fig. 1C****.*

- Grayscale color bars for expression in marker gene dot plots are hard to distinguish.

We thank the reviewer for this comment. We have replaced the gray color bars throughout the manuscript for improved contrast.

- Gene expression differences should not be visualized only on UMAPs. Violin plots by cluster and genotype are preferred to highlight quantitative differences in expression.

Violin plots showing the quantitative differences in the gene expression between the two genotypes in the oligodendrocytes are presented as a series of three supplementary figures (**Figs S11,S12,S13**). For all other annotated clusters and sub-clusters, the data is made available in **Tables S2,S3**. As an example, the **Fig. S11** is pasted below:

© 2023. Published by The Company of Biologists under the terms of the Creative Commons Attribution License (https://creativecommons.org/licenses/by/4.0/).

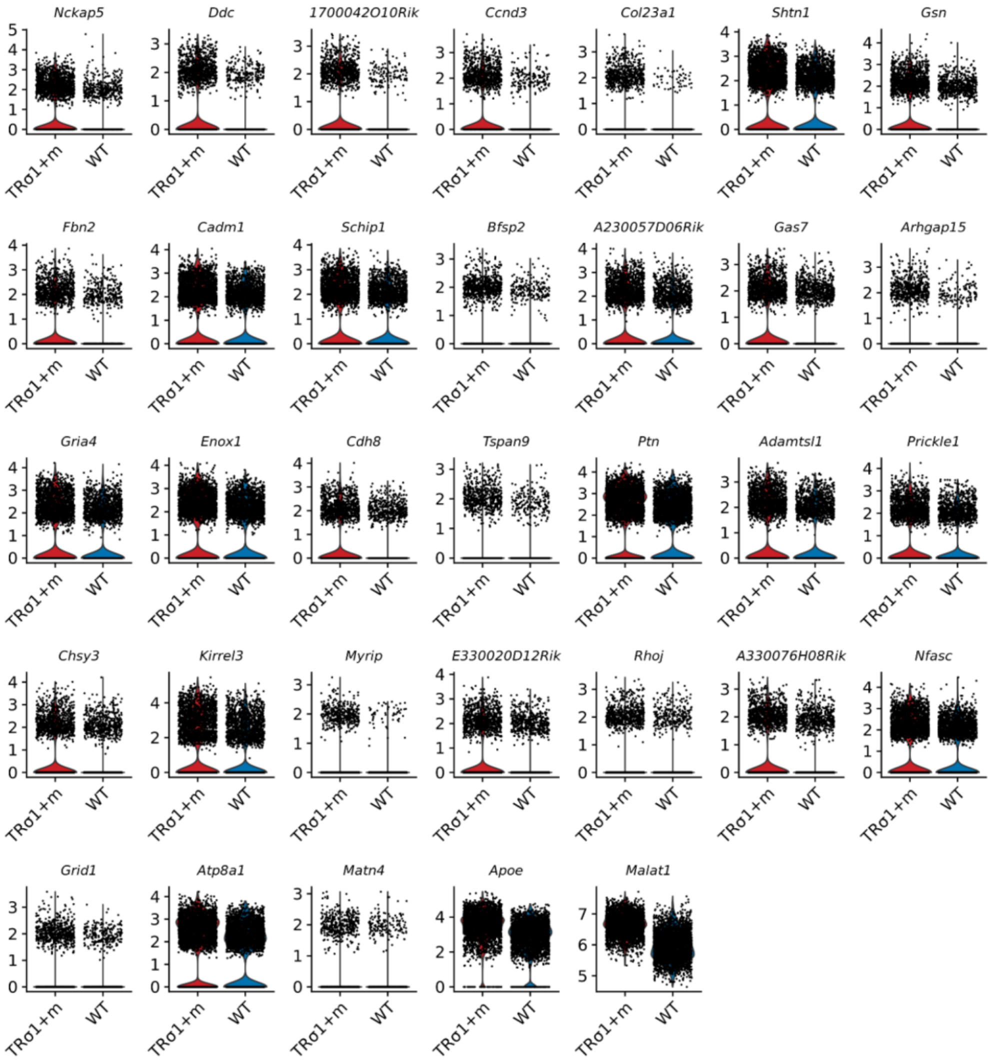

Figure S11. Expression of differentially expressed genes between the genotypes in oligodendrocytes. *Positively differentially expressed genes in the TRα1+m, sorted by decreasing order of the difference in percentage of cells expressing the gene.*

Reviewer 2 Advance Summary and Potential Significance to Field:

The study aims to characterize the role of TRalpha1 in the development of various cell populations in the mouse hypothalamus. The authors performed single-nucleus RNA sequencing on the hypothalamus of adult WT and TRalpha1 heterozygous mutant mice to compare the frequencies of individual cell types and their gene expression profiles. While they do not detect differences in the composition of cell types, they do identify a number of differentially expressed genes in oligodendrocytes. Next, they took homogenates of hypothalamic tissue and used western blotting to analyze genes known to play a role in oligodendrocyte biology. The authors find that when TRalpha1 signaling is diminished, there is a reduction in proteins involved in myelination. They demonstrate that this reduction can be abolished again when TRaplha1 signaling is postnatally restored.

In general, the data provided by the authors support several claims of the study. Single-nucleus transcriptomic profiling permits a largely unbiased and comprehensive characterization of cell populations and demonstrates that the overall tissue composition is not affected by defects in TRalpha1 signaling. After correcting for batch effects in the data, the authors discovered substantial differences in gene expression specifically in oligodendrocytes. The study further shows that myelination proteins are reduced, confirming that the oligodendrocyte population is affected in TRalpha1 mutants. However, the authors fail to connect the results from single-nucleus analysis sufficiently to the findings from the western blotting. Though the individual findings are

© 2023. Published by The Company of Biologists under the terms of the Creative Commons Attribution License (https://creativecommons.org/licenses/by/4.0/).

valuable and interesting, this makes the two parts of the study seem disconnected, which leaves some of the conclusions insufficiently supported. Under the condition that the authors address the major comments listed below, I would consider the study conclusive and valuable for the scientific community. I therefore, recommend this manuscript be subjected to a revision, at which point it will likely be suitable for publication.

We thank the reviewer for their interest in our manuscript and the positive feedback. We have addressed all the comments raised by the reviewer, as below.

Reviewer 2 Comments for the Author:

Major comments (essential):

1. The authors need to provide some account of how the findings from the single-nucleus analysis (first part of the study, Figure 1-3) are linked to those from the protein level (second part of the study, Figure 4-5) to clarify their claims about how the oligodendrocyte population is affected. They should explain the rationale by which they selected specific genes for profiling by western blotting and, whenever possible, present levels of both transcripts and proteins for these genes. The following changes/additions would strengthen the connection between the findings of the two parts of the manuscript.

We thank the reviewer for this important issue and apologize for not better explaining the rationale for the second part. The single cell approach was used as an unbiased method to identify alterations in the hypothalamic cellular composition in TRa mutant mice to find populations that depend on TRa1 signaling for development (similar to the previously described parvalbumin neurons in the AHA, Mittag et al. 2013 JCI). This analysis revealed that oligodendrocytes are the most affected cell type, as observed by the cellular distribution in the UMAP embeddings as well as the number of DE genes identified in oligodendrocytes (with respect to all the other cell types, new **Fig. S10**, pasted below) Therefore it felt warranted to focus primarily on oligodendrocytes for the more in-depth second part. Given that mRNA translation is heavily regulated in oligodendrocytes on several levels including mRNA editing (Xu et al. 2020 Neuron), integrin mediated activation of the 3'UTR (Laursen, 2011 J Cell Biol), mRNA transport (Müller et al 2013 Frontiers in Cell Neuro), tRNA modification and codon-based mRNA decay (Martin et al 2022 Nat Comm) and small non-coding RNAs (Bauer et al. 2012 EMBO Reports), we decided to probe for established oligodendrocyte proteins from different stages of development to better define the fate of this cell type. The probed proteins include OLIG2 and SOX10 for all stages of development, PLP and CNPase for stages from pre-Oligodendrocytes, and MBP, MOG, ODSP and ARSG for mature oligodendrocytes. These results demonstrated that the oligodendrocyte precursor cells seem to be fine, but the mature oligodendrocytes are somewhat defective (given the alterations in PLP1, CNPase, MOG and ODSP), but generally present (as evidenced by the normal MBP and ARSG). This conclusion is also supported by the very small number of differentially expressed genes (two) between the genotypes in OPC as opposed to 99 in oligodendrocytes. This is in agreement with the fact that we could not match the expression signature of the TRa1 mutant oligodendrocyte population to published lineage data from oligodendrocyte development (Kim et al 2016 Nat. Commun.), as they seem to develop in general, but with different properties.

To take this comment into account, we have now revised the manuscript accordingly to better present the rationale of the second part of the study and to discuss the apparent differences between mRNA and protein levels using the references above for the heavy posttranscriptional regulation occurring in oligodendrocytes. The main text has been updated as follows:

> *Given that oligodendrocytes seemed to be the most severely affected cell population in TRa1+m mutant mice, we focused on this cell type for a more in-depth analysis. Since mRNA translation is heavily regulated in oligodendrocytes on several levels including mRNA editing (Xu et al. 2020), integrin mediated activation of the 3'UTR (Laursen et al., 2011), mRNA transport (Müller et al., 2013), tRNA modification and codon-based mRNA decay (Martin et al., 2022) as well as small non-coding RNAs (Bauer et al., 2012), we decided to probe for established oligodendrocyte proteins from different stages of development to better define the fate of this cell type, namely OLIG2 and SOX10 for all stages of development, PLP and CNPase for stages from pre-Oligodendrocytes, and MBP, MOG, ODSP and ARSG for mature oligodendrocytes. We therefore included an additional*

© 2023. Published by The Company of Biologists under the terms of the Creative Commons Attribution License (https://creativecommons.org/licenses/by/4.0/).

*cohort of mice from both genotypes that were treated for 2 weeks as adults with thyroid hormone T3 in drinking water to restore acute TRα1 signaling. While SOX10, MBP, ARSG and OLIG2 were not affected by genotype or T3 treatment (**Fig. 4, Table S1**), we observed a reduction of CNPase, MOG, PLP1 and ODSP in the TRα1+m mice, which was however not restored when TRα1 was reactivated by T3, indicating a permanent developmental defect. The newly identified differentially expressed genes Tmem117, Dpyd, Slco3a1 and Hcn2 (**Fig. 3**) were also tested on protein level, confirming a reduction in DPYD and HCN2 that was not rescued by T3 treatment, whereas TMEM117 and SLCO3A1 were not affected on protein level in either conditions (**Fig. 4, Table S1**).*

The newly added figure:

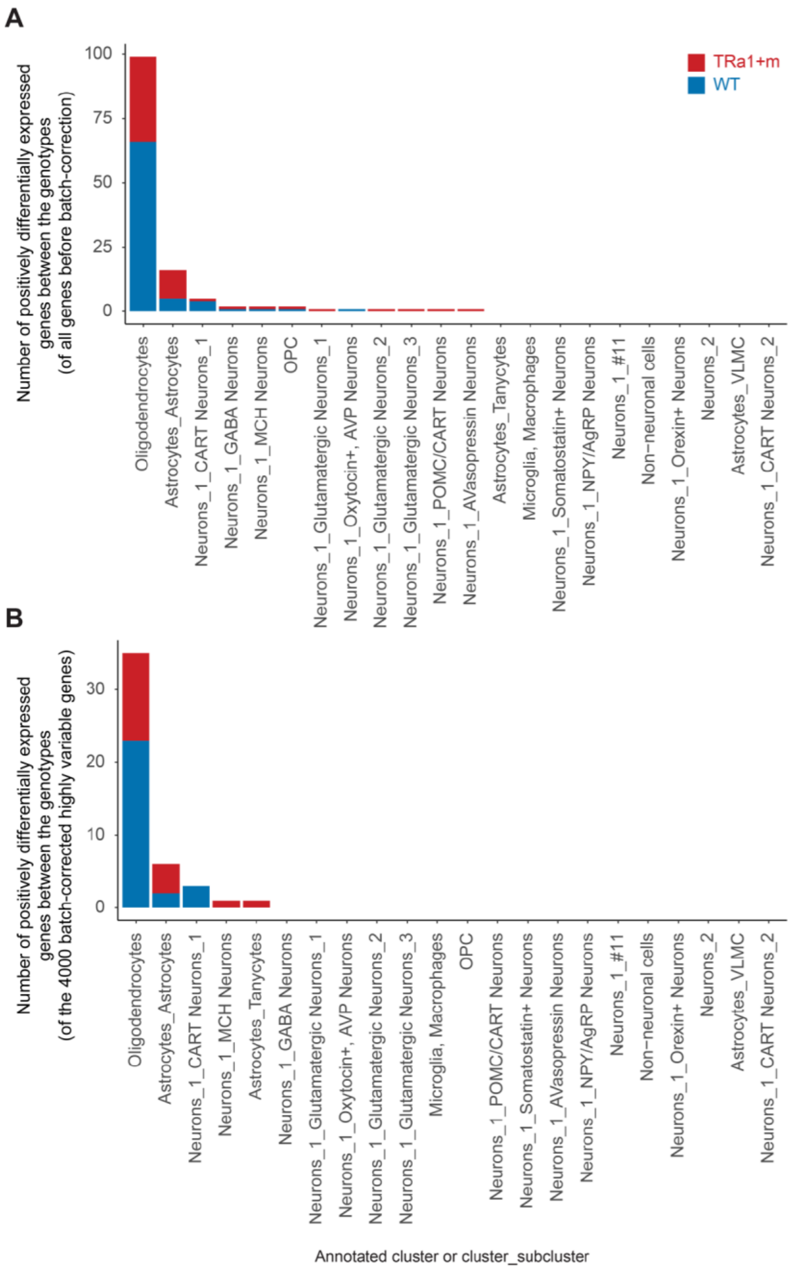

***Figure S10. Number of differentially expressed genes per annotated clusters or sub- clusters.** The number of differentially expressed genes between the two genotypes were calculated A. without bach-correction across all the genes or B. with Seurat's canonical correlation analysis-based batch-correction of the top 4000 highly variable genes. Differential expression analysis was performed based on the Wilcoxon Rank Sum test for genes expressed in at least 10% of the cells of either genotypes, and was further filtered for the absolute average log2 fold change greater than 0.5 and bonferroni*

© 2023. Published by The Company of Biologists under the terms of the Creative Commons Attribution License (https://creativecommons.org/licenses/by/4.0/).

adjusted p- values < 1E-20. Positively differentially expressed genes of either genotypes are colored in blue and red for the two genotypes.

Single-nucleus analysis: The differential gene expression analysis between the two genotypes in oligodendrocytes yielded a list of ~90 deregulated genes (line 161-165). Only 10 of these are mentioned and presented in Figure 3B. The complete list needs to be provided as supplementary information. Additionally, for all the genes analyzed by western blotting, the authors should present and discuss the extent to which their transcript levels are affected in oligodendrocytes (feature plots as in Figure 3B). Many of the genes identified by western blotting might also exhibit differences in abundance at the transcript level. This information would help the reader to connect the findings regarding transcripts and proteins. Western blotting: Only three of the differentially expressed genes identified in the single-nucleus analysis were analyzed by western blotting, and deregulation at the protein level could only be shown for one of them (TMEM117). In the current version of the paper, these three genes are the only link between the two parts of the study, which is insufficient and does not support a conclusion. No rationale was given for how the other genes (CNPase, MOG, PLP1, ODSP) were selected and what role the single-nucleus analysis results played in this selection. Did the transcriptional analysis indicate that they were deregulated? This information needs to be provided within the main text and can be included in Supplementary Table 1.

We thank the reviewer for the opportunity to clarify our rationale in the revised manuscript. As explained above, we used the most apparent abnormality from the single cell study, namely the oligodendrocyte population, as the starting point for the in-depth study in the second part. This second part aimed to address three questions:

1) How is the oligodendrocyte development affected in the TRa mutant mice?

Given the plethora of posttranscriptional modifications occurring in this cell type as listed above, we decided to use Western Blot for well established oligodendrocyte marker proteins as a more direct readout of the developmental stage. This analysis revealed that the early markers SOX10 and OLIG2 are not changed and that even later markers for mature oligodendrocytes are expressed correctly (MBP and ARSG). Other late markers (PLP1, CNPase, MOG, ODSP) are however lower in TRa mutants, suggesting that the oligodendrocytes develop to maturity, but remain somehow different. This has been clarified in the revised manuscript, as follows:

> *Similarly, in our study we found normal protein expression of SOX10 and OLIG2 as early markers and MBP and ARSG as markers for mature oligodendrocytes, while other protein markers such as PLP1, CNPase, MOG and ODSP were still lower in TRα1+m mice. Remarkably, our study identified several other genes that were disrupted in this cell type at the transcript level, and some of these including the sodium/potassium channel HCN2 and the dihydropyrimidine dehydrogenase DPYD could be confirmed on the protein level. While our findings support the notion that mRNA and protein level do not necessarily match in oligodendrocytes, as expected from the heavily regulated translation in the cell type (Xu et al. 2020; Laursen et al. 2011; Müller et al. 2013; Martin et al. 2022; Bauer et al. 2012), both technologies confirm a generally altered appearance of mature oligodendrocytes in TRα1+m mice. In agreement with observations in congenital hypothyroid children (Gupta et al., 1995), these findings suggest that thyroid hormone may be of less importance for the start of the myelination process, but very relevant for the completion of maturation, as evidenced by the number of differentially expressed genes and the clearly distinct UMAP embeddings between the oligodendrocytes from wildtype and TRα1+m mice.*

2) How are these OLs different in TRa mutant mice?

Here we used the DE gene list as the starting point for further immunoblot studies, which we have provided as a series of three supplementary figures (**Figs S11,S12,S13**). The raw data is also provided as **Tables S2,S3**. As the reviewer is most certainly aware, it is not always possible to find suitable antibodies for all candidate genes, which is why only Tmem117, Dpyd and Slco3a1 were included in **Fig. 4**. We have now tested additional antibodies for the DE genes and were able to get reliable data also for HCN2, which is now included in the new figure (pasted below) and shows the predicted reduction. The analysis in general confirms the single cell data, but also shows that posttranscriptional regulation or protein stability might heavily affect oligodendrocytic properties. This has been discussed in the revised manuscript (as pasted above).

© 2023. Published by The Company of Biologists under the terms of the Creative Commons Attribution License (https://creativecommons.org/licenses/by/4.0/).

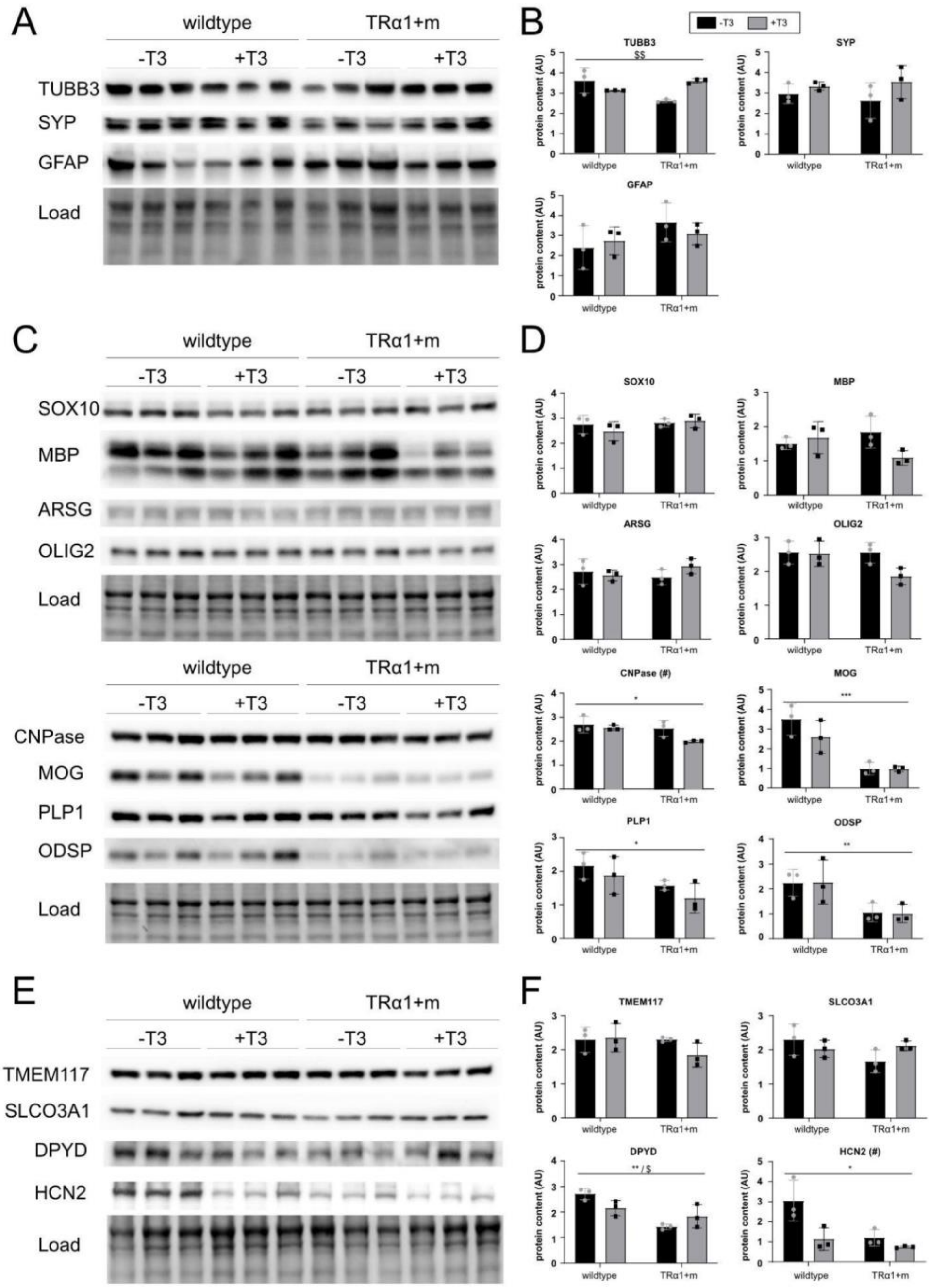

Figure 4 Comparison of the protein-expression of cellular and oligodendrocyte markers from whole-hypothalamus homogenates in adult wildtype and TRα1+m mutant, with and without T3 treatment. *A,C,E Raw western blots and their* ***B,D,F*** *quantifications for the expression of A,B overall hypothalamic cell markers, C,D classic oligodendrocyte markers and E,F four potentially differentially expressed genes between the WT and the mutant. *: p<0.05; **:p<0.01; ***:p<0.001 for wildtype versus TRα1+m mutant, #:p<0.05 for T3-treatment, $:p<0.05, $$:p<0.01 for interaction between T3 treatment and genotype (2-way ANOVA, n=3 per group,* ***Table S1****).*

3) At what time during development does this impairment occur?

As oligodendrocyte development occurs in several waves starting at E14, with peak oligodendrocyte production at 2-4wks postnatally and also still to some extent in adulthood (Bergles 2016 Cold Spring Harbor Perspectives and Kuhn et al. 2019 Cells), we decided to a) test whether the adult production can be rescued by T3 (it cannot) and b) whether the pre- or postnatal waves can be rescued (postnatal yes, prenatal no additional effect). We have now clarified the

© 2023. Published by The Company of Biologists under the terms of the Creative Commons Attribution License (https://creativecommons.org/licenses/by/4.0/).

rationale for the times used in the second part as well in the revised manuscript, as below:.

*Based on these observations, we hypothesized that impaired TRα1 signaling during an earlier developmental period would be responsible for this irreversible defect, as oligodendrocyte development in mice occurs in several perinatal waves. To this end, we used our established approach of backcrossing the TRα1+m mice to the TRβknockout strain, as these mice are endogenously hyperthyroid in postnatal life (TRα1+m TRβ-/- double knockouts). Therefore, any reversal of a TRα1+m phenotype on the TRβ-/- background then confirms that it occurs postnatally and is furthermore independent of TRβ signaling. As a second group, we included TRα1+m TRβ-/- double knockouts, that are born by hyperthyroid TRβ-/- knockout mothers. These dams are hyperthyroid throughout pregnancy and thus can reactivate the mutant TRα1 additionally during embryonic development (Wallis et al., 2008) to test for reversal in this period. The resulting animals were then analyzed as adults (**Fig. 5, Table S1**). Our data unequivocally show that the postnatal reactivation of TRα1 signaling leads to an unaltered expression of the previously impaired expression of CNPase, MOG, PLP1 and ODSP when comparing the TRα1++ and TRα1+m genotypes, while the additional reactivation before birth in TRβ-/- dams has little effect. Likewise, DPYD and HCN2 were no longer lower in TRα1+m as compared to the respective controls. Interestingly, TMEM117, which was not affected by the TRα1 mutation on the protein level, was lowered in both groups that were exposed to elevated thyroid hormone prenatally.*

2. A major finding of the manuscript suggests that oligodendrocyte maturation is impaired, but the results presented so far do not sufficiently support this claim. There is no specific result to support the statement that TRalpha1 signaling "seems to be crucial for hypothalamic oligodendrocyte maturation" (line 41 in abstract; line 82, 250). The specific findings and reasoning that allow the authors to arrive at this conclusion should be laid out in detail. Further, the statement "very relevant for completion of maturation, as evidenced by the clearly distinct UMAP populations" (line 250) is invalid; a 2D visualization alone cannot reveal impairments in maturation. Instead, the differentially expressed genes (line 161-165) should be analyzed to address whether maturation is indeed impaired, or whether only methylation is impaired without additional signs of a stalled cell maturation process. Figures 4 and 5 show that proteins involved in myelination are reduced (e.g. MOG, PLP1); however, there is no discussion regarding the extent to which myelination can be considered an approximation for maturation. Additionally, it should be investigated whether these genes are also reduced at the transcript level of oligodendrocytes using the single-nucleus data. To what extent maturation is affected requires more analysis, especially since the authors did not identify clusters reflecting oligodendrocyte maturation stages (Supplementary Figure 8).

The authors should then formulate the way in which impaired TRalpha1 signaling affects oligodendrocytes as precisely as possible, by clearly differentiating between conclusions, indications, and assumptions.

We agree with the reviewer that we were not as precise as necessary in defining the oligodendrocytic defect in the original manuscript, and we appreciate the opportunity to correct this in the revised version. The reviewer is entirely correct that myelination cannot be used as an approximation for maturation; more importantly, only some marker proteins are affected (PLP1, CNPase, MOG, ODSP), while others such as MBP - a classic marker for myelination - is normal. We therefore conclude now that the oligodendrocytes do progress through the entire developmental process, given the normal marker expression of SOX10, OLIG2 and MBP; however, they present with phenotypic abnormalities including lower PLP1, CNPAse, MOG and ODSP marker levels in addition to the difference found on transcript (list of DE genes now provided in full as **Tables S2,S3** and **Figs S11,S12,S13;** as an example, **Fig. S12** is pasted below) and protein level (DPYD and now HCN2, **Fig. 4** pasted above). As these defects are not rescued by T3 treatment in adulthood, they are not simply an acute change in gene expression caused by the mutant TRα1, but rather a consequence of a defect founded during early postnatal development. It seems therefore more precise to characterize the observed defect as a deviation of the phenotype of mature OLs in TRa1 mutant mice that originates from an early postnatal action of the mutant TRa1 during oligodendrocyte development, rather than stating that they are stuck during development given that they express some markers for mature OLs.

The following lines have been added/edited in the discussion section:

© 2023. Published by The Company of Biologists under the terms of the Creative Commons Attribution License (https://creativecommons.org/licenses/by/4.0/).

While our findings support the notion that mRNA and protein level do not necessarily match in oligodendrocytes, as expected from the heavily regulated translation in the cell type (Xu et al. 2020; Laursen et al. 2011; Müller et al. 2013; Martin et al. 2022; Bauer et al. 2012), both technologies confirm a generally altered appearance of mature oligodendrocytes in TRα1+m mice. In agreement with observations in congenital hypothyroid children (Gupta et al., 1995), these findings suggest that thyroid hormone may be of less importance for the start of the myelination process, but very relevant for the completion of maturation, as evidenced by the number of differentially expressed genes and the clearly distinct UMAP embeddings between the oligodendrocytes from wildtype and TRα1+m mice.

The new **Fig. S12**:

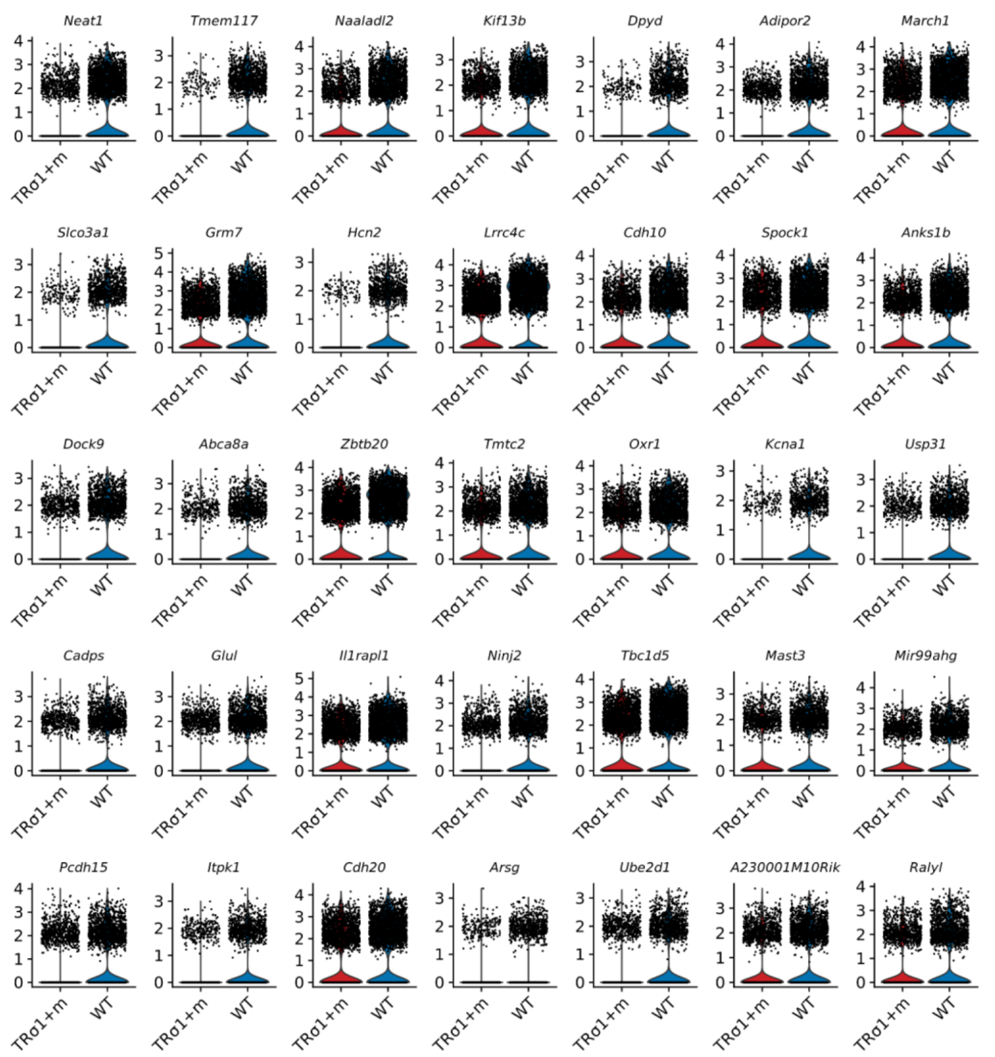

***Figure S12. Expression of differentially expressed genes between the genotypes in oligodendrocytes**. Positively differentially expressed genes in the wild type, sorted by decreasing order of the difference in percentage of cells expressing the gene. Continued in **Fig. S13**.*

Note: Addressing the major comments 1 and 2 above would require a further investigation of the genes found to be differentially expressed in the single-nucleus data from oligodendrocytes. The requirement that only genes detected in at least one in five cells (line 166-167) might be too stringent for data with on average 2,000 genes/nucleus. The authors could test whether a lower cut-off (e.g., detected in one in ten cells) recovers more genes involved in myelination and maturation.

© 2023. Published by The Company of Biologists under the terms of the Creative Commons Attribution License (https://creativecommons.org/licenses/by/4.0/).

We have changed the filtering criteria of the DE genes between genotypes to be to one in ten cells, throughout the manuscript. This resulted in moderate increase in the number of DE genes identified (now 99 genes). The details of DE gene calculation between genotypes has been added as another subsection ("Differential gene expression analysis across genotypes") in the materials and methods. We did search through GOBP gene sets corresponding to oligodendrocyte differentiation, development, regulation and proliferation genesets, but none of the DE genes except *Ptn* was found in our DE genes list after stringent statistical cutoff of adjusted p-value < 1E-20 and log2FC>0.5. Moreover, a gene set enrichment analysis also did not result in any interpretable process or pathways.

The changes made to the manuscript are as follows:

> ***Differential gene expression analysis across genotypes***
>
> *All of the following steps were carried out using Seurat-4.0.5 as well as standard R packages. We determined the DE features between the wildtype and TRa1+m expressing cells across all the annotated cell clusters or sub-clusters using the FindAllMarkers function using the following parameters: min.cells.group=0, min.pct=0.1, and logfc_threshold=0.5. The obtained gene list was further filtered for statistical significance using bonferroni adjusted p-values < 1E-20. This stringent cut off was chosen to reduce false positives. The differential expression analysis was performed on the raw (non-batch corrected) expression data, since batch-correction based on harmony applies only to the PCA space. However, as a means of verification, DE analysis was also performed on batch-corrected expression data. For this purpose, Seurat's canonical correlation analysis-based batch-correction was applied over the top 4000 highly variable genes reduced to 20 PCs. The results of the DE analysis without and with batch-correction without post-filtering are presented in* ***Tables S2,S3****.*

Recommended changes and extensions:

To explain potential cell doublets, the authors refer to a study that reported cell-type specific doublets in an entirely different context (line 116-117, Xu et al. 2014). Specific cell types that preferentially attach (forming doublets) should not occur in single-nucleus RNA sequencing because all cells enter the experiment as nuclei.
We agree with the reviewer's comment. We have now removed the reference and the associated argument. We instead refer to the work by Mickelsen et al 2019 Nat. Neurosci., where a similar cluster was identified in the hypothalamus, in which both astrocytic and oligodendrocytic markers are expressed. Whether this cluster is truly a doublet is unclear, but we nevertheless exclude the cells from further analysis. The new text is as follows:

> *A small, seventh cluster of 207 cells appeared to express several astrocytic and oligodendrocytic marker genes and was initially labeled to be non-neuronal cells. Specifically, of the 929 positively differentially expressed genes in this non-neuronal cluster, 408 and 500 genes were shared with the differentially expressed genes of oligodendrocytes and astrocytes, respectively. A similar cluster was also observed in the lateral hypothalamic area by Mickelsen et al (in their Supplementary Figure 2) (Mickelsen et al., 2019), which was annotated as doublets in their dataset. Indeed, these cells do exhibit a higher doublet score distribution in our dataset (****Fig. S2****). Although, the formation of such cell type specific doublets constituting astrocytes and oligodendrocytes is unlikely in our dataset due to the use of single-nuclei sequencing (as opposed to single-cell sequencing by Mickelsen et al), we erred on the side of caution and decided not to further analyze this small cluster (0.37% of total nuclei).*

The authors sometimes use the terminology "cellular architecture" (line 126, 195) when they refer to the cell type composition of the tissue. I recommend avoiding the term "architecture" as it often refers to spatial organization and thus can be misunderstood.
We have replaced the wording to be more specific. We now refer to the cellular composition and transcriptomic profiles.

The authors point out that they could not identify a cell population (parvalbumin neurons) that they previously reported as being affected (line 148-150, line 229-231). They explain this by referring to the low abundance of this cell type. However, more information needs to be provided. The

© 2023. Published by The Company of Biologists under the terms of the Creative Commons Attribution License (https://creativecommons.org/licenses/by/4.0/).

authors have sequenced ~30,000 nuclei from WT hypothalamus. According to their previous findings (Mittag et al. 2013), how many parvalbumin neurons would they expect to find in those 30,000 cells? Which genes were used to look for these cells? The feature plots should be provided in the supplement.

We thank the reviewer for this comment. Based on their suggestion, we have now performed a detailed calculation of the expected cell numbers: the total number of AHA parvalbumin neurons in a wildtype hypothalamus is around 400 cells (Mittag et al 2013 JCI). With the published cell density of the hypothalamus of around 300.000 cells/mm^3 (Keller et al. 2018 Frontiers in Neuroanatomy) and the volume of the excised hypothalamus of approx 3,22mm * 2,5 mm * 1,5mm (depth, width, height) = 12,075mm^3, we would have 3,6 Mio cells total in this area. For 30.000 nuclei that were sequenced, this would come down to less than ten parvalbumin neurons included - not accounting for any bias in the nuclei extraction for different cell types etc.

We had originally only used the expression of *Pvalb* in our efforts to identify the parvalbuminergic neurons of our interest. However, this resulted in 606 WT neurons, indicating that this marker alone is not sufficiently specific to identify these neurons. Therefore, as also suggested by the other reviewer, we refined our approach to identify these neurons using an additional marker gene (*Syt2*), which resulted in 66 WT and 16 TRα1 neurons, thus reflecting the expected reduction in the number of these neurons (Mittag et al. 2013 JCI). This number is still a bit higher than our estimation above, but is likely a result of biases in nuclei extraction or residual marker unspecificity.

As requested, the feature plots of the marker genes are provided in the new **Fig. S6** (pasted below), which also indicates a drop in the number of these neurons in the mutant hypothalamus.

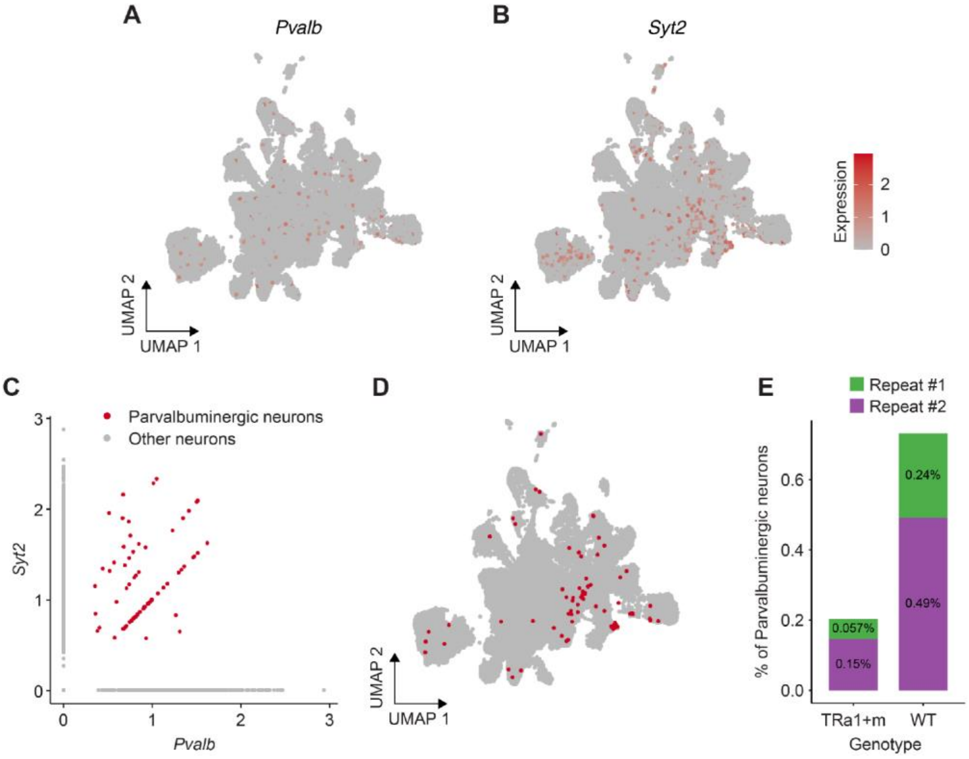

***Figure S6**. **Cellular composition of parvalbuminergic neurons**. **A-B**. UMAP embedding of the Neurons_1 cluster highlighting the expression of the marker genes of parvalbuminergic neurons (Pvalb, Syt2) **C**. Pvalb+/Syt2+ neurons are defined as Pvaralbuminergic neurons. **D**. UMAP embedding of the Neurons_1 cluster highlighting the parvalbuminergic neurons (red). **C**. The cellular composition of parvalbuminergic neurons as a percentage of the total number of neurons in each of the four datasets (to account for varying cell numbers between the datasets).*

© 2023. Published by The Company of Biologists under the terms of the Creative Commons Attribution License (https://creativecommons.org/licenses/by/4.0/).

The results section has also been edited as follows:

*To address the first question, as to whether parvalbuminergic neurons are less abundant in the TRa1+m hypothalamus (Mittag et al., 2013), we identified these cells based on the expression of Pvalb and Syt2 - known markers of parvalbuminergic neurons (Sommeijer and Levelt, 2012) (**Fig. S6A-D**). Indeed, the total number of parvalbuminergic neurons dropped from 66 in the wild type to 16 in the mutant hypothalamus, which after correcting for the total number of neurons per dataset amounts to a drop in abundance by 3.6 fold (**Fig. S6E**). However, given the generally low abundance of these neurons and the limited experimental repeats, the difference in cell numbers were not found to be statistically significant. Perhaps for similar reasons, a differential gene expression analysis between the genotypes for this neuronal population did not yield any significantly differentially expressed genes (data not shown).*

The selection of proteins in Figure 4A,B (CNPase, MOG, PLP1, ODSP; line 174), the biological role of these genes, and the interpretation of their reduction need to be explained. The reasoning behind profiling these proteins and the connection to the single-nucleus analysis results are unclear. Furthermore, since the proteins were analyzed from bulk samples (tissue homogenates), the authors should first show their cell type-specific expression across the hypothalamus cell types using the single-nucleus data (comparable to Figure 1C). Such a supplementary figure will illustrate that these proteins can be profiled in bulk to acquire information specifically about oligodendrocytes.

We thank the reviewer for this feedback. We have now added additional cell type marker proteins including TUBB3, SYP and GFAP (new **Fig. 4**, pasted above) to demonstrate that bulk analysis in hypothalamic homogenates is possible and replicates the sequencing findings for neurons and astrocytes. With regard to oligodendrocytes, we selected classic protein markers for these cells and different stages during their development (see comment above). The selection of these markers and their biological role during oligodendrocyte development has been explained in more depth in the revised manuscript.

We have also included the single-cell expression corresponding to all the proteins tested in Figures 4 and 5, as evidence that performing WB on tissue homogenate for most of these genes/proteins provides a reasonable representation of their expression in oligodendrocytes alone (to the extent the proteome mirrors the transcriptome). This is added as **Fig. S14A** (pasted below).

© 2023. Published by The Company of Biologists under the terms of the Creative Commons Attribution License (https://creativecommons.org/licenses/by/4.0/).

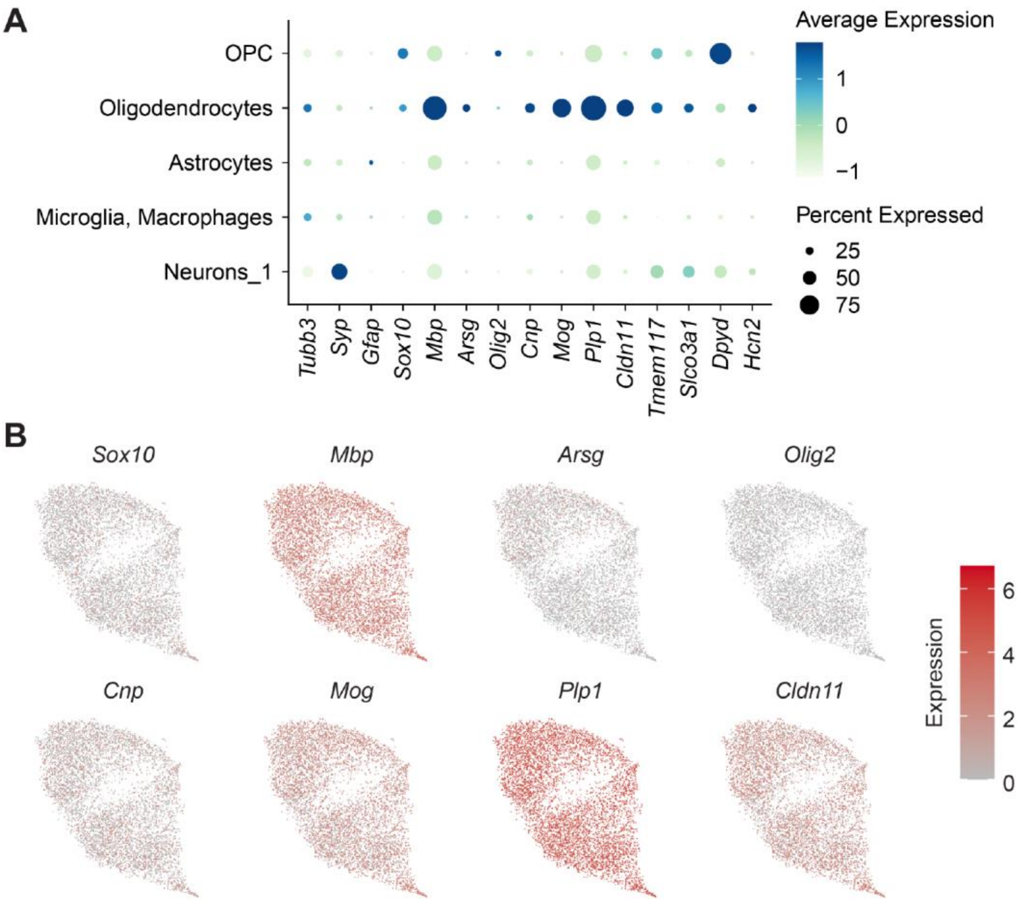

Figure S14. Gene expression of proteins assayed by western blotting in Figs 4,5. A. **Expression of most of the markers are limited to the respective cells. Only cells from the wild type hypothalamus were included, since some of these genes are differentially expressed in the TRα1+m oligodendrocytes.** B. **Expression pattern based on the same UMAP embedding as in** Fig. 3. **Cldn11 codes for ODSP.**

The strategy by which TRalpha1 signaling is restored postnatally and pre-/postnatally needs to be explained in more detail (line 182-186). To a reader unfamiliar with thyroid hormone signaling defects, it will be unclear how defects in another TH receptor (TRbeta) can restore TRalpha1 signaling.

We apologize for not being more precise in the explanation of the double mutant strategy to reactivate the mutant TRa1 and have elaborated in more detail in the revised version of the manuscript, which now reads:

*Based on these observations, we hypothesized that impaired TRα1 signaling during an earlier developmental period would be responsible for this irreversible defect, as oligodendrocyte development in mice occurs in several perinatal waves. To this end, we used our established approach of backcrossing the TRα1+m mice to the TRβ knockout strain, as these mice are endogenously hyperthyroid in postnatal life (TRα1+m TRβ-/- double knockouts). Therefore, any reversal of a TRα1+m phenotype on the TRβ-/- background then confirms that it occurs postnatally and is furthermore independent of TRβ signaling. As a second group, we included TRα1+m TRβ-/- double knockouts, that are born by hyperthyroid TRβ-/- knockout mothers. These dams are hyperthyroid throughout pregnancy and thus can reactivate the mutant TRα1 additionally during embryonic development (Wallis et al., 2008) to test for reversal in this period. The resulting animals were then analyzed as adults (**Fig. 5, Table S1**). Our data unequivocally show that the postnatal reactivation of TRα1 signaling leads to an unaltered expression of the previously impaired expression of CNPase, MOG, PLP1 and ODSP when comparing the TRα1++ and TRα1+m genotypes, while the additional reactivation before birth in TRβ-/- dams has little effect. Likewise, DPYD and HCN2 were no longer lower in TRα1+m as compared to the respective controls. Interestingly, TMEM117, which was not affected by the TRα1*

© 2023. Published by The Company of Biologists under the terms of the Creative Commons Attribution License (https://creativecommons.org/licenses/by/4.0/).

mutation on the protein level, was lowered in both groups that were exposed to elevated thyroid hormone prenatally.

Feature plots (UMAPs showing the expression of an individual gene) are only shown for the top ten differentially expressed genes in Figure 3B. However, these feature plots should also be provided for all genes profiled via western blotting.
We thank the reviewer for this suggestion. We have now provided this data in the new **Fig S14B** (pasted above). We hypothesize the mismatch between the protein- and transcript-level expression to be due to translational regulation mentioned earlier.

Figure 4B,D and 5B,D: The three data points per group need to be shown individually, e.g. plotting them instead of the bar plots or plotting them on the bar plots (see also Weissgerber et al. 2015).
We thank the reviewer for this suggestion. The figures have been adapted to show the individual values on the bars.

Figure 5: It is unclear what "wildtype" refers to: Are the hypothalamus samples from WT mice or mice carrying a WT TRalpha1 gene in a TRbeta knockout background? This needs to be clarified in the figure legend and the labeling within the figures. What is presented is presumably "WT TRalpha1 gene in a TRbeta knockout"; in this case, the protein levels should also be directly compared with those of WT mice (presented in Figure 4), and the authors should commend on potential differences between these two "WT" references.
The reviewer is correct, the samples in **Fig. 5** are from animals on a TRb knockout background. We have changed the labels accordingly, namely from wildtype to TRa1++ to reflect the fact that the wildtype refers only to the TRa1 allele. We also appreciate the suggestion for a direct comparison to **Fig. 4** to identify the contribution of the TRb knockout background; however, as the samples were run on two different gels and subsequently western blots without calibrator samples (due to space constraints), we would prefer not to provide a direct comparison between the two experiments. We have added this limitation to the methods section as follows:

*As the results from **Figs 4**, **5** have been obtained from separate gel runs, there is no direct comparison between the respective groups to avoid technical bias.*

Minor comments:
We thank the reviewer for highlighting the following errors, all of which have been addressed.

Line 26: "in cellular development" Could mean anything and should be specified.
This wording has been removed.

Line 86: extend title beyond the method that is being used
The title of this section has been changed to: "*Cell type composition of the hypothalamus*"

Line 90: change to "nuclei per sequenced sample" to avoid that the numbers are misunderstood as experimental input.
This edit has been implemented.

Line 103: it should be "by differential gene expression analysis"
This error has been fixed.

Line 121:Provide percentages when referring to "this very small cluster"
This information has been added

Line 139:sentence is discontinued "batch effects since…"
The word "since" has been removed.

Line 159: "oligodendrocytes formed two distinct clusters". It seems like no additional clustering was performed and the authors should instead refer to two distinctly distributed groups of cells within the oligodendrocyte cluster.
We had previously performed a lovain-based sub-clustering of this oligodendrocyte cluster, which gave rise to (as expected) two clusters, with only a few cells that showed cross-over. Therefore, we deemed it to be more appropriate to perform DE analysis based on the genotypes instead of the subcluster identities. We have now removed references to the two groups of cells as clusters.

© 2023. Published by The Company of Biologists under the terms of the Creative Commons Attribution License (https://creativecommons.org/licenses/by/4.0/).

Line 176: “newly identified marker genes”. These are not marker genes for a specific cell type (at least no data was presented that would suggest cell type specificity) and to avoid misunderstandings the authors could consistently refer to these genes as “differentially expressed genes”.
This edit has been implemented at this line as well as at other instances throughout the manuscript.

Line 236: “TH” should be spelled out before the abbreviation is used.
The abbreviation has been introduced at this instance.

Line 325-339: Provide exact information which functions were used from which packages/versions.
The package and version information was provided at the end of the paragraph, and it has now been moved to the beginning of the respective paragraphs.

Supplementary Figure 7: The cell type compositions of the data sets are compared based on the original clustering and annotations of the respective studies (i.e. no data integration was performed before making a comparison). This should be mentioned.
We thank the reviewer for this important clarification statement. We have added this to the figure caption.

Figure 4C and 5C: Please add the missing loading controls for the Western blots.
We apologize for the oversight and added the loading controls as requested.

Figure 4B: For CNPase, please check whether the difference is indeed significant between WT and TRalpha1 mutant (the average levels appear very similar)
The p-value is 0.033 for the TRa genotype in the 2-way ANOVA analysis.

Supplementary Table 1: The meaning of the asterisk should be included in the figure legend.
This information has been added to the table legend.

Second decision letter

MS ID#: DEVELOP/2022/201228

MS TITLE: Single-cell RNA based phenotyping reveals a pivotal role of thyroid hormone receptor alpha for hypothalamic development

AUTHORS: Varun K A Sreenivasan, Riccardo Dore, Julia Resch, Julia Maier, Carola Dietrich, Saranya Balachandran, Jana Henck, Jens Mittag, and Malte Spielmann

I have now received all the referees reports on the above manuscript, and have reached a decision. The referees' comments are appended below, or you can access them online: please go to BenchPress and click on the 'Manuscripts with Decisions' queue in the Author Area.

The overall evaluation is positive and we would like to publish a revised manuscript in Development, provided that the referees' comments can be satisfactorily addressed. Please attend to all of the reviewers' comments in your revised manuscript and detail them in your point-by-point response. If you do not agree with any of their criticisms or suggestions explain clearly why this is so. If it would be helpful, you are welcome to contact us to discuss your revision in greater detail. Please send us a point-by-point response indicating your plans for addressing the referee’s comments, and we will look over this and provide further guidance.

© 2023. Published by The Company of Biologists under the terms of the Creative Commons Attribution License (https://creativecommons.org/licenses/by/4.0/).

Reviewer 1

Advance summary and potential significance to field

In the presented manuscript, Sreenivasan and colleagues use single-nucleus RNA-seq profiling to investigate defects across cell types of the hypothalamus as a function of hypothyroidism downstream of a hypomorphic allele of TR&[alpha]1.

Single-nucleus RNA-seq identified a surprisingly minor effect of hypothyroidism on gene expression of hypothalamic neuronal populations but a substantial change in hypothalamic oligodendrocytes. The authors demonstrate that changes induced in hypothalamic oligodendrocytes are due to a developmental defect where oligodendrocytes are unable to reach full maturation. In addition, the authors show that a post-natal increase in thyroid signaling can rescue this phenotype by identifying a precise window where thyroid signaling is necessary for hypothalamic oligodendrocyte development. The results of this study have implications for informing future therapeutic strategies for individuals with congenital hypothyroidism.

Comments for the author

The reviewers have adequately addressed all of my previous comments.

Minor comment:

- In the violin plots (e.g., Fig S11), it is difficult to see the distribution in the violins due to the per-cell expression values plotted on top. Making the cell points smaller or adding transparency would help.

Reviewer 2

Advance summary and potential significance to field

The authors have comprehensively addressed the points raised and improved the manuscript substantially.
The manuscript is suitable for publication.

Comments for the author

I have only three aspects that I recommend the authors adjust:

1. In the revision, reviewer 1 wondered whether the authors had performed the differential gene expression analysis on batch-corrected expression values. The authors have now added a differential gene expression analysis on Seurat CCA-batch corrected values (S10B). However, it is best practice to perform differential gene expression analysis on the original data rather than batch-corrected values. I recommend removing S10B (but keeping S10A).

2. How the TRalpha1+m mutation and the TRbeta-/- knockout relate to and affect each other still needs to be explained. The authors should describe in detail how a knockout of another receptor gene (TRbeta-/-) can rescue (here reactivate) the functionality of the mutated receptor gene TRalpha1+m. Mittag, Wallis Vennstroem 2010 (DOI 10.1007/s10741-008-9119-5) indicate that in this context supraphysiological doses of activated TH can reactivate TRa1+m.

3. The adjusted text still reads “very relevant for the completion of maturation,” which implies that the cells are stuck in the maturation process. The authors, in their response, clarified that they do not see the phenotype as “stuck in development.” Thus, they should adjust the text (e.g., “very relevant for normal maturation”).

© 2023. Published by The Company of Biologists under the terms of the Creative Commons Attribution License (https://creativecommons.org/licenses/by/4.0/).

Second revision

Author response to reviewers' comments

Our responses to the reviewer's comments in blue and any edits made to the manuscript in *"brown italics".*

Reviewer 1 Advance Summary and Potential Significance to Field:
In the presented manuscript, Sreenivasan and colleagues use single-nucleus RNA-seq profiling to investigate defects across cell types of the hypothalamus as a function of hypothyroidism downstream of a hypomorphic allele of TR&[alpha]1. Single-nucleus RNA-seq identified a surprisingly minor effect of hypothyroidism on gene expression of hypothalamic neuronal populations but a substantial change in hypothalamic oligodendrocytes. The authors demonstrate that changes induced in hypothalamic oligodendrocytes are due to a developmental defect where oligodendrocytes are unable to reach full maturation. In addition, the authors show that a post-natal increase in thyroid signaling can rescue this phenotype by identifying a precise window where thyroid signaling is necessary for hypothalamic oligodendrocyte development. The results of this study have implications for informing future therapeutic strategies for individuals with congenital hypothyroidism.

We thank the reviewer for their interest in our manuscript and the positive feedback. We have addressed all the comments raised by the reviewer, as below.

Reviewer 1 Comments for the Author:
The reviewers have adequately addressed all of my previous comments.

Minor comment:
- In the violin plots (e.g., Fig S11), it is difficult to see the distribution in the violins due to the per-cell expression values plotted on top. Making the cell points smaller or adding transparency would help.

Thank you for this suggestion. We have now modified the Fig S11-13 for better visibility of the distributions. As a representative, the modified Fig S11 is shown below.

© 2023. Published by The Company of Biologists under the terms of the Creative Commons Attribution License (https://creativecommons.org/licenses/by/4.0/).

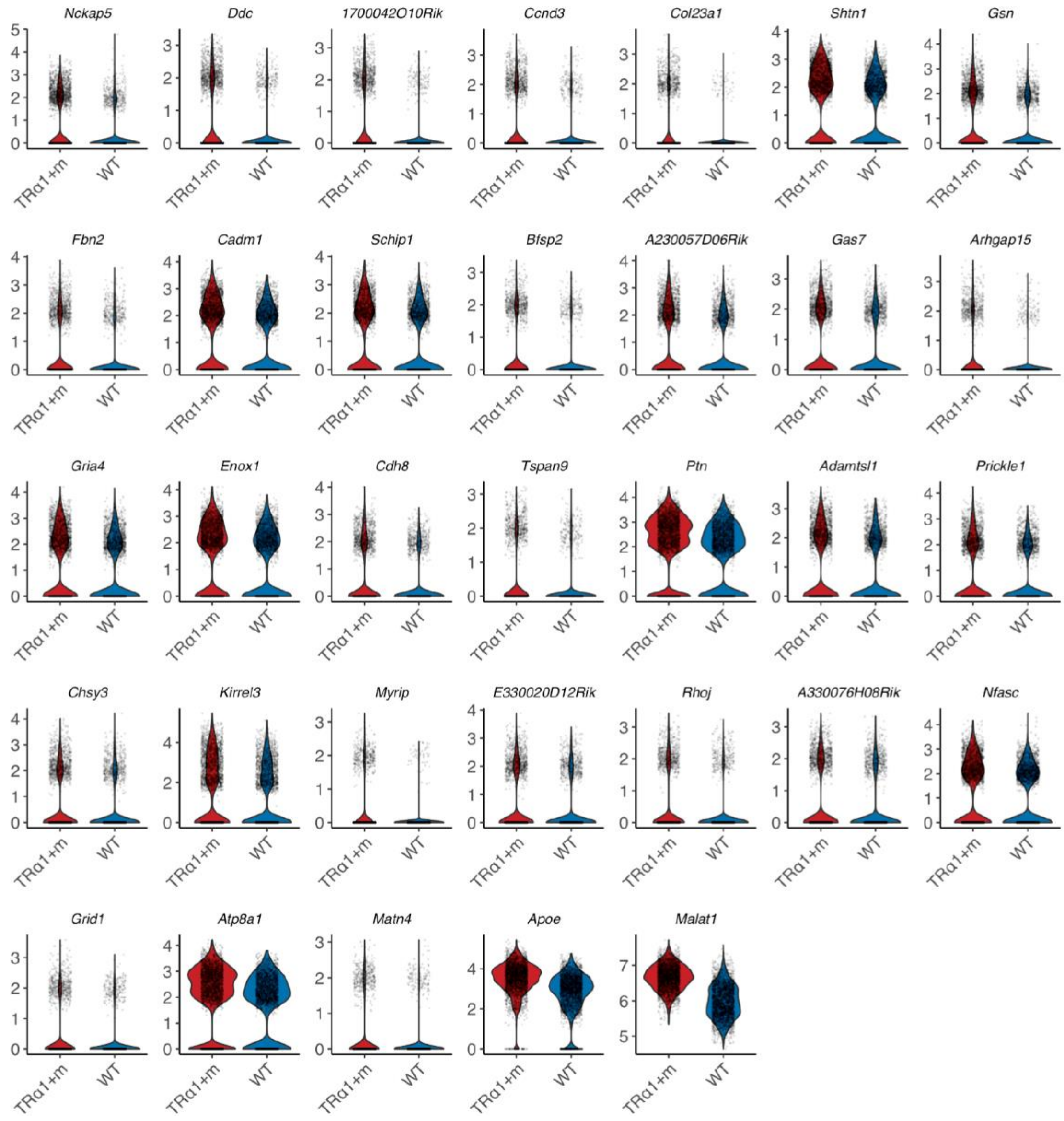

Reviewer 2 Advance Summary and Potential Significance to Field:
The authors have comprehensively addressed the points raised and improved the manuscript substantially.
The manuscript is suitable for publication.

We thank the reviewer for the positive feedback. We have addressed all the comments as below.

Reviewer 2 Comments for the Author:
I have only three aspects that I recommend the authors adjust:

1. In the revision, reviewer 1 wondered whether the authors had performed the differential gene expression analysis on batch-corrected expression values. The authors have now added a differential gene expression analysis on Seurat CCA-batch corrected values (S10B). However, it is best practice to perform differential gene expression analysis on the original data rather than batch-corrected values. I recommend removing S10B (but keeping S10A).
We have now removed the Figure S10B and all the associated text in the manuscript.

2. How the TRalpha1+m mutation and the TRbeta-/- knockout relate to and affect each other still needs to be explained. The authors should describe in detail how a knockout of another receptor gene (TRbeta-/-) can rescue (here reactivate) the functionality of the mutated receptor

© 2023. Published by The Company of Biologists under the terms of the Creative Commons Attribution License (https://creativecommons.org/licenses/by/4.0/).

gene TRalpha1+m. Mittag, Wallis, Vennstroem 2010 (DOI 10.1007/s10741-008-9119-5) indicate that in this context, supraphysiological doses of activated TH can reactivate TRa1+m.

We appreciate the opportunity to expand the explanation of the TRb KO strategy in the revised manuscript, which now reads:

> *"To this end, we aimed to reactivate TRα1 signaling before and after birth using our established approach of backcrossing the TRα1+m mice to the TR knockout strain. The TRα1+m TR -/- double knockout mice are endogenously hyperthyroid in postnatal life, as removal of TR results in an impaired feedback loop within the hypothalamus-pituitary-thyroid axis and thus elevated thyroid stimulating hormone (TSH) levels. This triggers high release of T3 and T4 from the thyroid gland and reactivates the mutant TRα1. Therefore, any reversal of a TRα1+m phenotype on the TR -/- background then confirms that it occurs postnatally and is furthermore independent of TR signaling, as this receptor has been removed in the double mutants. As a second group, we included TRα1+m TR -/- double knockouts, that are born by TR -/- knockout mothers, which are hyperthyroid throughout pregnancy and thus can reactivate the mutant TRα1 additionally during embryonic development (Wallis et al., 2008) to test for reversal in this period."*

3. The adjusted text still reads "very relevant for the completion of maturation," which implies that the cells are stuck in the maturation process. The authors, in their response, clarified that they do not see the phenotype as "stuck in development." Thus, they should adjust the text (e.g., "very relevant for normal maturation").

We thank the reviewer for this notion and changed the text as suggested to:

> *"very relevant for normal maturation"*

Third decision letter

MS ID#: DEVELOP/2022/201228

MS TITLE: Single-cell RNA based phenotyping reveals a pivotal role of thyroid hormone receptor alpha for hypothalamic development

AUTHORS: Varun K A Sreenivasan, Riccardo Dore, Julia Resch, Julia Maier, Carola Dietrich, Saranya Balachandran, Jana Henck, Jens Mittag, and Malte Spielmann
ARTICLE TYPE: Research Article

I am happy to tell you that your manuscript has been accepted for publication in Development, pending our standard ethics checks.

© 2023. Published by The Company of Biologists under the terms of the Creative Commons Attribution License (https://creativecommons.org/licenses/by/4.0/).
